# Innovative Three-Step Microwave-Promoted Synthesis of *N*-Propargyltetrahydroquinoline and 1,2,3-Triazole Derivatives as a Potential Factor Xa (FXa) Inhibitors: Drug Design, Synthesis, and Biological Evaluation

**DOI:** 10.3390/molecules25030491

**Published:** 2020-01-23

**Authors:** Fabián Santana-Romo, Carlos F. Lagos, Yorley Duarte, Francisco Castillo, Yanina Moglie, Miguel A. Maestro, Nitin Charbe, Flavia C. Zacconi

**Affiliations:** 1 Departamento de Química Orgánica, Facultad de Química y de Farmacia, Pontificia Universidad Católica de Chile, Av. Vicuña Mackenna 4860, Macul, Santiago 7820436, Chile; fmsantana@uc.cl (F.S.-R.); fjcastil@uc.cl (F.C.); nitinunimi@gmail.com (N.C.); 2 Chemical Biology & Drug Discovery Laboratory, Facultad de Medicina y Ciencia, Universidad San Sebastián, Lota 2465, Providencia 7510157, Santiago de Chile, Chile; carlos.lagos@uss.cl; 3 Center for Bioinformatics and Integrative Biology, Facultad de Ciencias de la Vida, Universidad Andrés Bello, Santiago 8370146, Chile; yorley.duarte@unab.cl; 4 Institute for Biological and Medical Engineering, Schools of Engineering, Medicine and Biological Sciences, Pontificia Universidad Católica de Chile, Av. Vicuña Mackenna 4860, Macul, Santiago 7820436, Chile; 5 Departamento de Química, Instituto de Química del Sur (INQUISUR-CONICET), Universidad Nacional del Sur Avenida Alem 1253, Bahía Blanca B8000CPB, Argentina; ymoglie@uns.edu.ar; 6 Department of Chemistry—CICA, University of A Coruña, Campus da Zapateira, 15008A A Coruña, Spain; miguel.maestro@udc.es

**Keywords:** factor Xa inhibitors, microwave-assisted synthesis, *N*-propargyltetrahydroquinoline, 1,2,3-triazole, cell viability assay, coagulation parameters

## Abstract

The coagulation cascade is the process of the conversion of soluble fibrinogen to insoluble fibrin that terminates in production of a clot. Factor Xa (FXa) is a serine protease involved in the blood coagulation cascade. Moreover, FXa plays a vital role in the enzymatic sequence which ends with the thrombus production. Thrombosis is a common causal pathology for three widespread cardiovascular syndromes: acute coronary syndrome (ACS), venous thromboembolism (VTE), and strokes. In this research a series of *N*-propargyltetrahydroquinoline and 1,2,3-triazole derivatives as a potential factor Xa (FXa) inhibitor were designed, synthesized, and evaluated for their FXa inhibitor activity, cytotoxicity activity and coagulation parameters. Rational design for the desired novel molecules was performed through protein-ligand complexes selection and ligand clustering. The microwave-assisted synthetic strategy of selected compounds was carried out by using Ullmann-Goldberg, *N*-propargylation, Mannich addition, Friedel-Crafts, and 1,3-dipolar cycloaddition type reactions under microwave irradiation. The microwave methodology proved to be an efficient way to obtain all novel compounds in high yields (73–93%). Furthermore, a thermochemical analysis, optimization and reactivity indexes such as electronic chemical potential (µ), chemical hardness (η), and electrophilicity (ω) were performed to understand the relationship between the structure and the energetic behavior of all the series. Then, in vitro analysis showed that compounds **27**, **29**–**31**, and **34** exhibited inhibitory activity against FXa and the corresponding half maximal inhibitory concentration (IC_50_) values were calculated. Next, a cell viability assay in HEK293 and HepG2 cell lines, and coagulation parameters (anti FXa, Prothrombin time (PT), activated Partial Thromboplastin Time (aPTT)) of the most active novel molecules were performed to determine the corresponding cytotoxicity and possible action on clotting pathways. The obtained results suggest that compounds **27** and **29** inhibited FXa targeting through coagulation factors in the intrinsic and extrinsic pathways. However, compound **34** may target coagulation FXa mainly by the extrinsic and common pathway. Interestingly, the most active compounds in relation to the inhibition activity against FXa and coagulation parameters did not show toxicity at the performed coagulation assay concentrations. Finally, docking studies confirmed the preferential binding mode of *N*-propargyltetrahydroquinoline and 1,2,3-triazole derivatives inside the active site of FXa.

## 1. Introduction

In the last few decades, statistics relating to a high number of cardiovascular syndromes that are a leading cause of heart problems and increasing death rates in Europe and the US have been published. A recent study carried out in Chile found the incidence risk rate for thromboembolic diseases among patients under general surgery is 55% and the principal cause of death in Chile is related to cardiovascular diseases [1,2]. With more than 24,000 deaths annually, cerebrovascular accidents (CVA) represent almost a third of all deaths [3,4] in Chile.

Thrombosis is a common causal pathology for three widespread cardiovascular syndromes: acute coronary syndrome (ACS), venous thromboembolism (VTE), and strokes [5,6]. Furthermore, the latest statistical study from the Global Burden of Diseases, Injuries and Risk Factors (GBD) showed that 25% of people worldwide die from thrombosis related events [7].

The coagulation cascade is the process of the conversion of soluble fibrinogen to insoluble fibrin that terminates in production of a clot. This process is the property of plasma where factor X (FX), which is a serine endopeptidase enzyme, plays a vital role in the conversion of prothrombin to thrombin [8]. The clot composition is composed of activated platelets, red blood cells, and cross-linked fibrin protein. A clot, which blocks the bloodstream, can produce acute myocardial infarction (AMI), an ischemic stroke, and deep vein thrombosis (DVT) [9,10,11].

In the normal circulation system, blood remains in liquid form and does not coagulate. It is now well documented that endothelium cells have an important role in homeostasis maintenance and various pathological conditions. Under healthy conditions, it not only degrades thrombus and downregulates the overall process of thrombosis, but it also promotes thrombosis when damage to the endothelium of vessels occurs. The endothelial cell produces and releases certain factors such as PGI2 (Prostaglandin I2/Prostacycline), nitric oxide (NO), and adenosine diphosphate (ADP), which acts as the antiplatelet aggregating factors [12]. Thrombomodulin or CD141 is another important integral protein expressed in the membrane of the endothelial cells. This integral protein acts as a cofactor in the activation of protein C [13]. As the name suggests it modulates the activity of thrombin to work as the protein C activator. Once activated, protein C inhibits factor Va and VIIIa and thereby inhibits the platelet aggregation and clot formation [14]. Heparan sulphate a highly sulfated polysaccharide is also expressed on the surface of endothelial cells. Its analog heparin is the most commonly use anticoagulant drug in hospitals [15]. Binding of antithrombin with heparan sulfate leads to the conformational changes in the antithrombin. These changes in the antithrombin, generates the active form of antithrombin which not only inhibits the IXa and Xa, but also inhibits the thrombin, i.e., factor IIa. Tissue plasminogen activator is another protein expressed on the endothelial cells which line the internal lumen of blood vessels [16].

**Figure 1:**
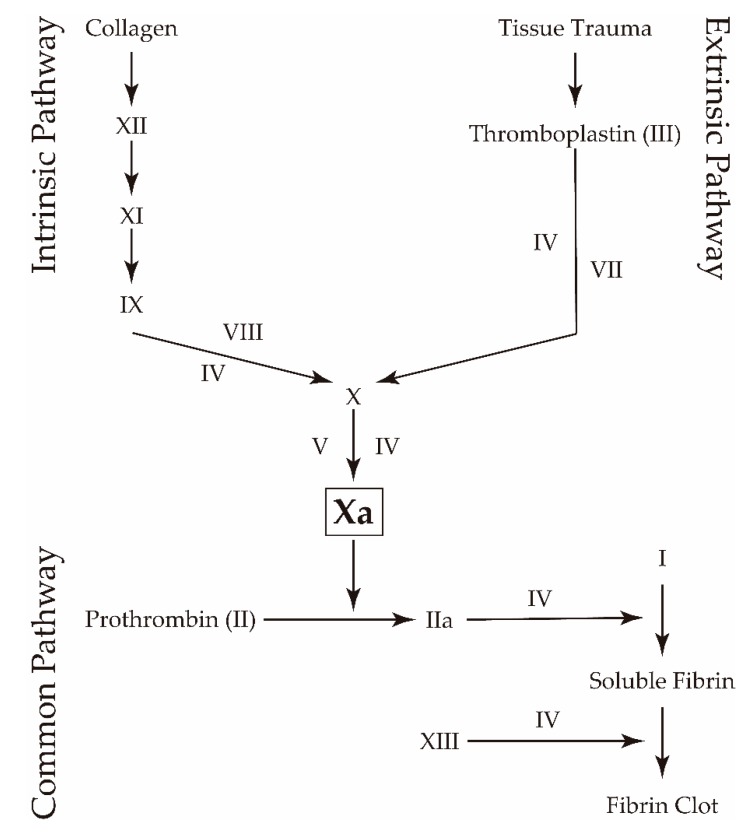
Blood coagulation cascade: extrinsic, intrinsic, and common pathway.

During an injury, at the site of action, endothelial cells lose the ability to act as an antiplatelet aggregating activity and became pro-coagulant. Injured endothelial cells release the Von Willebrand factor or factor VIII (FVIII), which is a large glycoprotein complex continuously produced by the endothelial cells [17]. VW in particular binds to the platelets to activate it and to factor VIII to begin the process of the intrinsic coagulation cascade [17,18,19,20]. Activated platelets activate the factor XII to XIIa. XIIa then activates the factor XI to XIa, which then activates IX to IXa. IXa along with Ca ions, phospholipids, and factor VIII activates factor X to Xa (FXa). FXa again in the presence of Ca ions, phospholipids, and factor V catalyzes the conversion of prothrombin to thrombin [21]. Thrombin then converts the fibrinogen to fibrin monomers [22]. Fibrin monomer is then deposited on the primary hemostatic plug, which then cross-links and stabilizes itself with the help of factor XIIIa. Tissue factor produced by the damaged endothelial cells also activates factor VII to VIIa leading to the extrinsic coagulation cascade. Factor VIIa activates factor IX and IX to IXa and Xa which are the common factors involved in the intrinsic coagulation pathway [21,22] (Figure 1).

Moreover, Factor Xa is noteworthy as the sole and common factor of intrinsic and extrinsic pathways that are involved in thrombin activation. Considering that one molecule of FXa catalyzes the formation of 1000 thrombin molecules, this enzyme plays a central role in the coagulation process of blood. Finally, this serine protease enzyme generates the main catalyzing reaction that results in clot production and wound closure [23,24]. Because of its vital role in the coagulation cascade, its inhibition is an important approach for the development of anticoagulant/antithrombic agents.

Mortality associated to thrombin is very high, and the currently available prescription drugs to prevent thrombosis in cardiovascular patients include heparins and warfarin. Rivaroxaban and Apixaban are the recently approved orally active agents available for FXa inhibition, whereas Dabigatran inhibits the thrombin directly [25]. Warfarin is the vitamin K antagonist, which, if taken incorrectly, increases the chances of dangerous bleeding. Warfarin, which is available in orally active form, has several disadvantages. It is associated with drug interactions with over the counter drugs and has been associated with severe side effects such as blood in the urine, severe stomach and joint pain, vomiting of blood, and dizziness, among others [26]. Heparins on the other hand, which can only be administered parenterally, have high interpatient variability in metabolism, and hence, require therapeutic drug monitoring for dose adjustment. Due to its short half-life and low bioavailability, frequent dosing of heparin is also essential [27,28]. Both of these agents required monitoring of clotting time and dose adjustments to prevent bleeding.

As discussed earlier, FXa is the point of junction of the intrinsic and extrinsic coagulation pathways. Inhibition of FXa could therefore be useful to inhibit intrinsic and extrinsic pathways. Furthermore, unlike warfarin, FXa inhibitors directly inhibit the critical factor involved in the cascade. The coagulation cascade is the process whereby amplification of the enzymes takes place at each step, which means the concentration of the FXa is always lower than the concentration of the thrombin. This allows for the efficient and potent antithrombotic activity of such inhibitors at very low concentrations. On the other hand, several preclinical and clinical investigations have already demonstrated a lesser risk of bleeding with the direct inhibition of FXa [22,29]. Nevertheless, the latest clinical studies have just demonstrated that Rivaroxaban and Apixaban dose interruption could produce thromboembolic events. Moreover, the utilization of warfarin combined with Rivaroxaban heightens the chance of hemorrhage in non-valvular atrial fibrillation patients [30,31,32].

**Figure 2:**
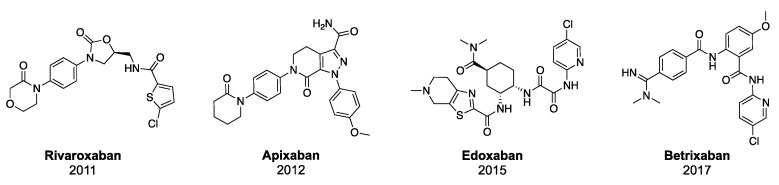
Currently clinically used FXa Inhibitors and the Food and Drug Administration (FDA) year of approval.

In recent years, several direct FXa inhibitors have become available in the marketplace, and others are in active development (Figure 2) [33,34]. Although these new oral anticoagulants are more efficacious than warfarin for the prevention of strokes and systemic embolism in patients with atrial fibrillation, the reversal agent Andexanet alfa has only recently been approved [35,36]. The development of antidotes for oral direct FXa inhibitors are still in the pipeline, but their expected approval for therapeutic purposes will be further beneficial to anticoagulation therapy [37,38].

**Figure 3:**
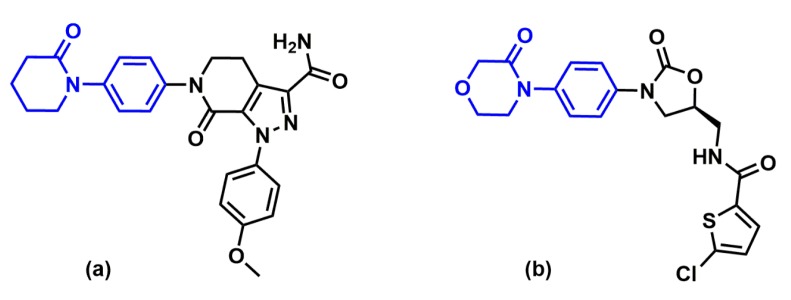
(**a**) Apixaban and (**b**) Rivaroxaban as gold standard reference compounds. The S_4_ binding scaffolds phenyloxopiperidine or phenyloxomorphonilo are highlighted in blue.

Novel FXa inhibitors under development are mainly structural bioisosteres of existing drugs (Figure 3). They show better efficacy and have a higher safety profile. Several amidines, peptide, pyrrolidines, azetidines, and triazoles are among the newly reported compounds [39,40].xd

Within the approved FXa inhibitors, the phenyloxomorphonilo or phenyloxopiperidine scaffolds present in Apixaban and Rivaroxaban are important anchoring moieties because along with the S_1_ site, S_4_ is the most important binding region for factor Xa inhibitors [39]. These groups have also shown to be pivotal for potency in that these two drugs were the first approved FXa inhibitors [33,34].

**Figure 4:**
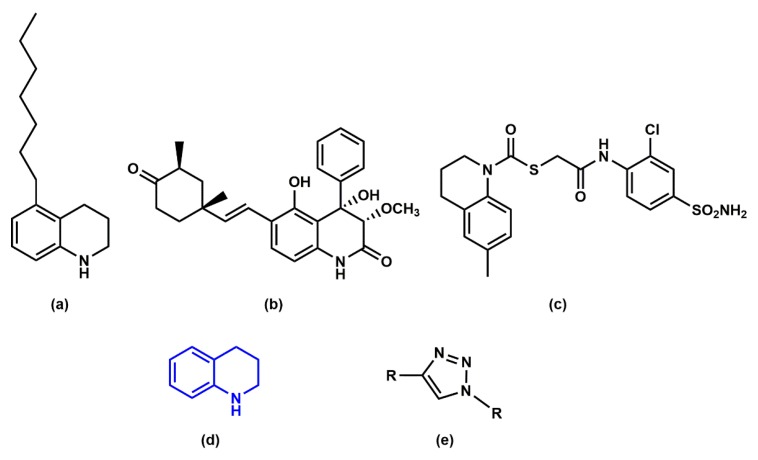
(**a**) 5-heptyl-1,2,3,4-tetrahydroquinoline [44]. (**b**) Aflaquinolone A [46]. (**c**) 1,4-disubstituted 1,2,3,4-tetrahydroquinoline [50] (**d**) Tetrahydroquinoline chemical structure. (**e**) 1,4-disubstituted-1,2,3-triazole chemical structure.

Consequently, FXa inhibitors are the critical milestones to be achieved in the clinical management of thrombosis. In recent years, various pharmacological activities of *N*-heterocyclic compounds including tetrahydroquinoline (Figure 4d) have received immense importance because of their various pharmacological activities. Heterocyclic compounds containing nitrogen not only play a crucial role in system biology but are the scaffold essential for the development of new drugs. Tetrahydroquinolines (THQs) are found to be the basic skeleton of numerous natural and synthetic compounds with various biological properties. For example, alkyl- and arylalkylderivatives of THQ such as (−)-angustureine, (−)-cuspareine, (−)-galipeine, and (−)-galipinine, which were extracted from the angostura (Galipea officinalis, Angostura trifoliata), tree have a variety of medicinal properties [41,42,43]. Different 1,2,3,4-tetrahydroquinolines with long alkyl chains at C-5 (Figure 4a) were extracted from a combined culture of Streptomyces nigrescens HEK616 and Tsukamurella pulmonis TP-B0596. These compounds were found to inhibit the growth of microorganisms [44]. Several 4-dioxygenated 3,4-dihydro 4-aryl-1,2,3,4-tetrahydroquinolin-2-(1*H*)-ones have been extracted and isolated from a variety of plants and fungi with a variety of medicinal properties [45]. For example, aflaquinolones A–G (Figure 4b), scopuquinolone B, aniduquinolones A–C, 6-deoxyaflaquinolone F, isoaflaquinolone E, 14-hydroxyaflaquinolone E, aspoquinolones A–D were also isolated from the various fungus belonging to the specie Aspergillus. These compounds have shown to have useful medicinal properties [46,47,48]. Moreover, some 1,4-disubstituted 1,2,3,4-tetrahydroquinoline derivatives (Figure 4c) were tested against HIV and few *N*-substituted tetrahydroquinoline derivatives were found to inhibit the reverse transcriptase enzyme of HIV-1 virus [49,50,51,52]. Previously, Quan et al. reported the synthesis of tetrahydroquinoline derivatives compounds having reversible factor XIa inhibitor activity in rats and rabbit thrombosis animal modes, whereas tetrahydroquinoline scaffold (Figure 4d) was assayed for its direct Factor Xa inhibition activity [52,53]. Similarly, 1,4-disubstituted-1,2,3-triazole derivatives (Figure 4e) have become an important pharmacophore due to its various biological properties and excellent stability. More recently, triazole has also been studied for its anticoagulant activity. For example, the hydrazone and sulfonamide derivatives of triazole were studied for their antiplatelet activity [54,55].

The synthesis of current antithrombotic agents is highly inefficient given that it has high costs and involves several synthetic steps, and specific catalysts. Considering the multiple-step synthesis development of commercially available FXa inhibitors [56,57], it makes the synthesis of these kind of inhibitors a challenge for the medicinal chemistry field.

Since the first reported microwave utilization techniques in organic synthesis in 1986, microwave techniques have become a recognized laboratory system and they have an important synthetic approach in the field of chemistry [58]. Moreover, these methodologies are therefore being used as an important synthetic tool [59,60,61,62,63]. For these reasons, our group has been interested in using microwave approaches to achieve our synthetic goals [64,65,66,67] in the last few years.

To this end, we have reported a modified Ullmann type procedure for related main core structures using microwave-assisted methodologies [67]. Currently, the utilization of microwave-assisted methodologies allows for the desired organic molecules in only three steps to be reached which involves Ullmann-Goldberg, *N*-propargylation, Mannich addition, Friedel-Crafts, and 1,3-dipolar cycloaddition type reactions.

Hence, in our continuous search for decisive FXa inhibitors, several approaches in designing synthesis and characterization novel FXa inhibitors [68] have been undertaken.

The present work includes the synthesis of novel tetrahydroquinoline and triazole derivatives by using microwave-assisted synthetic methodologies along with modern computer-aided drug designing and docking studies. Finally, in vitro and ex vivo assays have been performed in order to analyze the bioactivity of the molecules FXa.

## 2. Results and Discussion

### 2.1. Protein-Ligand Complexes Selection and Ligand Clustering

**Figure 5:**
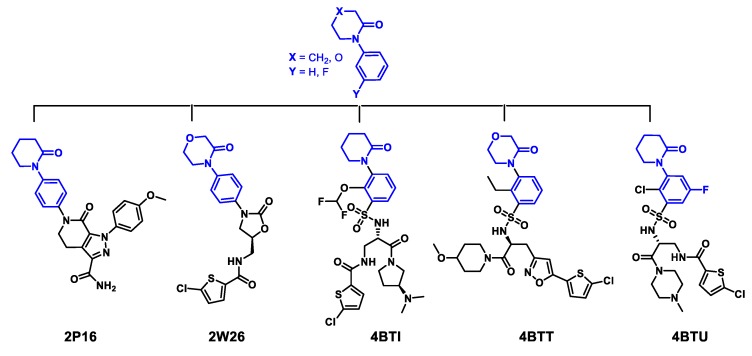
Virtual screening of interest fragment in FXa PDB crystallography data base.

First, the Protein Data Bank (PDB) (www.rcsb.org) [69] was searched for human FXa-ligand complexes available crystal structures, using the appropriate Uniprot accession number (P00742). A set of 130 enzyme-ligand reported complexes were found for humans, and after substructure search using the phenyloxomorphonilo or phenyloxopiperidine scaffolds present in Apixaban and Rivaroxaban, a set of 5 FXa-ligand complexes was compiled and their structures retrieved from the PDB (Figure 5).

After structural alignment against the protein corresponding to the cluster center (Root-mean-square deviation connected component, RMSD CC), the root-mean square deviation for the α-carbon for all residues and residues within 6 Å from the center of mass of all co-crystallized ligands was measured (Table 1).

The alpha-carbon root-mean square deviation (CαRMSD) against the low-resolution FXa-ligand structure (4BTI) ranged from 0.276 to 0.530 Å, while the all-atom RMSD within 6 Å from the center of mass of all aligned ligands ranged from 0.207 to 0.381 Å, indicating that proteins have no significant conformational differences within their binding sites in comparison with the reference structure.

The three-dimensional (3D) alignment of the ligands was used to generate a shape-based query, which was validated using a library of decoy [72,73]. The enrichment curve plots the number of active compounds recovered versus the proportion of the database screened. The AUC (area under the curve of the Receiver Operating Characteristic (ROC) plot) is defined as the probability that a randomly-chosen active compound has a higher score than a randomly-chosen inactive compound.

**Figure 6:**
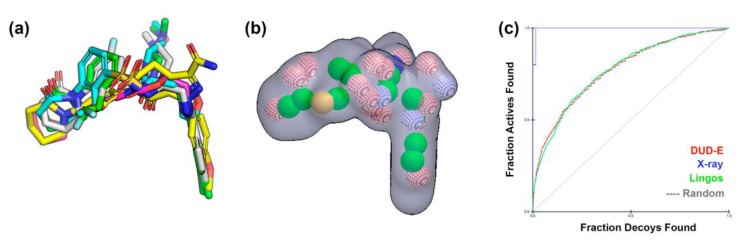
Shape-based query for phenyloxomorphonilo or phenyloxopiperidine containing FXa inhibitors. (**a**) Three-dimensional (3D) structural alignment of ligands after protein complexes superposition. (**b**) Shape-based query derived with color representing the combined shape with chemical features represented as spheres (hydrophobic = brown; rings = green; HB acceptor = red; HB donor = blue). (**c**) ROC area under the curve. HB: hydrogen bonding

The AUC of the probability obtained for the hypotheses is higher than 99% at ±95% confidence (Figure 6), suggesting the shape query hypothesis can be considered highly selective when using the actives that correspond to each cluster. However, when using a more diverse collection of FXa inhibitors from the extended database of useful decoys (DUD) [74], the AUC probability falls to near 77%. Although most actives rank higher than most of the decoy molecules, the query is considered mildly selective and only 5% of the top scoring solutions were retrieved.

**Scheme 1:**
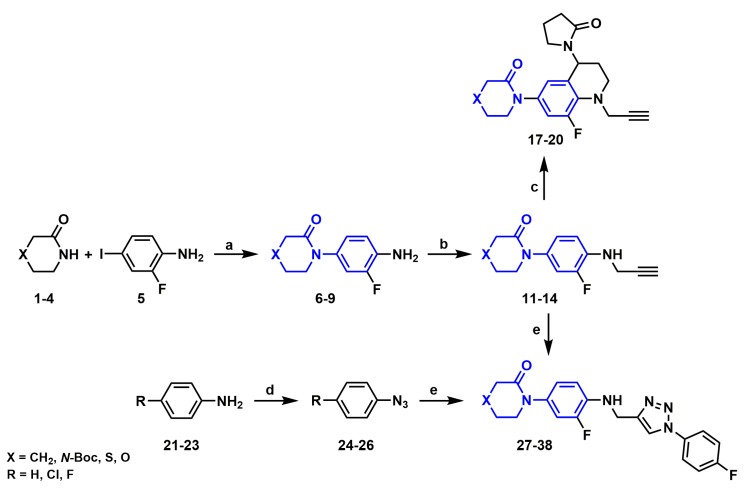
General synthetic pathway for selected compounds. (**a**) *N*,*N*′-dimethylethylenediamine (DMEDA), CuI, K_3_PO_4_, PhMe, microwave irradiation (MW), 160 °C. (**b**) propargylbromide, K_2_CO_3_, KI, CH_3_CN, MW, 160 °C. (**c**) formaldehyde, 1-vinyl-2-pyrrolidinone, InCl_3_, CH_3_CN, MW, 160 °C. (**d**) HCl, NaNO_2_, NaNO_3_, H_2_O, 40 °C. (**e**) (**11**–**14**), (**24**–**26**), copper nanoparticles supported on ZnO (CuNPs/ZnO), Et_3_N, tetrahydrofuran (THF), 160 °C.

After the generation of the shape-based query, the virtual screening protocol was applied to an in house developed library. The shape similarity between the screened compounds was evaluated by the Tanimoto Combo score method, which consists of the Tanimoto coefficient and the score retrieved from the ROCS color force field, which represents the structural complementarity between the template and the screened molecules. A final set of 28 molecules was finally prioritized and selected for synthesis (Scheme 1).

### 2.2. Synthesis of Novel Derivatives

The synthetic strategy applied for preparation of selected tetrahydroquinolines and 1*H*-1,2,3-triazole molecules was performed by using Ullmann-Goldberg, *N*-propargylation, Mannich addition, Friedel-Crafts, and 1,3-dipolar cycloaddition type reactions (Scheme 1).

#### 2.2.1. Synthetic Methodology Improvements for Aniline Precursors

Through the application of a methodology study, the performance of the Ullmann-type reaction has been improved. In previous research of our group, where the fluorine atom was not included, we observed a predictable behavior in the reaction and obtained a moderate yield, without the need to exceed the solvent boiling point [75]. In this case, applying our knowledge of the Arrhenius equation, in which the energy transmitted by microwaves affects the parameters of pressure, heating and especially time. The reaction time in all C-N couplings could be reduced for the synthesis of anilines when we increase the temperature to 160 °C [76,77,78].

**Scheme 2:**
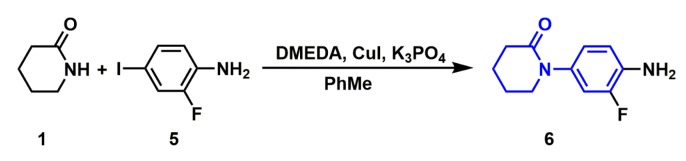
Synthesis of aniline derivative **6** [80].

In a theoretical study of C-arylations with aryl halides [79], the authors have explained the reaction reactivity between the corresponding lactams and 4-iodoaniline compound [67]. According to this, the reaction conditions were optimized by using lactam **1** (1.2 equivalents), aniline **5** (1 equivalent), CuI as catalyst and *N*,*N′*-dimethylethylenediamine (DMEDA) as ligand, in toluene as solvent using different heating sources (Table 2). The corresponding isolated compound **6** was obtained in good yield [80] (Scheme 2).

When one of the proposed mechanisms for Ullmann-Goldberg type reaction was analyzed, it was noticed that both the catalyst and the ligand bind directly to the lactam in the intermediate activation step [81]. This premise allowed us to change the reaction stoichiometry, changing the limiting reagent to lactam **1**, obtaining satisfactorily 62% yield of isolated compound under microwave heating conditions. This modification resulted in a 12% higher yield of the starting materials than previously reported yields (Table 1, entry 19).

**Scheme 3:**
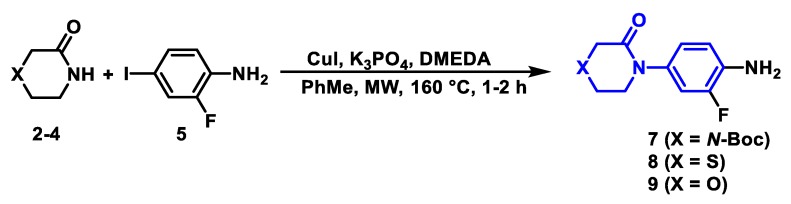
Synthesis of Anilines Derivatives (**7**–**9**) [80].

Although for piperazinone, morpholinone and thiomorpholinone derivatives, the synthesis was carried out with a lower catalyst loading (0.5 equiv.) and ligand (0.5 equiv.) (Scheme 3).

The aniline derivatives **7**, **8,** and **9** were obtained in excellent yield (90%, 86%, and 94%, respectively) of isolated products under microwave reaction conditions in 1–2 h. However, the coupling reaction for compound **7** was carried out at 90 °C, due to the deprotection side reaction. This methodological improvement allowed us to produce these interesting block buildings anilines in excellent yields, reducing reaction time and minimizing the energy consumption by using microwave technologies.

#### 2.2.2. Synthesis of *N*-propargylanilines

**Scheme 4:**
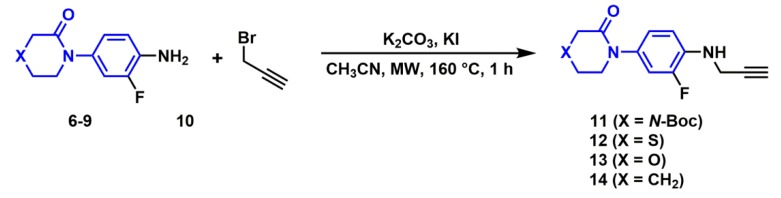
Synthesis of *N*-allyl/propargyl aniline derivatives (**11**–**14**) [80].

In the next step, *N*-propargyl anilines were synthesized according to the procedure developed by Rasool et al. with minor modifications such as temperature, time reaction and heating source (Scheme 4) [82]. The reaction conditions were optimized using different heating sources (Table 3). The best results were achieved using the aniline derivatives (**6**–**9**), propargyl bromide, K_2_CO_3_ as base, KI as additive, in acetonitrile as a solvent under microwave irradiation at 160 °C. *N*-propargyl aniline derivatives (**11**–**14**) were obtained in good yields of isolated products (62–72%). However, the synthesis of compound **13** had to be carried out at 90 °C, due to the deprotection side reaction [83,84,85,86].

Noteworthy, the propargyl derivatives were obtained with the lower yields of isolated compounds using room temperature (96 h, 9–19% yield), sonication (40–80 °C, 4–15 h, 39–67%), and conventional heating sources (80–90 °C, 24–72 h, 15–49%). In contrast, the microwave procedure showed the highest products yields (67–72%) within 30 min of reaction at 160 °C. As a second reaction step, the microwave methodology allowed us to produce the propargyl derivatives in an efficient and environmentally friendly approach.

#### 2.2.3. Synthesis of *N*-Propargyl Tetrahydroquinolines

**Scheme 5:**
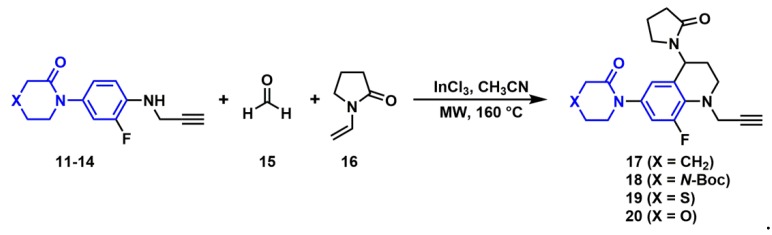
Synthesis of *N*-propargyl tetrahydroquinoline derivatives (**17**–**20**).

Different *N*-propargyl tetrahydroquinolines (**17**–**20**) were synthesized using acid-catalyzed three-component cationic imino Diels–Alder reaction [87,88,89] (Scheme 5).

The reaction conditions optimization involved the modification of the heating source [90,91].

Thus, the total reaction time was reduced from 96 h to 15 min (Table 4) through microwave-assisted methodology and compound yields were 2.5-fold increase in comparison with conventional procedures. It must be considered that compound **18** was not obtained, only decomposition products were obtained [87]. Novel tetrahydroquinolines derivatives **17**, **19,** and **20** were obtained in excellent yields (83–89%) of isolated products.

#### 2.2.4. Synthesis of Aryl Azides

**Scheme 6:**
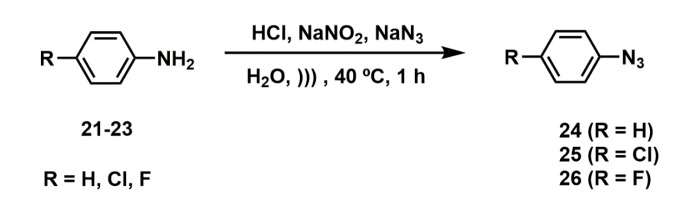
Synthesis of aryl azides derivatives (**24**–**26**).

The synthesis of the aryl azides were based on the reaction of diazonium salts with sodium azide in water under sonication at 40 °C (Scheme 6) [92]. The corresponding products were obtained in quantitative yields and they were used without further purification.

#### 2.2.5. Synthesis of 1*H*-1,2,3-triazole derivatives

**Scheme 7:**
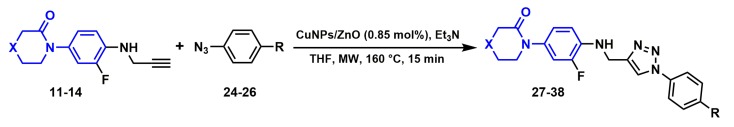
Synthesis of 1*H*-1,2,3-triazole derivatives (**27**–**38**). Compounds **27**–**29** (X = CH_2_; R = H, Cl, F, respectively); **30**–**32** (X = *N*-Boc; R = H, Cl, F, respectively); **33**–**35** (X = S; R = H, Cl, F, respectively); **36**–**38** (X = O; R = H, Cl, F, respectively). Novel 1*H*-1,2,3-triazole derivatives (**27**–**38**) were obtained in good to excellent yield (73–93%) using a lower loading of catalyst and in shorter reaction time (15 min) than previously reported by assisted microwave methodologies.

The synthesis of novel triazoles were prepared by the 1,3-dipolar cycloaddition between the *N*-propargyl amines (**11**–**14**) and the organic azide (**24**–**26**) catalyzed by copper nanoparticles supported on ZnO in tetrahydrofuran as solvent, triethylamine as base at 160 °C under microwave irradiation (Scheme 7) [93].

### 2.3. Theoretical Study

In order to understand the relationship between the structure and the energetic behavior of all the series, including a thermochemical analysis associated to the yield of the reactions, an optimization and reactivity indexes calculation of electronic chemical potential (μ), chemical hardness (η), and electrophilicity (ω) was performed.

The electronic chemical potential (μ) has the same significance as the classic thermodynamics chemical potential but characterizes the tendency to escape of electrons from the equilibrium. This index its associated to the ionization potential and the electron affinity, and to the energies of Frontier Molecular Orbitals (FMOs) based on the Koopman’s theorem.

The chemical hardness (η) is associated to the stability of the systems and the resistance of a molecule to exchange electron density. The same approximations as for electronic chemical potential were made.

For electrophilicity (ω), a higher index value indicates a more electrophilic character for the molecule, as a lower index value, a more nucleophilic character has the molecule. Electrophilicity can also be associated with the energy stabilization of a molecule [79,94].

#### 2.3.1. X-Ray and Theoretical Optimization Correlation

Structural parameters of molecules **6**, **9,** and **20** were optimized and compared with crystallographic data (Table 5, Table 6 and Table 7). The structures are well refined, with final R indices (I > 2σ(I)) of R_1_ = 0.0461, wR_2_ = 0.1292 for **6**, R_1_ = 0.0343, wR_2_ = 0.0944 for **9** and R_1_ = 0.0518, wR_2_ = 0.1552 for **20**. The crystallographic data and structure refinement can be found in the Supplementary Material (Tables S27 to S29).

For the all studied molecules, bond angles and distances show a good correlation between calculated and experimental, suggesting good robustness for the calculated structure and parameters, with no significative difference between bond lengths.

Regarding bond angles for molecule **6** (Table 5), the most significant difference occurs in the dihedral angles, with a difference between 3 to 5 degrees, showing that the molecule has a slightly different conformations around the central C(6)-N(1) atoms, and a slightly more complex twist in the non-aromatic ring.

For molecule **9**, in addition to the good length correlation, between theoretical and experimental, there was a favorable correspondence between bond angles (Table 6). The main difference takes place in the O(1) oxygen atom position, the calculations showed that this atom is facing the same plane as the fluorine F(1) atom. On the other hand, the crystal shows that the atoms are opposite from each other. The C(10)-C(5)-N(1)-C(2) dihedral angle was measured in order to visualize the difference between experimental and calculated structures (Figure S30, Supplementary Material). This behavior could be explained due to the hydrogen bonds interactions generated between the F(1) and O(2) atoms with the opposite hydrogen atoms in the molecule (Figure S31, Supplementary Material).

Bond angles for molecule **20** also shows that the most significant difference between calculated and experimental occurred in the dihedral angles with a slight difference of 5 degrees in the twist structure on the non-aromatic ring but a large difference between the propargyl moiety position and the tetrahydroquinoline cycle. The last three angles shown (Table 7) were measured to demonstrate the different positioning of the tetrahydroquinoline cycle and the propargyl moiety. This could be due to the interactions generated between the propargyl and the carbonyl groups in the crystal (Figure S32, Supplementary Material).

#### 2.3.2. Energetic Behavior and Thermochemistry

The electronic chemical potential for propargyl precursors **11**–**14** shows a mean of −3.0471 eV, with a maximum of −2.889 eV for molecule **11** and a minimum of −3.169 for molecule **13**. For the arylazide compounds (**24**–**26)**, the mean is −4.4049 eV, with a maximum of −4.277 eV for molecule **24** and a minimum of −4.490 eV for molecule **26**. This implies that the reaction between precursors **11**–**14** and **24**–**26** present a medium polar character, and the electron density flux will take place from the propargylated molecule to the arylazide [94].

The Density Functional Theory (DFT) study for 1*H*-1,2,3-triazole derivatives showed that the highest chemical hardness is obtained in the molecules synthetized from precursor **24**, thus being the more stable ones (average 2.013 eV). It is worth to notice that these derivatives (**27**, **30**, **33,** and **36,** all from precursor **24**) had the highest yield of all synthesized compounds (91.11 ± 1.55%).

For the electrophilicity index, propargyl molecules **11**–**14** showed mean of 1.8301 eV, with a maximum of 1.908 eV for molecule **14** and a minimum of 1.667 for molecule **11**. For arylazide compounds **24**–**26**, the mean is 3.4799 eV, with a maximum of 3.657 eV for molecule **27** and a minimum of 3.234 eV for molecule **25**. According to these values, propargyl precursors acts as nucleophiles and the arylazide precursors acts as electrophiles. This approach may not be entirely correct because the arylazide is a dipolar compound and the propargyl is a dipolarophile center. The reaction should go through a Huisgen cycloaddition, following a concerted mechanism with a nucleophilic attack to and from the propargylated molecule [96]. All energies for reactivity indexes and FMOs are summarized in Table 8.

In order to understand the electrophilic behavior of arylpropargylated and arylazide precursors, the Fukui functions for molecules **13** and **24** were calculated. These molecules were chosen as an example due therefrom compound **33** was obtained. This reaction presented the best performance according to the products yield.

Fukui functions helped us to understand the behavior of chemical reactions providing information on the local reactivity of atoms within the molecule, as shown in the Supplementary Material (Table S33 and S34). According to the *f* ^+^ function, N(9) nitrogen atom (molecule **24)** was the most susceptible atom to experience a nucleophilic attack. However, the most susceptible atom for compound **13** was the C(18) carbon. For neutral or radical reagents or reactions, the *f* ^0^ function can be used. In this case, with a concerted mechanism, the function shows the same behavior previously discussed [97]. The calculations predicted that the mesomeric structure that reacts is the one with a triple bond between the nitrogen atoms (Figure S35, Supplementary Material).

DFT calculations have been previously performed on similar systems, showing that the mechanism for the copper (I) catalyzed cycloaddition are in concordance with experimental and theoretical findings reported in this work. Moreover, previously published calculations also agrees with the mesomeric structure that actually reacts, showing that the reaction with and without catalyst should undergo the same mechanism, with the same sites being susceptible to nucleophilic attack [98].

The thermochemistry of the series was studied taking into consideration the sum of electronic and thermal enthalpies for both precursors and final compounds, in order to calculate the ΔH for each reaction (Table S36, Supplementary Material). All reactions have shown a highly exothermic behavior with a reaction enthalpy value of between −61.765 ± 0.295 kcal/mol. These energies were in agreement with previously calculated similar compounds [98]. It is important to note the most exothermic reaction is the one to obtain **33** (from **13** and **24**) in accordance to its highest yield of reaction.

### 2.4. Biological Analysis

#### 2.4.1. FXa Inhibition in Vitro Assay

By using SensoLyte^®^ 520 Factor Xa Assay Kit “Fluorimetric” FXa (bovine isolated enzyme) inhibition was measured. Upon FXa protease cleavage, this substrate generates the Rh110 (rhodamine 110) as free fluorophore, which can be detected at excitation/emission of 490 nm/520 nm, where the following values of inhibition FXa percentage and IC_50_ from the representative compounds of each series (compounds **6**–**38**, Table 9), as well as for all final 1*H*-1,2,3-triazole derivatives [68,99] were obtained.

The assay showed that only five of the novel compounds (**27**, **30**, **31**, and **34**) exhibited inhibitory activity higher than 50% against FXa at 100 μM. According to these results, IC_50_ values were calculated using corresponding dilutions of each sample (Materials and Methods). It is important to note that none of the evaluated compounds showed fluorescence quench at the assayed wavelength avoiding the possibility to present a potential pan-assay interference compounds (PAINS) fragment [100,101,102].

#### 2.4.2. Anti FXa assay, Prothrombin Time (PT) and Activated Partial Thromboplastin Time (aPTT) ex Vivo Assay

Ex vivo anticoagulation efficacy assays of compounds **27, 30, 31, 34,** Apixaban, and Rivaroxaban (control samples) were evaluated by anti FXa (Table 10), activated partial thromboplastin time (aPTT) and prothrombin time (PT) assays by using a human plasma pool.

We know that chromogenic FX assay measures the amount of FX activity in a patient, however, chromogenic anti-FXa activity assay does not measure the patient’s FX. What it does instead is that it measures the ability of the patient’s plasma to repress exogenous FXa. The chromogenic anti-FXa assay is therefore used to determine the concentration of anticoagulants that inhibit FXa.

What will need to be taken into consideration in the future is how anti-FXa monitoring will need to be increased. As more anticoagulants that are FX inhibitors are more freely available in the market, therapeutic compliance, together with diagnosing the causes of bleeding in a patient being treated with these anticoagulants, will need to be done much more often [103]. Widespread use of FXa inhibitors may require performing the ant-FXa assay as a complementary assay. If FXa inhibitors are more commonly used, this may mean that the anti-FXa assay as complementary assay will need to be done.

As an example, high anti-FXa levels demonstrated the increasing risk of bleeding and low levels of anti-FXa established potential thromboembolic future events.

**Figure 7:**
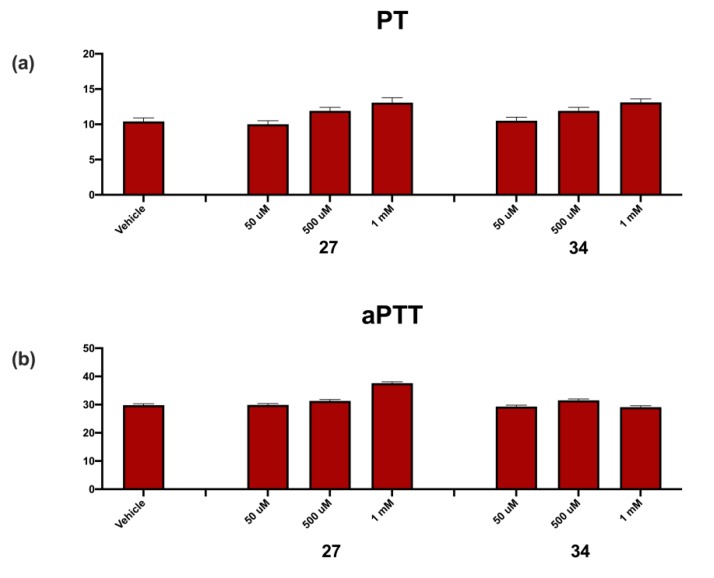
(**a**) PT and (**b**) aPTT coagulation parameters for compounds **27** and **34**.

According to the therapeutic range of the anti-FXa obtained values (0.3–0.7 IU/mL), selected compounds **27** and **34** were assay for PT and aPTT coagulation parameters (Figure 7).

PT parameter evaluated the extrinsic and common coagulation pathway. On the contrary, aPTT parameter evaluated the intrinsic pathway and its less sensitive to the final common pathway. Compound **27** simultaneously prolonged aPTT and PT in a dose-dependent manner for concentrations up to 50 μM (Figure 6a,b). Meanwhile, compound **34** only prolonged PT in a dose-dependent manner (Figure 6a) for concentrations up to 50 μM. Apixaban and Rivaroxaban prolonged both coagulation parameters in a dose-dependent manner for concentrations up to 0.1 nM [104,105].

These interesting results suggest that compounds **27** inhibited FXa targeting this coagulation factor through both pathways in order to increase the possible potency of their derivatives. Finally, compound **34** may target coagulation FXa mainly by the extrinsic and common pathway.

#### 2.4.3. Cell Viability Assays

**Figure 8:**
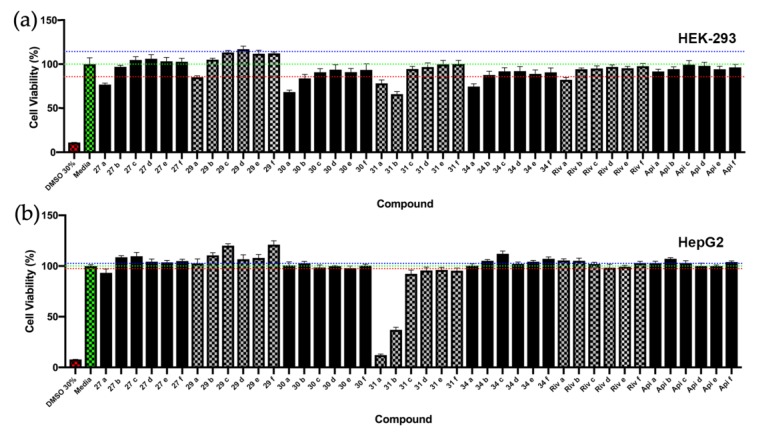
Effect of selected compounds on cell viability using the MTS assay in (**a**) HEK293 and (**b**) HepG2 cell lines. Concentrations used were 100 mM (a), 10 mM (b), 100 nM (c), 50 nM (d), 10 nM (e), and 1 nM (f). Blue and red lines defines ± 2 SD limits from the average value of control wells treated only with vehicle (green line). All values are means ± S.D (*n* = 6) vs. control. Api: Apixaban and Riv: Rivaroxaban. SD: standard deviation.

Cell viability assay was performed in HEK293 and HepG2 cells using the 3-(4,5-dimethylthiazol-2-yl)-5-(3-carboxymethoxyphenyl)-2-(4-sulfophenyl)-2H-tetrazolium (MTS) method (Figure 8). The compounds were assayed under increasing concentrations, namely, 1 nM (a), 10 nM (b), 50 nM (c), 100 nM (d), 10 mM (e), and 100 mM (f). In HEK293 cells, most of the assayed compounds only shows signs of toxicity (defined as the value obtained after subtracting two standard deviations from the average value of control wells treated only with vehicle) when concentrations are higher than 10 mM, such as compounds **30** and **31**. Compound **31** showed a greater toxic effect in HepG2, reducing cell viability to 40% and 20% of control at 10mM and 100mM, respectively, while the rest of the compounds did not affect HepG2 cell viability.

Interestingly, the most active compounds in relation to the inhibition activity against FXa and coagulation parameters **27** and **34,** did not show toxicity at the performed coagulation assays concentrations (1–500 μM).

### 2.5. Computational Analysis

#### 2.5.1. Docking Studies

Molecular docking and MM-GBSA studies (Molecular Mechanics-Generalized Born Surface Area) were performed to get insight into their binding modes and affinity of the most active compounds of each series (**8**, **19**, **27**, **30**, **31,** and **34**) against factor Xa enzyme (FXa), In this study, the Glide module of Schrödinger suite was used to find the suitable orientation of the most active compounds in the active site FXa. Comparison of the docking poses of compounds of each series and apixaban showed some similar interactions.

An overlay of the Apixaban (green) and *N*-propargyl- tetrahydroquinoline (THQ) (compound **19**) on the active site of FXa can be seen in Figure 8a. The Apixaban fit in the FXa binding site, standing between Y99 and F174 so that the phenyl lactam portion can be parallel to residue W215. Carbonyl group of the 2-oxopiperidine ring in Apixaban presents HB interaction with F174 through the water molecule. Docking analysis also revealed that the central pyridine ring of FXa inhibitor is located strategically to form HB with G216 and Y99; this last interaction is water-mediated.

**Figure 9:**
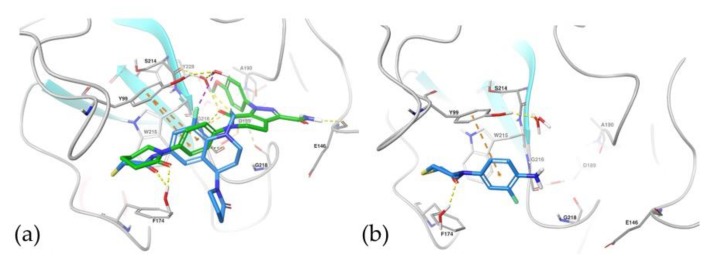
(**a**) Overlay of Apixaban (green) and compound **19** over active site of FXa. (**b**) Aniline derivative **8** on FXa.

**Figure 10:**
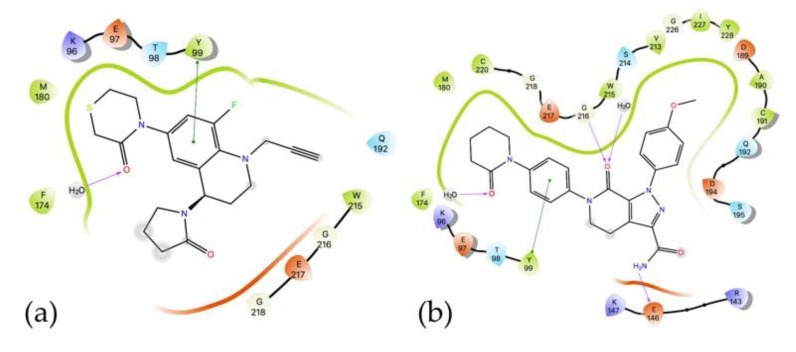
(**a**) Interaction diagram of Compound **19** in the FXa active site. (**b**) Apixaban interactions with different residues in the FXa active site.

On the other hand, the electron-rich nature of the phenyl ring of the aromatic residues F174, Y99, and W215 surrounded the ring 6-membered of the molecules **8** and **19**, forming a well-defined π-π stacking interaction with residue Y99. (Figure 9a,b). This orientation is similar to the benzene ring of apixaban, which forms the well-defined π-π stacking interaction with the phenyl ring. Similarly, the oxygen of the lactam group of thiomorpholine moiety of compounds **19** and **8** forms HB interaction with a water molecule close to residue F174 at 3.00 Å (Figure 10a). The interaction with F174 is comparable to presented by 2-oxopiperidine ring of apixaban. Additionally, near the bottom of the FXa active site, the propargyl group of THQ **19** forms two HB interactions, one of them with residue G216 at 3.22 Å, and other with the residue Y99 water-mediated. This interaction is like the interaction of the carbonyl group of the piperidine ring of apixaban (Figure 10b).

In the complex FXa-compound **19**, it was possible to observe one water-mediated halogen bond (dotted line in magenta) between the fluorine atom of THQ with the aromatic residue Y99 at a distance of 3.02 Å [106,107]. Although fluorine is a highly electronegative element, it could form a noncovalent bond with another electron donor when fluorine itself is linked with the sufficiently electron attracting group. In such an interaction, fluorine acts as an electrophilic group. Such interactions are known as the halogen bonding or fluorine bond. The halogen (fluorine) bonding has the property of stabilizing inter- and intramolecular interactions that can preserve ligand interactions and can affect molecular folding [108]. In pharmacology research, several halogenated compounds as drugs have been included, but this interaction is rarely considered necessary for the rational drug design process [109]. Thereby, potential interactions as described above could explain the inhibitory potency of the compound **19**. The structural difference of compound **19** with respect to the other THQ derivatives was the presence of sulfur atom on the morpholine scaffolds. The sulfur atom on the thiomorpholine ring did not appear to interact with any specific residue in this region of the enzyme, and it was oriented to the exposed hydrophobic region solvent.

On closer examination of the docking binding modes, binding differences between *N*-propargyl-THQ and apixaban inhibitors (Figure 10a) were better understood. Compound **19** lacked some fundamental intermolecular interactions that were established by Apixaban, which could explain the difference in activity. As an example, apixaban formed an H-bond with negative charged residue E146 through its amine terminal group, while compound **19** was incapable of forming such an interaction. Moreover, the docking poses show that a methoxyphenyl ring of Apixaban spread through the active site cavity close to residues C191 and G216, while THQ was not large enough to reach the site of the enzyme. Other interactions that compound **19** were missing was the HB interaction of the pyrazole ring of the Apixaban with residue G216 at distance 2.9 Å [71]. Unlike that of Apixaban and compound **19**, the aniline derivative **8** presented a small chemical structure that did not allow for it to build a good cover of the binding site, evidencing the low biological activity against FXa enzyme.

**Figure 11:**
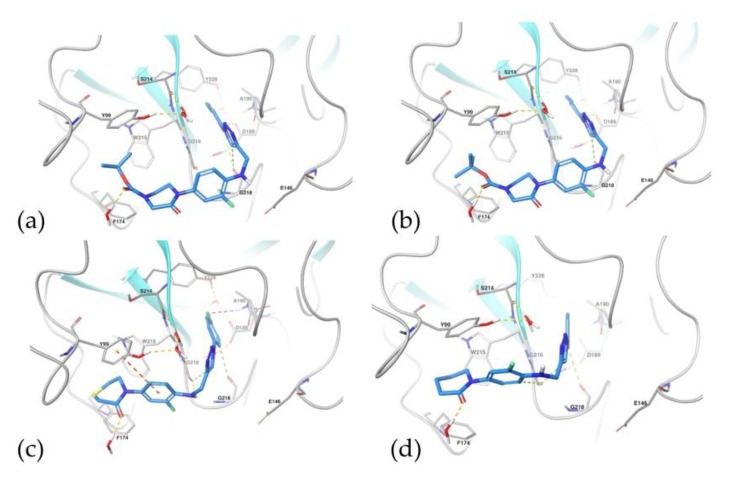
Molecular docking of structures **31**, **30**, **34**, and **27** ((**a**), (**b**), (**c**), and (**d**), respectively) into the active site of FXa enzyme (PDB: 2HYY). The dotted lines show the most common ligand-protein interactions in all ligand binding poses, HB in yellow, π-π stacking in orange, halogen bond in magenta and CH···O HB in green.

Unlike compounds **8** and **19**, four triazole derivatives (**31, 30, 34** and **27**) showed a marked biological activity against FXa enzyme. These compounds displayed a similar orientation to Apixaban in the binding site and similar molecular interactions dominated by CH···O hydrogen bonds (HB) and π-stacking interactions with residues Y99, G218, D189, and G216, as shown in Figure 11. CH···O hydrogen bonds are well-recognized interactions in protein structures. These interactions are energetically stabilizing and play a fundamental role in several biological processes such as in the protein structure and their folding until enzyme catalysis [109]. Several studies have shown that carbon atoms can form weak hydrogen bonds, which are denoted as CH···O hydrogen bonds. Due to the increase of polarization by the adjacent atoms, carbon atoms can theoretically participate in hydrogen bonds as strong as those formed by conventional donors, like oxygen or nitrogen, thus, this type of interaction is relevant to the study of molecular docking [110,111].

At the molecular level, compound **31** presented the highest biological activity (29.7 μM), as it occupied most of the active site of FXa exhibiting several molecular interactions. Specifically, the triazole and chlorobenzene rings showed CH···O HB interactions with G218 and D189 residues with distances of less than 3.71 Å. The fluorobenzene central ring established CH···O HB interactions with G218, and the Boc terminal moiety formed the same kind of interactions with the Y99 residue, with distances of 2.47 and 3.90 Å, respectively. The second and fourth most active compounds, **30** and **27** in the series, presented similar molecular interactions to compound **31**, highlighting the interactions with G218 and G216 residues, mainly mediated by CH···O HB interactions. Furthermore, the aromatic interaction with Y99 residue was maintained with a Boc moiety of compound **30**. Compound **34**, the third in biological activity, was oriented entirely into the active site of the enzyme, presenting molecular interactions not shown by the other compounds of the same series. The structural characteristics of this molecule, such as the absence of the Boc protective group, allowed it to interact by well-defined π-π staking between the fluorobenzene ring and the Y99 residue. At the same time, the terminal chlorobenzene ring was positioned in a way that allowed it to establish a halogen bond with A190 at 3.33 Å.

The predicted Glide Emodel values obtained from molecular docking for compounds **8, 19, 27, 30, 31,** and **34** are listed in Table 6. Emodel energy values agree with the values inhibitory activities [112,113]. Compound **19** bound more strongly to FXa than compound **8**, but it was weaker than the triazole derivatives (−56.62 and −45.66 kcal/mol for **19** and **8**, respectively, and a range of −77.10 to −82.94 kcal/mol for triazole derivatives). The inspection of the Glide SP scoring terms revealed that the Van der Waals (VdW) interaction energy was the major contributor to the inhibitor binding. The VdW contribution had higher values for triazole derivatives than for compounds **19** and **8**. Glide Emodel and VdW contribution predicted the correct ranking of the compounds.

MM-GBSA calculations using the module Prime MM/GBSA [114,115] were also performed. This was done to get an estimation of the energetic differences observed in the binding affinity and to understand better the thermodynamic factors involved in differential FXa biological activities of the more active compounds. The MM/GBSA results showed that compound **19** and **8** have energy values of −53.15 and −49.49kcal/mol, respectively, while the calculated values for triazole derivatives were in the range of −52.84 to −67.39 kcal/mol. The ΔG_bind VdW_ term often dominates the net ΔG_bind_, and its contribution in this study is relevant in that it is the highest value for compound **31**, which was the compound that showed the highest biological activity. This result indicated that VdW interaction is the driving force for the inhibitor binding, and the Boc group interactions of compound **31** has contributed to this term. Unfortunately, the ΔG_bind_ calculations contained one vague approximation that has affected our results.

The approximation is related to the lack of conformational entropy and information about the number and free energy of water molecules in the binding site [114]. The water molecules were key in many of the electrostatic and H-bond interactions in enzyme-ligands complexes, and the lack of inclusion of their contribution has affected the ΔG_bind_ results.

The molecular docking and MM/GBSA calculations are an interesting approach to try to explain or predict the activity of the different chemical derivatives in a FXa enzyme. Moreover, the different binding patterns of the best molecules proposed in this study can help us to better understand at a molecular level the modes of interaction of the new series of synthesized compounds (Table 11).

#### 2.5.2. Molecular Dynamics Simulations

The generated protein-ligand complexes from more active molecular docking compounds (*N*-propargyl-THQ, **19** and 1,4-disubstituted-1,2,3-triazole derivative, **31**) were subsequently submitted to MD simulations to optimize the ligand position into the FXA binding site using Schrödinger software suite [116]. In comparison with the initial docking position of compound **19**, it showed an accommodation slightly different in the binding pocket throughout the MD simulation time. In particular, the initial π-π staking interaction with Y99 was maintained for a significant part of the time of the simulation. Indeed, the aromatic ring of the THQ compound showed an accommodation parallel to Y99 and W215 settling two stables π-π stacking interactions with both residues. The interaction with W215 was the most frequent. The pyrrolidone ring kept in constant rotation, established an interaction network of hydrogen bonds with residues E146 and G216. Similarly, the morpholine moiety presented some unstable hydrophobic interactions with F174 as well as aromatic-HB interactions with Y99. Despite the high degree of rotation of the THQ molecule, the strong hydrophobic interactions with the aromatic residues W215, Y99, and F174 did not allow this molecule to disperse from this part of the active site, demonstrating some stability in this region of the protein. On the other hand, the analysis of the MD trajectory of compound **31** revealed π-stacking interactions between the Boc moiety and Y99 residue for much of the trajectory time. In addition, a moderately frequent hydrophobic interaction was observed between the same Boc moiety and the ring of F174. The morpholine fragment displayed a high-frequency of hydrophobic interaction with W215, while chlorobenzene ring revealed low-frequency of CH···O HB interactions with D189 and G218, as well as a low presence of HB interactions in the protein-compound **31** complex. MD analysis shows that Y99, W215, and F274 residues contribute significantly to the stabilization of compound **31** within the FXa binding site.

The RMSD values of the backbone atoms in the protein show a convergence in the first nanoseconds (ns) of simulation, showing fluctuations between 1.0–1.8 Å. The low RMSD indicates that the FXA protein in complex with compounds **19** and **31** presents a stable conformation during the MD simulation.

#### 2.5.3. In Silico Pharmacokinetics Prediction

The potential of novel compounds to cross the blood–brain barrier via passive diffusion was evaluated by molecular descriptors: the topological polar surface area (TPSA), the calculated logarithm of the octanol/water partition coefficient (logP) (Table 12) [117,118]. On the other hand, several parameters were calculated in parallel, the molecular weight (MW), the number of hydrogen bond acceptors (HBA), the number of hydrogen bond donors (HBD), and the number of rotatable bonds (RB). Molecular descriptors were determined using QikProp of Schrödinger [119]. QuikProp allows you to compute physicochemical descriptors as well as to predict absorption, distribution, metabolism, and excretion (ADME) parameters, pharmacokinetic properties, druglike nature, and medicinal chemistry friendliness of one or multiple small molecules to support drug discovery [120].

Taking into account the in silico pharmacokinetics results for compounds **27** and **34**, they showed logP < 5, appropriate logBB, logS and PHOA values that encourage us to utilize these novel molecules as scaffolds for potential anticoagulant oral administration drugs.

## 3. Materials and Methods

### 3.1. FXa-Ligand Structure Retrieval and Standardization

The reference sequence for human FXa was obtained from the Uniprot database (entry code P00742). The protein databank PDB advanced search tool was used to BLAST the sequence and identify all available human FXa crystal structures. All small co-crystallized ligands were retrieved in Simplified Molecular-Input Line-Entry System (SMILES) format for the substructure search. It was done as a pdb file for further clustering by chemical similarity according to the Tanimoto distance by using the FCFP_4 (functional-class extended-connectivity fingerprint count up to diameter 4) fingerprint descriptors set available within Discovery Studio v2.1 suite (Accelrys Inc., San Diego, CA, USA).

Protein preparation, editing, atom typing, and structural alignment were performed using Accelrys Discovery Studio v2.1 (Accelrys Inc., San Diego, CA, USA). Co-crystallized ligands plus crystallographic waters found within 4 Å from the center of mass of the ligand structure were retained. Visual inspection and manual modification of the ligand structure valences were performed to ensure a proper state in the protein, and the 20 crystal structures of the selected human FXa-ligand complexes structures were aligned. After superimposition, CαRMSD against the lower resolution structure, was calculated. In addition, an all atom RMSD calculation, including only residues within 6Å from the center of mass of all crystallized ligands, was also performed. All FXa-ligand complexes atoms were typed with the CHARMM force field for further experiments [121].

### 3.2. Shape-Based Query Generation and Validation

The 3D alignment of the ligands was used to generate shape-based queries with vROCS v3.3.1.2 (OpenEye Scientific Software, Santa Fe, NM, USA) [122]. The queries were validated using the active compounds. The FXa inhibitors dataset from the extended database of useful decoys (DUD-E) database [74], which contains 792 actives and 24,017 decoy molecules, were also used. The in-house library was generated and standardized using InstantJChem v19.17.0 (ChemAxon Inc., Budapest, Hungary) and a multiple conformer database was generated by OMEGA v2.5.1.4 (OpenEye Scientific Software, Santa Fe, NM, USA) [123]. Shape-based screening was performed by using ROCS v3.3.1.2 using the Tanimoto Combo metric (OpenEye Scientific Software, Santa Fe, NM, USA) [72].

### 3.3. Synthesis of Derivatives

#### 3.3.1. Instrumentation and Chemicals

Sensitive reactions were carried out under a nitrogen atmosphere. Anhydrous solvents (tetrahydrofuran (THF), acetonitrile, and toluene) were dried over anhydrous calcium chloride and then stored over activated 0.3 nm molecular sieves. Other solvents (ethyl acetate, AcOEt; *n*-hexane, or others) were treated prior to their use by standard methods [124].

All starting materials were of the best available grade (Sigma-Aldrich, Merck, AK Scientific) and were used without further purification. Commercially available copper(II) chloride dihydrate was dehydrated upon heating in an oven (150 °C, 45 min) prior to use by the preparation of CuNPs. Column chromatography was performed with Merck silica gel 60 (0.040–0.063 mm, 240–400 mesh). Reactions were monitored by thin-layer chromatography on silica gel plates (60F-254) and visualized under UV.

Reactions were performed in a microwave Synthesis Reactor Monowave 200, Anton Paar. The melting points of synthesized derivatives were measured using an Electrothermal IA9100 digital melting point apparatus (Staffordshire, UK). Sonication was performed using a Hinotek SB-120DT Ultrasonic Cleaner ultrasound device. The nuclear magnetic resonance (NMR) spectra were recorded on Bruker Advance III HD 400 NMR spectrometer (400 MHz) with CDCl_3_ as a solvent, except where indicated. The NMR data were reported in δ (ppm) from tetramethylsilane (TMS). Multiplicities are abbreviated as follows: s = singlet, d = doublet, t = triplet, q = quartet, m = multiplet; brs = broad singlet. Coupling constants (J) were reported in Hz. The FT-IR spectra were recorded in a KBr pellet, on a Bruker VECTOR 22 spectrophotometer. High-resolution mass spectra (HRMS) were recorded on a Thermo Scientific Exactive Plus Orbitrap. Enzymatic activity was measured a Cytation^TM^ 5 Cell Imaging Multi-Mode Reader (Winooski, VT, USA) programmed to read continuously for 30 min at 37 °C, at a wavelength of Ex/Em 490 nm/520 nm.

#### 3.3.2. General Procedure for the Synthesis of Aniline Derivatives (**6**–**9**)

Microwave-assisted Ullmann-Goldberg coupling of 1.0 equivalent of the corresponding lactams (compound **1**–**4**) with 1.2 equiv. of 2-fluor-4-iodoaniline (**5**), 0.5 equiv. of CuI (except for compound **1** where 2 equivalents of catalyst were used), 2 equivalents of *N,N′*-dimethlyethylenediamine (DMEDA), and 2 equivalents of K_3_PO_4_ in 3.5 mL of dry toluene. The reaction mixture was irradiated in a microwave oven with 6–7 bar, 850 watts for 2 h at 160 °C (except for compound **7,** where 90 °C was used).

The resulting solution was passed through a pad of silica and Celite with ethyl acetate and the crude mixture was purified by flash column chromatography (silica gel, *n*-hexane/AcOEt, 5:5) [80].

#### 3.3.3. General Procedure for the Synthesis of N-propargyl Aniline Derivatives (**11**–**14**)

The synthesis of *N*-propargyl aniline derivatives was carried out using 1 equivalent of aniline (compounds **6**–**9**), 1.5 equiv. K_2_CO_3_ in 3 mL of dry acetonitrile. The mixture was stirred in a G-30 microwave instrument vessel at 500 rpm under an inert atmosphere of nitrogen for 20 min. In addition, in another vessel 0.1 equivalent of KI and 1 equivalent of propargyl bromide (**10**) solution 80% wt in toluene and 1 mL of acetonitrile were added. This mixture was added to the previous mixture reaction and irradiated with 7–8 bar, 850 watts at 160 °C for 30 min. The resulting solution was passed through a pad of silica and Celite with ethyl acetate and the crude mixture was purified by flash column chromatography (silica gel, *n*-hexane/AcOEt, 8:2) [80].

#### 3.3.4. General Procedure for the Synthesis of N-propargyl tetrahydroquinoline Derivatives (**17**–**20**)

1 equiv. of *N*-propargyl aniline derivative (compounds **11**–**14**), 1.1 equiv. of formaldehyde 30% in methanol (**15**) and 0.2 equiv. of InCl_3_ were mixed into a G-30 vessel. The reaction mixture was stirred at 500 rpm for 20 min under an inert atmosphere. Then, 1.1 equiv. of 1-vinyl-2-pyrrolidinone (**16**) were added to the mixture and irradiated with 7–8 bar, 850 watts of power at 160 °C for 15 min. The resulting solution was passed through a pad of silica and celite with ethyl acetate and the crude mixture was purified by flash column chromatography (silica gel, *n*-hexane/AcOEt, 9:1) [87,89].

*1-(8-Fluoro-4-(2-oxopyrrolidin-1-yl)-1-(prop-2-yn-1-yl)-1,2,3,4-tetrahydroquinolin-6-yl)piperidin-2-one* (**17**). Brown oil; 85% yield; FT-IR (KBr) ν / cm^−1^ 2924, 2098, 1743, 1681, 1604, 1489, 1381, 1134, 795; ^1^H NMR (400 MHz, CDCl_3_) δ 6.70 (dd, *J* = 14.2, 2.4 Hz, 1H), 6.49 (d, *J* = 2.4 Hz, 1H), 5.17 (dd, *J* = 9.8, 5.7 Hz, 1H), 3.92 (qd, *J* = 18.0, 2.4 Hz, 2H), 3.40 (ddt, *J* = 17.8, 11.5, 6.1 Hz, 2H), 3.20 (dtt, *J* = 17.2, 8.4, 4.1 Hz, 2H), 3.10 (dt, *J* = 9.8, 7.2 Hz, 2H), 3.01 (dt, *J* = 9.7, 6.6 Hz, 2H), 2.33 (d, *J* = 7.1 Hz, 2H), 2.30 (dd, *J* = 8.1, 3.6 Hz, 2H), 2.09 (t, *J* = 2.2 Hz, 1H), 1.84 (dt, *J* = 12.5, 6.3 Hz, 2H), 1.73 (dt, *J* = 6.4, 3.1 Hz, 4H); ^13^C NMR (101 MHz, CDCl_3_) δ 175.6, 169.9, 154.3, 151.8, 133.8, 126.8, 120.5, 113.1, 79.8, 72.5, 51.1, 48.1, 47.5, 43.1, 43.1, 32.3, 31.0, 24.3, 23.0, 20.8, 17.9; ^19^F NMR (376 MHz, CDCl_3_) *δ* −120.9; ESI-HRMS (*m/z):* calcd. for C_21_H_24_FN_3_O_2_ [M + H] ^+^: 369.1853, found 369.1847.

*4-(8-Fluoro-4-(2-oxopyrrolidin-1-yl)-1-(prop-2-yn-1-yl)-1,2,3,4-tetrahydroquinolin-6-yl)thiomorpholin-3-one* (**19**). Brown oil; 89% yield; FT-IR (KBr) ν / cm^−1^ 3533, 3224, 3055, 2854, 2106, 1705, 1573, 1504, 1118, 1064, 1987, 933, 856, 817, 786, 740, 632; ^1^H NMR (400 MHz, CDCl_3_) δ 6.81 (dd, *J* = 14.0, 2.8 Hz, 1H), 6.58 (d, *J* = 2.6 Hz, 1H), 5.28 (dd, *J* = 9.9, 5.0 Hz, 1H), 4.15–3.94 (m, 2H), 3.83 (tt, *J* = 5.2, 2.6 Hz, 2H), 3.34 (q, *J* = 1.9 Hz, 2H), 3.29 (td, *J* = 3.9, 2.1 Hz, 2H), 3.19 (qd, *J* = 7.2, 3.5 Hz, 2H), 3.11 (tt, *J* = 9.8, 5.1 Hz, 2H), 2.93 (dtd, *J* = 5.9, 4.2, 1.8 Hz, 2H), 2.42 (tq, *J* = 7.8, 4.9, 3.4 Hz, 2H), 2.15 (s, 1H), 1.95 (ddt, *J* = 9.7, 7.6, 2.8 Hz, 2H); ^13^C NMR (101 MHz, CDCl_3_) δ 175.7, 166.8, 154.5, 152.1, 134.0, 127.4, 120.7, 113.4, 80.0, 72.7, 52.1, 48.5, 47.8, 43.5, 43.3, 31.3, 30.5, 26.6, 24.7, 18.3; ^19^F NMR (376 MHz, CDCl_3_) δ −120.3; ESI-HRMS (*m/z):* calcd. for C_20_H_22_FN_3_O_2_S [M + H]^+^: 387.1417, found 388.1410.

*4-(8-Fluoro-4-(2-oxopyrrolidin-1-yl)-1-(prop-2-yn-1-yl)-1,2,3,4-tetrahydroquinolin-6-yl)morpholin-3-one* (**20**)**.** Brown oil; 83% yield; FT-IR (KBr) ν / cm^−1^ 3317, 3240, 3070, 2939, 2885, 2368, 2106, 1697, 1573, 1504, 1111, 1056, 995, 956, 925, 864, 786, 725, 648; ^1^H NMR (400 MHz, CDCl_3_) δ 6.91 (dd, *J* = 14.2, 2.5 Hz, 1H), 6.69 (dt, *J* = 2.5, 1.2 Hz, 1H), 5.32 (dd, *J* = 9.9, 5.7 Hz, 1H), 4.23 (s, 2H), 4.18–3.95 (m, 2H), 3.94 (td, *J* = 4.8, 4.2, 1.2 Hz, 2H), 3.71–3.55 (m, 2H), 3.42–3.27 (m, 2H), 3.18 (ddt, *J* = 37.6, 9.7, 7.0 Hz, 2H), 2.44 (td, *J* = 7.9, 2.4 Hz, 2H), 2.16 (s, 1H), 2.15 (qd, *J* = 10.8, 10.3, 5.2 Hz, 2H), 1.97 (q, *J* = 7.6 Hz, 2H); ^13^C NMR (101 MHz, CDCl_3_) δ 175.8, 166.6, 154.6, 152.1, 133.3, 127.4, 120.0, 112.8, 80.0, 72.8, 68.5, 64.0, 49.4, 48.6, 47.9, 43.5, 43.4, 31.3, 24.8, 18.4; ^19^F NMR (376 MHz, CDCl_3_) δ −120.2; ESI-HRMS (*m/z):* calcd. for C_20_H_22_FN_3_O_3_ [M + H]^+^: 371.1645, found 371.1644.

#### 3.3.5. General Procedure for the Synthesis of Azides (**24**–**26**)

A 100-mL round bottom flask was charged with 4 equiv. of aniline (compounds **21–23**), water (4 mL) as a solvent, and 2.6 equiv. of concentrated HCl were added to the vigorously stirred reaction mixture. Then, 4.2 equiv. of NaNO_2_ in water (6.0 mL) were added dropwise into the reaction mixture. Next, the mixture was sonicated at 40 °C for an additional 10 min. Then, a freshly prepared solution of 4.2 equiv. of NaN_3_ in water (12.0 mL) was added dropwise into the reaction mixture. Upon complete addition of the sodium azide solution, the reaction mixture was stirred for 1 h. The reaction mixture was extracted with EtOAc (3 × 10 mL) and washed with water (10 mL). The organic layer was dried with MgSO_4_, then filtered and the solvent removed in vacuo to afford the corresponding arylazide a quantitative yield.

#### 3.3.6. General Procedure for the synthesis of 1*H*-1,2,3-triazole Derivatives (**27**–**38**)

In a G-30 vessel, 1 equiv. of *N*-propargyl amines (compounds **17–20**), 1 equiv. of aryl azide (compounds **24**–**26**), 0.85 mol% of copper nanoparticles supported on ZnO (CuNPs/ZnO, synthesis detailed in the next section) as a catalyst, 1 equiv. of Et_3_N in THF were added. The reaction mixture was heated at 160 °C under microwave irradiation for 15 min with a pressure of 7.7 bar at 850 W. The corresponding triazoles were purified by flash column chromatography (silica gel, *n*-hexane/AcOEt, 3:7).

##### Synthesis of Copper Nanoparticles Supported on ZnO

The catalyst used in the synthesis of different triazoles were prepared according to the procedure reported in the literature [125].

Anhydrous copper(II) chloride (135 mg, 1 mmol) was added to a suspension of lithium (14 mg, 2 mmol) and 4,4′-di-tert-butylbiphenyl (DTBB, 27 mg, 0.1 mmol) in THF (2 mL) at room temperature under a nitrogen atmosphere. The reaction mixture, which was initially dark blue, rapidly changed to black, indicating that the suspension of copper nanoparticles was formed. This suspension was diluted with THF (8 mL) followed by the addition of the zinc oxide (800 mg). The resulting mixture was stirred for 1 h at room temperature, filtered, and the solid successively washed with ethanol (20 mL) and then dried under vacuum.

*1-(3-Fluoro-4-(((1-phenyl-1H-1,2,3-triazol-4-yl)methyl)amino)phenyl)piperidin-2-one* (**27**)**.** Brown solid; 92% yield; m.p. 146–148 °C; FT-IR, λmáx (KBr), ν / cm^−1^ 3418, 3132, 2855, 1651, 1528, 1466, 1420, 1342, 1273, 1227, 1173, 1119, 1049, 926, 856, 826, 756, 687; ^1^H NMR (400 MHz, DMSO) δ 8.68 (s, 1H), 8.00–7.81 (m, 2H), 7.63–7.51 (m, 2H), 7.50–7.42 (m, 1H), 6.99 (dd, *J* = 13.0, 2.2 Hz, 1H), 6.90–6.72 (m, 2H), 6.04 (td, *J* = 6.0, 2.1 Hz, 1H), 4.46 (d, *J* = 5.9 Hz, 2H), 3.49 (t, *J* = 5.6 Hz, 2H), 2.32 (t, *J* = 6.2 Hz, 2H), 1.79 (qt, *J* = 8.4, 4.2 Hz, 4H); ^13^C NMR (101 MHz, DMSO) δ 168.7, 151.3, 148.9, 146.7, 136.7, 133.4, 129.8 (2C), 128.5, 122.3, 121.1, 119.9 (2C), 113.2, 111.6, 51.1, 38.3, 32.5, 23.0, 20.9; ^19^F NMR (376 MHz, DMSO) δ −133.5; **ESI-HRMS** (*m/z):* calcd for C_20_H_20_FN_5_O [M + H]^+^: 365,1652; found 366,1713.

*1-(4-(((1-(4-Chlorophenyl)-1H-1,2,3-triazol-4-yl)methyl)amino)-3-fluorophenyl) piperidin-2-one* (**28**)**.** Brown solid; 90% yield; m.p. 180–181 °C; FT-IR, λmáx (KBr), ν / cm^−1^ 3402, 2955, 1643, 1520, 1412, 1273, 1227, 1173, 1111, 1088, 1042, 988, 926, 826; ^1^H NMR (400 MHz, CDCl_3_) δ 7.89 (s, 1H), 7.73–7.61 (m, 2H), 7.52–7.39 (m, 2H), 6.94 (d, *J* = 2.3 Hz, 1H), 6.85 (dd, *J* = 8.6, 2.3 Hz, 1H), 6.72 (t, *J* = 9.0 Hz, 1H), 4.57 (s, 1H), 4.56 (s, 2H), 3.55 (h, *J* = 3.7 Hz, 2H), 2.57–2.45 (m, 2H), 1.90 (p, *J* = 3.3 Hz, 4H); ^13^C NMR (101 MHz, CDCl_3_) δ 170.4, 152.4, 150.0, 147.2, 135.6, 134.8, 134.6, 133.3, 130.0, 122.5, 121.8, 120.0, 113.7, 113.5, 112.3, 52.1, 39.7, 32.9, 23.6, 21.5; ^19^F NMR (376 MHz, CDCl_3_) δ −133.9; ESI-HRMS (*m/z):* calcd for C_20_H_19_ClFN_5_O [M + H]^+^: 399,1262; found 400.1334.

*1-(3-Fluoro-4-(((1-(4-fluorophenyl)-1H-1,2,3-triazol-4-yl)methyl)amino)phenyl) piperidin-2-one***(29).** Brown solid; 76% yield; m.p. 139–140 °C; **FT-IR,** λmáx (KBr), ν / cm^−1^ 3318, 3140, 3071, 2947, 2862, 1643, 1520, 1412, 1335, 1273, 1227, 1180, 1119, 1049, 980, 934, 841, 625; ^1^H NMR (400 MHz, CDCl_3_) δ 7.88 (s, 1H), 7.65 (ddt, *J* = 6.6, 4.5, 2.5 Hz, 2H), 7.14 (t, *J* = 8.6 Hz, 2H), 6.89 (dd, *J* = 12.3, 2.4 Hz, 1H), 6.82 (dd, *J* = 8.6, 2.4 Hz, 1H), 6.69 (t, *J* = 8.8 Hz, 1H), 4.61 (q, *J* = 6.1 Hz, 1H), 4.50 (d, *J* = 5.6 Hz, 2H), 3.52 (t, *J* = 5.5 Hz, 2H), 2.48 (t, *J* = 5.8 Hz, 2H), 1.87 (p, *J* = 3.4 Hz, 4H); ^13^C NMR (101 MHz, CDCl_3_) δ 170.3, 163.6, 161.1, 152.2, 149.8, 146.9, 134.8, 133.2, 122.5, 122.3, 120.3, 116.6, 113.4, 112.1, 52.0, 39.5, 32.8, 29.7, 23.5, 21.4; ^19^F NMR (376 MHz, CDCl_3_) δ −112.3, −133.9; ESI-HRMS (*m/z):* calcd for C_20_H_19_F_2_N_5_O [M + H]^+^: 383,1558; found 384,1771.

*Tert-butyl 4-(3-fluoro-4-(((1-phenyl-1H-1,2,3-triazol-4-yl)methyl)amino)phenyl)-3-oxopiperazine-1-carboxylate***(30).** Brown solid; 90% yield; m.p. 90–92 °C; FT-IR, λmáx (KBr), ν / cm^−1^ 3348, 2924, 2855, 1528, 1420, 1327, 1242, 1042; ^1^H NMR (400 MHz, CDCl_3_) δ 7.91 (s, 1H), 7.69 (dd, *J* = 7.8, 1.6 Hz, 2H), 7.48 (t, *J* = 7.8 Hz, 2H), 7.46–7.36 (m, 1H), 6.95 (dd, *J* = 12.1, 2.4 Hz, 1H), 6.94–6.80 (m, 1H), 6.75 (t, *J* = 8.9 Hz, 1H), 4.65 (d, *J* = 15.1 Hz, 1H), 4.56 (s, 2H), 4.20 (s, 2H), 3.73 (t, *J* = 5.3 Hz, 2H), 3.63 (dd, *J* = 6.2, 4.4 Hz, 2H), 1.47 (s, 9H); ^13^C NMR (101 MHz, CDCl_3_) δ 166.0, 153.9, 152.2, 149.8, 146.6, 137.0, 135.3, 131.4, 129.8, 128.9, 122.0, 120.6, 120.0, 113.1, 112.3, 81.0, 50.2, 48.3, 40.4, 39.6, 29.8, 28.4 (3C); ^19^F NMR (376 MHz, CDCl_3_) δ −133.5; ESI-HRMS (*m/z):* calcd for C_24_H_27_FN_6_O_3_ [M + H]^+^: 466,2129; found 466,2097.

*Tert-butyl 4-(4-(((1-(4-chlorophenyl)-1H-1,2,3-triazol-4-yl)methyl)amino)-3-fluorophenyl)-3-oxopiperazine-1-carboxylate***(31).** Brown solid; 80% yield; m.p. 129–130 °C; FT-IR, λmáx (KBr), ν / cm^−1^ 3433, 3140, 3109, 2978, 2847, 1697, 1535, 1404, 1335, 1234, 1173, 1126, 1088, 1049, 1018, 988, 941, 864, 833, 795, 764, 733, 656; ^1^H NMR (400 MHz, CDCl_3_) δ 7.89 (s, 1H), 7.71–7.60 (m, 2H), 7.52–7.41 (m, 2H), 6.96 (dd, *J* = 12.1, 2.3 Hz, 1H), 6.87 (ddd, *J* = 8.6, 2.4, 1.1 Hz, 1H), 6.73 (t, *J* = 9.0 Hz, 1H), 4.62 (s, 1H), 4.56 (d, *J* = 5.1 Hz, 2H), 4.20 (s, 2H), 3.74 (dd, *J* = 6.4, 4.3 Hz, 2H), 3.63 (dd, *J* = 6.3, 4.3 Hz, 2H), 1.48 (s, 9H); ^13^C NMR (101 MHz, CDCl_3_) δ 165.9, 153.8, 152.1, 149.8, 146.9, 135.5, 135.2, 134.6, 131.4, 129.9, 122.0, 121.7, 119.9, 113.2, 113.0, 112.2, 81.0, 50.2, 48.2, 40.4, 39.5, 28.4 (3C); ^19^F NMR (376 MHz, CDCl_3_) δ −133.5; **ESI-HRMS** (*m/z):* calcd for C_24_H_26_ClFN_6_O_3_ [M + H]^+^: 500,1739; found 501,1817.

*Tert-butyl 4-(3-fluoro-4-(((1-(4-fluorophenyl)-1H-1,2,3-triazol-4-yl)methyl)amino)phenyl)-3-oxopiperazine-1-carboxylate***(32).** Brown solid; 73% yield; m.p. 80–82 °C; FT-IR, λmáx (KBr), ν / cm^−1^ 3588, 3341, 3140, 3078, 2978, 2924, 2862, 2778. 2361, 1705, 1528, 1481, 1412, 1366, 1327, 1288, 1234, 1173, 1126, 1042, 1018, 988, 941, 841, 756, 656; ^1^H NMR (400 MHz, CDCl_3_) δ 7.88 (s, 1H), 7.68–7.59 (m, 2H), 7.19–7.08 (m, 2H), 6.92 (dd, *J* = 12.1, 2.2 Hz, 1H), 6.83 (ddd, *J* = 8.6, 2.4, 1.1 Hz, 1H), 6.70 (t, *J* = 9.0 Hz, 1H), 4.68 (td, *J* = 5.9, 2.7 Hz, 1H), 4.50 (d, *J* = 5.8 Hz, 2H), 4.17 (s, 2H), 3.71 (dd, *J* = 6.4, 4.2 Hz, 2H), 3.60 (dd, *J* = 6.2, 4.4 Hz, 2H), 1.45 (s, 9H); ^13^C NMR (101 MHz, CDCl_3_) δ 165.9, 163.6, 161.1, 153.8, 152.1, 149.7, 146.7, 135.2, 133.2, 131.2, 122.4, 121.9, 120.2, 116.6, 113.0, 112.1, 80.9, 50.1, 48.2, 40.6, 39.3, 28.3 (3C); ^19^F NMR (376 MHz, CDCl_3_) δ −112.2, −133.5; ESI-HRMS (*m/z):* calcd for C_24_H_26_F_2_N_6_O_3_ [M − H]^+^: 484,2034, found 483,1983.

*4-(3-Fluoro-4-(((1-phenyl-1H-1,2,3-triazol-4-yl)methyl)amino)phenyl)thiomorpholine-3-one***(33).** Brown solid; 93% yield; m.p. 178–180 °C; FT-IR, λmáx (KBr), ν / cm^−1^ 3140, 2924, 1659, 1528, 1466, 1427, 1327, 1273, 1219, 1111; ^1^H NMR (400 MHz, CDCl_3_) δ 7.91 (s, 1H), 7.70 (dd, *J* = 7.5, 1.6 Hz, 2H), 7.49 (t, *J* = 7.7 Hz, 2H), 7.41 (t, *J* = 7.4 Hz, 1H), 6.94 (dd, *J* = 12.0, 2.3 Hz, 1H), 6.86 (dd, *J* = 8.5, 2.4 Hz, 1H), 6.75 (t, *J* = 8.9 Hz, 1H), 4.59 (s, 1H), 4.56 (d, *J* = 4.7 Hz, 2H), 3.92–3.84 (m, 2H), 3.41 (s, 2H), 2.98 (dd, *J* = 6.8, 4.7 Hz, 2H); ^13^C NMR (101 MHz, CDCl_3_) δ 167.1, 152.2, 149.8, 146.6, 137.1, 135.3, 132.4, 129.8, 128.9, 122.4, 120.6, 120.0, 113.5, 112.3, 52.8, 39.6, 30.7, 29.8, 26.8; ^19^F NMR (376 MHz, CDCl_3_) δ −133.6; ESI-HRMS (*m/z):* calcd for C_19_H_18_FN_5_OS [M + H]^+^: 383,1216; found 384,1278.

*4-(4-(((1-(4-Chlorophenyl)-1H-1,2,3-triazol-4-yl)methyl)amino)-3-fluorophenyl) thiomorpholine-3-one***(34).** Brown solid; 88% yield; m.p. 240–242 °C; FT-IR, λmáx (KBr), ν / cm^−1^ 3426, 3132, 3063, 1659, 1589, 1528, 1504, 1404, 1342, 1273, 1234, 1188, 1126, 1088, 1042, 988, 957, 895, 864, 826, 718, 687, 648, 617; ^1^H NMR (400 MHz, DMSO) δ 8.71 (s, 1H), 7.93 (d, *J* = 8.9 Hz, 2H), 7.64 (d, *J* = 8.9 Hz, 2H), 7.01 (dd, *J* = 12.8, 2.3 Hz, 1H), 6.87 (dd, *J* = 8.7, 2.3 Hz, 1H), 6.79 (t, *J* = 9.1 Hz, 1H), 6.12 (td, *J* = 6.1, 2.1 Hz, 1H), 4.47 (d, *J* = 5.9 Hz, 2H), 3.85 (t, *J* = 5.8 Hz, 2H), 3.34 (d, *J* = 11.2 Hz, 2H), 2.99 (t, *J* = 5.8 Hz, 2H); ^13^C NMR (101 MHz, DMSO) δ 166.9, 151.2, 148.8, 146.9, 135.4, 134.7, 132.8, 131.4, 129.8, 122.2, 122.2, 121.6, 121.2, 113.0, 111.6, 51.4, 38.2, 29.5, 26.1; ^19^F NMR (376 MHz, DMSO) δ −133.3; ESI-HRMS (*m/z):* calcd for C_19_H_17_ClFN_5_OS [M + H]^+^: 417,0826; found 418,0906.

*4-(3-Fluoro-4-(((1-(4-fluorophenyl)-1H-1,2,3-triazol-4-yl)methyl)amino)phenyl) thiomorpholine-3-one***(35).** Brown solid; 75% yield; m.p. 161–163 °C; FT-IR, λmáx (KBr), ν / cm^−1^ 3356, 3240, 1651, 1528, 1420, 1319, 1281, 1242, 1211, 1188, 1126, 1111, 1026, 964, 910, 864, 818, 772, 702, 648; ^1^H NMR (400 MHz, CDCl_3_) δ 7.88 (s, 1H), 7.69–7.61 (m, 2H), 7.20–7.11 (m, 2H), 6.90 (dd, *J* = 12.1, 2.3 Hz, 1H), 6.83 (dt, *J* = 8.5, 1.6 Hz, 1H), 6.70 (t, *J* = 8.9 Hz, 1H), 4.64 (td, *J* = 6.0, 2.7 Hz, 1H), 4.51 (d, *J* = 5.8 Hz, 2H), 3.89–3.81 (m, 2H), 3.38 (s, 2H), 2.97–2.94 (m, 2H); ^13^C NMR (101 MHz, CDCl_3_) δ 167.1, 163.6, 161.1, 152.1, 149.7, 146.7, 135.1, 133.2, 132.3, 122.5, 122.3, 120.2, 116.7, 113.4, 112.1, 52.7, 39.4, 30.6, 26.7; ^19^F NMR (376 MHz, CDCl_3_) δ −112.1, −133.6; ESI-HRMS (*m/z):* calcd for C_19_H_17_F_2_N_5_OS [M + H]^+^: 401,1122; found 401,1901.

*4-(3-Fluoro-4-(((1-phenyl-1H-1,2,3-triazol-4-yl)methyl)amino)phenyl)morpholin-3-one***(36).** Brown solid; 90% yield; m.p. 168–169 °C; FT-IR, λmáx (KBr), ν / cm^−1^ 3426, 1651, 1535, 1474, 1420, 1342, 1234, 1119; ^1^H NMR (400 MHz, CDCl_3_) δ 7.91 (s, 1H), 7.68 (dd, *J* = 7.7, 1.8 Hz, 2H), 7.51–7.43 (m, 2H), 7.40 (dd, *J* = 8.5, 6.4 Hz, 1H), 6.99 (dd, *J* = 12.2, 2.4 Hz, 1H), 6.90 (dd, *J* = 8.8, 2.4 Hz, 1H), 6.74 (t, *J* = 9.0 Hz, 1H), 4.66 (d, *J* = 7.1 Hz, 1H), 4.54 (d, *J* = 4.5 Hz, 2H), 4.27 (s, 2H), 3.96 (dd, *J* = 6.1, 4.1 Hz, 2H), 3.65 (dd, *J* = 6.0, 4.1 Hz, 2H); ^13^C NMR (101 MHz, CDCl_3_) δ 166.9, 152.2, 149.8, 146.6, 137.0, 135.2, 130.9, 129.8, 128.8, 121.8, 120.5, 120.0, 113.0, 112.8, 112.2, 68.6, 64.1, 50.1, 39.5; ^19^F NMR (376 MHz, CDCl_3_) δ −133.5; ESI-HRMS (*m/z):* calcd for C_19_H_18_FN_5_O_2_ [M − H]^+^: 367,1445; found 366,1372.

*4-(4-(((1-(4-Chlorophenyl)-1H-1,2,3-triazol-4-yl)methyl)amino)-3-fluorophenyl) morpholin-3-one***(37).** Brown solid; 86% yield; m.p. 235–237 °C; FT-IR, λmáx (KBr), ν / cm^−1^ 3372, 3102, 2862, 1667, 1620, 1520, 1466, 1427, 1319, 1281, 1219, 1180, 1111, 1049, 980, 895, 864, 671, 625; ^1^H NMR (400 MHz, DMSO) δ 8.71 (s, 1H), 8.04–7.86 (m, 2H), 7.73–7.60 (m, 2H), 7.13 (dd, *J* = 13.0, 2.3 Hz, 1H), 6.99–6.91 (m, 1H), 6.81 (dd, *J* = 9.8, 8.7 Hz, 1H), 6.14 (td, *J* = 6.0, 2.1 Hz, 1H), 4.47 (d, *J* = 5.9 Hz, 2H), 4.14 (s, 2H), 3.97–3.88 (m, 2H), 3.69–3.59 (m, 2H); ^13^C NMR (101 MHz, DMSO) δ 165.9, 151.2, 148.8, 146.9, 135.5, 134.8, 132.8, 130.2, 129.8, 121.8, 121.6, 121.2, 112.6, 111.6, 67.7, 63.5, 54.9, 49.3, 38.2; ^19^F NMR (376 MHz, DMSO) δ −133.2; ESI-HRMS (*m/z):* calcd for C_19_H_17_ClFN_5_O_2_ [M + H]^+^: 401,1055; found 402,1119.

*4-(3-Fluoro-4-(((1-(4-fluorophenyl)-1H-1,2,3-triazol-4-yl)methyl)amino)phenyl) morpholin-3-one***(38).** Brown solid; 78% yield; m.p. 153–154 °C; FT-IR, λmáx (KBr), ν / cm^−1^ 3364, 3125, 3094, 2862, 1667, 1620, 1520, 1466, 1427, 1319, 1281, 1227, 1180, 1157, 1111, 1049, 980, 941, 895, 856, 818, 671, 625; ^1^H NMR (400 MHz, DMSO) δ 8.67 (s, 1H), 7.93 (dd, *J* = 9.0, 4.7 Hz, 2H), 7.42 (t, *J* = 8.8 Hz, 2H), 7.13 (dd, *J* = 12.9, 1.9 Hz, 1H), 6.96 (dd, *J* = 8.7, 2.5 Hz, 1H), 6.81 (t, *J* = 9.2 Hz, 1H), 6.13 (td, *J* = 5.9, 2.1 Hz, 1H), 4.47 (d, *J* = 6.0 Hz, 2H), 4.14 (s, 2H), 3.92 (t, *J* = 5.1 Hz, 2H), 3.62 (t, *J* = 5.0 Hz, 2H); ^13^C NMR (101 MHz, DMSO) δ 166.4, 163.2, 160.8, 151.7, 149.3, 147.2, 135.3, 133.7, 130.7, 122.8, 122.3, 121.8, 117.1, 113.1, 112.1, 68.2, 64.0, 49.78, 38.7. ^19^F NMR (376 MHz, DMSO) δ −113.4, −133.2; ESI-HRMS (*m/z):* calcd for C_19_H_17_F_2_N_5_O_2_ [M + H]^+^: 385,1350; found 386,1413.

### 3.4. Theoretical Study

The Gaussian 09 computational package was used. It was used to perform ground-state geometry optimization and reactivity parameters calculations, without symmetry constraints, employing Becke’s three-parameter hybrid functional combined with Lee-Yang-Parr’s nonlocal correlation functional B3LYP. A 6-311G basis set was employed [126].

The FMOs GAP, reactivity indexes and Fukui functions were calculated as follows [97,127]:

(1) $$\mathrm{GAP}=\varepsilon_{LUMO}-\;\varepsilon_{HOMO}$$

(2) $$\mathrm{Chemical}\;\mathrm{Hardness}\;\eta =\frac{1}{2}\left(\varepsilon_{LUMO}-\;\varepsilon_{HOMO}\right)$$

(3) $$\mathrm{Chemical}\;\mathrm{Potential}\;\mu =\frac{1}{2}\left(\varepsilon_{LUMO}+\;\varepsilon_{HOMO}\right)$$

(4) $$\mathrm{Electrophilicity}\;\omega =\frac{2\mu }{2\eta }$$

(5) $$f^{+}=\left|q_{N+1}-q_{N}\right|$$

(6) $$f^{-}=\left|q_{N}-q_{N-1}\right|$$

(7) $$f^{0}=\left|\frac{1}{2}(q_{N+1}-q_{N-1})\right|$$

### 3.5. Biological Analysis

#### 3.5.1. FXa in Vitro Inhibition Assay

All compounds were evaluated in vitro for their FXa enzyme inhibitory activity using as the positive control Rivaroxaban (gold standard inhibitor) and Gabexate mesylate (FXa kit inhibitor). First, all the proposed compounds were screened at 100 µM to obtain a preliminary result of the potential inhibitory activity of the novel molecules. The enzymatic assay was prepared according to the manufacturer’s instructions (Sensolyte^®^ Rh 110 factor Xa Assay Kit *Fluorometric*, Anaspec, Fremont, CA, USA). Inhibition control 1 mM was diluted 1:10 with the prepared buffer 1x ibtaining of the 0.1 mM inhibition control. The reagents containing compound dilutions, buffers, and enzymes were mixed, centrifuged, and incubated for 30 min at 37 °C in 96-well microtiter plates. The enzyme reaction was initiated by adding an appropriate substrate (FXa, S-2222). The 96-well plate for fluorescence was prepared with 40 μL of enzyme and 1 µL of each compound. To initiate the enzymatic reaction 50 µL of the FXa substrate was added to each well. In the second trial, the selected compounds (**27**, **30**, **31,** and **34**) were prepared to span five different concentrations: 0.1–100 µM. All inhibitory assays were performed three times in triplicate. For compounds with FXa inhibition over 50%, respectively, IC_50_ values were calculated from the mean of triplicates from a dilution series of the compound with GraphPad Prism v. 8.0.2 (GraphPad Inc., MA, USA).

#### 3.5.2. Coagulation Parameters Analyses: ex Vivo Assay

All measurements were read on the ACL TOP 500 CTS spectrophotometer equipment (Instrumental Laboratory, Milano, Italy), as it can perform medium to high volume recesses. To measure, monitor, and analyze Prothrombin time (PT), activated partial thromboplastin time (aPTT), anti-FXa activity were used. All methodologies were used with their corresponding calibrators and controls. Statistical analysis was expressed as a mean ± SD. Statistical comparison was performed by using the Student *t* test. Two-sided *p* < 0.05 was considered statistically significant.

##### Anti-FXa Assay

The anti-FXa assay was performed by using the Hemosil Liquid anti-FXa kit (IL^®^LAX, Instrumentation Laboratory, Orangeburg, NY, USA) and the measures were done through ACL Top 500 CTS spectrophotometer instrument (Instrumental Laboratory, Italy). The assay was prepared according to the manufacturer’s instructions. The solution anti-Xa kit is a one-stage chromogenic assay based on a synthetic chromogenic substrate and an FXa inactivation. FXa is neutralized by the tested compounds. Residual FXa is quantified with a synthetic chromogenic substrate. The *p*-nitroaniline released is monitored kinetically at 405 nm and is inversely proportional to the compounds in the sample (**27**, **30**, **31**, **34**, Apixaban, Rivaroxaban). The anti-FXa activity measurement was expressed as heparin activity (IU/mL). Anti-FXa analysis was performed in triplicate.

##### Prothrombin Time (PT) Test

It is the most important clinical screening test in the evaluation of disorders of the extrinsic coagulation pathway. A highly sensitivity thromboplastin reagent, based on recombinant human tissue factor (RTF), was done for the quantitative determination in human citrated plasma of PT. Pathway and domino activation reactions are the target of this assay, which is widely used in hemostasis field (RecombiPlasTin 2G-0020003050, HemosIL^®^, Milano, Italy). The assay was prepared according to the manufacturer’s instructions. PT analysis was performed in triplicate.

##### Activated Partial Thromboplastin Time (aPTT) Test

A contact activator was used to stimulate the production of Factor XIIa by providing a surface for the function of high molecular weight kininogen, kallikrein, and Factor XIIa. This contact activation proceeds at 37 °C for a couple of minutes. Calcium is then added to trigger further reactions and the time required for clot formation is measured. Phospholipids are necessary to form complexes that activate Factor X. Assay provides a highly reproducible and stable response. Prolonged clotting times are observed in the presence of heparin, or another inhibitor.

aPTT results may be reported in seconds in plasma pool assays; it is not affected by hemoglobin up to 100 mg/dL or triglycerides up to 1200 mg/dL. The final step was to remove the tube and tilt once per second and stop the stopwatch when the first vestige of clot formation (APTT-SP (liquid)-0020006300, HemosIL^®^) was noticed. aPTT analysis was performed in triplicate.

#### 3.5.3. Cell Viability Assays

The human cell lines (HEK293 and HepG2) used in the cell viability assays were purchased from American Type Culture Collection (Manassas, VA, USA). HEK293 cell lines were cultured in Dulbecco’s modified Eagle medium supplemented with 10% fetal bovine serum, 100 units/mL penicillin and 100 mg/mL streptomycin and kept at 37 °C in a humidified atmosphere containing 5% CO_2_. A 100-μL aliquot of adherent cells was used to seed 96-well cell culture plates at 30,000 cells/well and allowed to adhere for 16 h prior to the addition of the compounds.

HepG2 cells were maintained in Dulbecco’s Modified Eagle Medium (DMEM-F12) with 5% fetal bovine serum (FBS), and penicillin/streptomycin 1%, under standard cell culture conditions (37 °C, humidified, 5% CO_2_). Cells were plated at a density of 25,000 cells/well in a 96-well plate and incubated in a growth medium for 16 h. Cells were treated with each compound in DMEM containing 10% FBS for 48 h. Subsequently, 20 μL MTS reagent was added into each well and incubated for 4 h. The cell viability was detected by the CellTiter 96 AQueous One solution Cell Proliferation Assay kit (Promega Corporation, Madison, WI, USA). All experiments were done in sextuplicate.

### 3.6. Computational Analysis

#### 3.6.1. Docking Studies

FXa consists of 254 amino acid catalytic domains and the substrate cleaves at its active site in FFNPR-TF and YIDGR-IV in prothrombin to form thrombin. Basically, FXa has four binding pockets that are involved in the substrate-specific cleavage making them an important target for ligands designed for inhibition activity. These active sites host different amino residues, which play a vital role in ligand binding through hydrogen bonding. The four binding pockets present in the active site are S1, S2, S3, and S4. The synthesized compounds found to interact with the S1 residues make them potential inhibitors of FXa. S1 pocket is symmetrical, and *U* shaped, whereas this site in thrombin is less symmetrical and shallower due to the presence of L99 in place of Y99 in FXa [128].

FXa S1 pocket is hydrophobic in nature and contains Y228, D189, and S195 residues. Y228 of S1 pockets plays a vital role in forming the important hydrogen bonds with the ligands. S4 is another hydrophobic site present on FXa and has residues like F174, Y99, and W215. These residues form the aromatic box capable of being bound to ligands having aliphatic, aromatic, and positively charged components. Because of its affinity towards the positively charged moieties, it is also known as a cation hole. S4 site of FXa makes its distinct form from the thrombin in terms of ligand specificity. This is because S4 site of FXa is perfectly symmetrical when compared to thrombin and another site of differentiation in the presence of Y99 and F174 residues in FXa and L99 and I174 amino residue in thrombin. Among the residues of site S4, residue Y99 is the most crucial for selectivity towards FXa [128]. Similarly, W215 formed the basis of the U shape pocket and played a vital role in binding with the synthesized compounds. Residue C191, which has the disulfide linkage with C220, is present at the bottom of the S1 site, is highly conserved, and hence, was expected to play an important role in FXa inhibition.

Basically, the S4 site of FXa is aromatic in nature due to the presence of aromatic residues and mostly host hydrophobic and positively charged substrates. On the other hand, S4 site of thrombin primarily hosts the substrates like aliphatic and aromatic functionalities because of the presence of the aliphatic and aromatic residues such as I174, L99, N98, E97, and W215. The difference between the affinity towards the different functionalities plays a key role in designing the selective FXa or thrombin inhibitor. Two other pharmacologically insignificant sites are S2 and S3. S1 site is small, shallow, and is a host Y99 residue, whereas the S3 site is flat and present at the edge of S1. Both sites are not as significant as that of S1 and S4 [128].

In the present work, which was aimed to design the specific factor Xa inhibitors, all computational calculations were performed using the Schrödinger’s Small-Molecule Drug Discovery Suite [129]. The FXa protein was prepared by adding hydrogens, assigning bond order and generating rotamers and protonation states using Protein Preparation Wizard of Schrödinger. All compounds were drawn with ChemDraw and prepared using the software LigPrep, while ionization/tautomeric states were predicted using Epik [130].

Finally, after H bond optimization, the protein was subjected to a molecular energy minimization using the Impref module of impact. The FXa crystal structure complexed with the Apixaban (PDB code 2P16) was used for the docking experiments.

The docking calculations using rigid-receptors and flexible-ligands were performed with Glide through the Single Precision (SP) mode [112]. A Docking grid box was centered on the apixaban co-crystallized to define the center of the grid box. Optimization of the resulting enzyme-ligand complexes was conducted using Prime [131,132]. The docking poses for each ligand were analyzed by examining the Emodel score and their interaction with residues at the binding sites. Three more energetically favorable conformations were selected as the best poses. The Molecular Mechanics/Generalized Born Surface Area (MM/GBSA) approach was used as implemented in the Prime module from Schrödinger Suites using the default settings. Protein-ligand complexes were obtained from docking experiments, as described above.

#### 3.6.2. Molecular Dynamics Simulations

Molecular dynamics (MD) simulations were performed using Desmond Software [116]. The OPLS3e force-field was used to model the amino acid interactions in the protein, and the TIP3P was used as a water model [133,134]. Equilibration of the system was carried out using the protocol provided in Desmond. The initial coordinates for the MD simulation were taken from the protein-ligand complex. The TIP3P water model was added (the orthorhombic dimensions of each water box were 10 Å × 10 Å × 10 Å approximately), and the system was neutralized by adding Cl^−^ counterions to balance the net charge of the system. The whole system was subjected to 300 K for 100 ns of simulation, with NPT ensemble. The structural changes and dynamic behavior of the protein were analyzed by calculating the RMSD.

#### 3.6.3. In Silico Pharmacokinetics Prediction

To better understand the overall properties of the synthesized compounds, the drugs with properties using QikProp [119], which calculate drug score as a sum of different physical and chemical properties such as drug-likeness and toxicity risks, were examined. It was remarkable that all the described propargyl-THQ derivatives possess logP values (expressed as the octanol/ water partition coefficient) compatible with those required to cross membranes. An important physicochemical parameter for drug discovery is the aqueous solubility calculated as logS. All compounds evaluated, presented LogS values into the recommended range. LogS parameter is relevant because it reflects the bioavailability of the compound, especially in oral drugs because the poor solubility limits the absorption of the compound by the gastrointestinal tract [135,136]. Moreover, none of the compounds violated Lipinski’s rule (molecular weight, log P, number of hydrogen donors and acceptors) showing that all compounds could be used as a safe oral drug [137,138]. As FXa inhibitors have to pass different membranes and reach the CNS, the evaluated properties as logBB (Predicted brain/blood partition coefficient) and PHOA (Predicted human oral absorption) support the potential of these derivatives as drug candidates. Molecular descriptors were determined using SwissADME platforms by using the Boiled-egg graph (Supplementary Material, S26) as it shows classifiers for important ADME behaviors. Passive gastro-intestinal (HIA) absorption and blood-brain barrier (BBB) permeation were predicted with the BOILED-Egg mode, for the egg white and yolk, respectively [118].

## 4. Conclusions

By using a three-step improved microwave methodology, a novel FXa inhibitor compounds **27** (1-(3-fluoro-4-(((1-phenyl-1H-1,2,3-triazol-4-yl)methyl)amino)phenyl)piperidin-2-one) and **34** (4-(4-(((1-(4-chlorophenyl)-1H-1,2,3-triazol-4-yl)methyl)amino)-3-fluorophenyl) thiomorpholine-3-one) has been successfully designed and synthesized. Moreover, theoretical studies enabled us to understand the reactivity, enzyme interactions and pharmacokinetics properties of all novel synthesized molecules. These novel compounds have been exhibited in vitro FXa inhibition activity in micromolar concentrations. Moreover, compound **27** prolonged aPTT and PT in a dose-dependent manner suggesting it can be used in both intrinsic and extrinsic clotting pathways. Furthermore, compound **34** only prolonged PT in a dose-dependent manner and it may target coagulation FXa by the extrinsic and common pathway. Taking into account the in silico pharmacokinetics results, compounds **27** and **34** showed logP < 5, appropriate logBB, logS and PHOA values that encourage us to utilize these novel molecules as interesting scaffolds for potential anticoagulant oral administration drugs.

## Figures and Tables

**Table 1:** Summary of crystal structures of Factor Xa-ligand complexes used in this study.

PDB ID (Reference)	Resolution (Å)	RMSD Cluster Center (Å)	^1^ RMSD Binding Site (6Å)	^2^ Tanimoto Distance
4BTI [70]	2.30	-----	-----	0.000
4BTU [70]	2.37	0.440	0.222	0.426
4BTT [70]	2.59	0.530	0.381	0.582
2W26 Rivaroxaban [34]	2.08	0.276	0.207	0.618
2P16 Apixaban [71]	2.30	0.290	0.381	0.689

^1^ All atoms within 6 Å from the center of mass of ligand alignment. ^2^ Tanimoto coefficient.

**Table 2:** Optimization of reaction conditions for the synthesis of compound **6**. ^1,2.^

Entry	CuI (equiv.)	DMEDA (equiv.)	T (°C)	Time (h)	Heating Source	Yield (%)
1	0.05	0.1	20	96	r.t.	-
2	0.05	0.1	100	72	conventional	-
3	0.1	0.2	100	72	conventional	1
4	0.05	0.5	100	72	conventional	-
5	0.05	0.1	120	48	conventional	1
6	0.1	0.2	120	48	conventional	4
7	0.05	0.5	120	48	conventional	8
8	0.05	0.1	60	6	sonication	-
9	0.1	0.2	60	6	sonication	5
10	0.05	0.5	60	6	sonication	20
11	0.05	0.1	120	2	microwave	6
12	0.1	0.2	120	2	microwave	8
13	0.15	0.3	120	2	microwave	11
14	0.25	0.5	120	2	microwave	17
15	0.5	0.5	120	2	microwave	19
16	1.00	1.0	120	2	microwave	26
17	2.00	2.0	120	2	microwave	46
18	2.00	2.0	160	1.5	microwave	50
19^3^	2.00	2.0	160	1.5	microwave	62

^1^ Conditions: lactam (1.2 equiv.), aniline (1 equiv.), K_3_PO_4_ (2 equiv.), toluene (3.5 mL). ^2^ Yield of isolated compound. ^3^ Reaction carried out using 1 equiv. of lactam and 1.2 equiv. of aniline. r.t.: room temperature.

**Table 3:** Optimization of reaction conditions for the synthesis of compounds **11**–**14**.^1,2.^

Entry	Heating Source	T (°C)	Time (h)	Yield (%)			
				11	12	13	14
1	r.t.	20	96	13	19	10	9
2	conventional	80	24	31	40	29	15
3	conventional	80	72	36	47	34	30
4	conventional	90	24	37	49	37	34
5	sonication	40	15	43	57	45	39
6	sonication	80	4	55	67	51	43
7	microwave	160	0.5	**69**	**72**	**70**	**67**

^1^ Conditions: aniline derivatives (1.2 equiv.), propargyl bromide (1 equiv.), K_2_CO_3_ (1.5 equiv.), KI (0.1 equiv.), in acetonitrile (3.5 mL). ^2^ Yield of isolated compounds. r.t.: room temperature

**Table 4:** Optimization of reaction conditions for the synthesis of compounds **17**–**20**.^1,2.^

Entry	Heating Source	T (°C)	Time (h)	Yield (%)			
				17	18	19	20
1	r.t.	20	96	32	-	34	36
2	conventional	80	24	47	-	45	52
3	conventional	80	72	53	-	54	59
4	conventional	90	24	59	-	56	62
5	sonication	40	8	69	-	64	67
6	sonication	80	2	72	-	72	73
7	microwave	160	0.5	**85**	-	**89**	**83**

^1^ Conditions: *N*-propargyl aniline derivatives (1 equiv.), formaldehyde 30% in methanol (1.1 equiv.), InCl_3_ (0.2 equiv.), KI (0.1 equiv.), in acetonitrile (3 mL). ^2^ Yield of isolated compounds.

**Table 5:** Selected bond lengths and angles for molecule **6**.

Bond Length	CALC	EXP	Crystal Structure ^1^
F(1)-C(10)	1.366	1.339	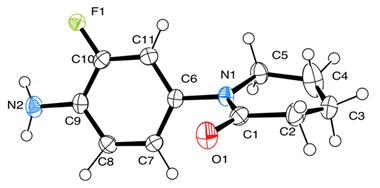
O(1)-C(1)	1.220	1.232	
N(1)-C(1)	1.382	1.354	
N(1)-C(6)	1.435	1.438	
N(1)-C(5)	1.477	1.480	
N(2)-C(9)	1.391	1.382	
C(2)-C(3)	1.529	1.540	
C(3)-C(4)	1.301	1.497	
**Bond Angles**	**CALC**	**EXP**	
C(4)-C(3)-C(2)	109.2	109.4	
C(3)-C(2)-C(1)	115.9	115.24	
C(5)-N(1)-C(6)	116.3	115.67	
C(1)-N(1)-C(6)	118.8	119.09	
C(11)-C(10)-F(1)	119.2	119.26	
C(11)-C(6)-N(1)-C(1)	112.7	116.25	
C(4)-C(5)-C(1)-C(2)	14.8	9.1	

^1^ ORTEP [95] drawing with thermal ellipsoids drawn at the 50% probability level. ORTEP: Oak Ridge Thermal Ellipsoid Plot.

**Table 6:** Selected bond lengths and angles for molecule **9**.

Bond Length	CALC	EXP	Crystal Structure ^1^
F(1)-C(7)	1.365	1.364	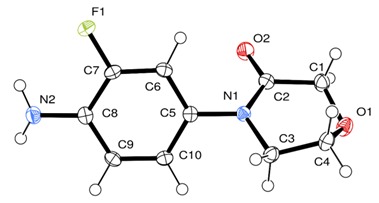
O(2)-C(2)	1.219	1.231	
N(1)-C(2)	1.377	1.352	
N(1)-C(5)	1.433	1.436	
N(1)-C(3)	1.474	1.479	
N(2)-C(8)	1.390	1.383	
C(1)-O(1)	1.418	1.418	
O(1)-C(4)	1.417	1.430	
**Bond Angles**	**CALC**	**EXP**	
C(4)-O(1)-C(1)	110.6	109.4	
O(1)-C(1)-C(2)	116.7	115.6	
C(3)-N(1)-C(5)	117.6	117.2	
C(2)-N(1)-C(5)	120.8	120.7	
C(6)-C(7)-F(1)	119.0	118.3	
C(6)-C(5)-N(1)-C(2)	50.0	50.2	
C(4)-C(3)-C(2)-C(1)	18.7	16.0	
C(10)-C(5)-N(1)-C(2)	50.2	131.7	

^1^ ORTEP [95] drawing with thermal ellipsoids drawn at the 50% probability level.

**Table 7:** Selected bond lengths and angles for molecule **20**.

Bond Length	CALC	EXP	Crystal Structure ^1^
F(1)-C(4)	1.365	1.363	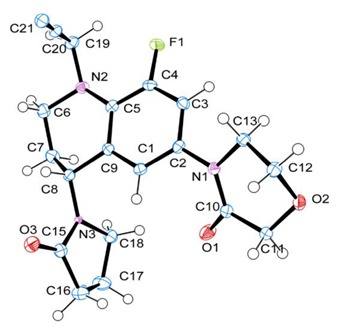
O(1)-C(10)	1.219	1.222	
N(1)-C(2)	1.432	1.436	
N(1)-C(10)	1.378	1.368	
N(1)-C(13)	1.474	1.475	
N(2)-C(5)	1.393	1.411	
N(2)-C(6)	1.465	1.474	
N(3)-C(8)	1.474	1.457	
N(3)-C(18)	1.464	1.471	
N(3)-C(15)	1.372	1.356	
O(3)-C(15)	1.219	1.231	
O(2)-C(12)	1.417	1.426	
O(2)-C(11)	1.418	1.421	
C(8)-N(3)	1.474	1.457	
**Bond Angles**	**CALC**	**EXP**	
C(12)-O(2)-C(11)	110.7	108.3	
O(2)-C(11)-C(10)	116.5	115.1	
C(13)-N(1)-C(2)	117.6	117.4	
C(10)-N(1)-C(2)	120.5	120.1	
C(3)-C(4)-F(1)	116.5	115.8	
C(5)-N(2)-C(19)	122.1	117.8	
C(3)-C(2)-N(1)-C(10)	126.8	137.7	
C(12)-C(13)-C(10)-C(11)	18.2	13.1	
C(5)-N(2)-C(19)-C(20)	128.5	55.3	
C(9)-C(8)-N(3)-C(15)	153.8	111.2	
C(21)-N(2)-C(8)-N(3)	108.7	158.0	

^1^ ORTEP [95] drawing with thermal ellipsoids drawn at a 50% probability level.

**Table 8:** Calculated energies (eV) for FMOs and reactivity indexes, for precursors and final compounds.

Compound	HOMO	LUMO	GAP	HARD. (η)	C. POT. (μ)	ELEC. (ω)
**11**	−5.3917	−0.3859	5.0058	2.5029	−2.8888	1.6671
**12**	−5.5550	−0.5519	5.0031	2.5016	−3.0534	1.8635
**13**	−5.8366	−0.5010	5.3357	2.6678	−3.1688	1.8819
**14**	−5.5593	−0.5954	4.9639	2.4820	−3.0774	1.9078
**27**	−5.4418	−1.5467	3.8951	1.9475	−3.4942	3.1347
**30**	−5.5892	−1.5913	3.9979	1.9989	−3.5903	3.2242
**33**	−5.8339	−1.6205	4.2134	2.1067	−3.7272	3.2970
**36**	−5.5982	−1.6003	3.9979	1.9990	−3.5993	3.2404
**28**	−5.5354	−1.8583	3.6771	1.8386	−3.6968	3.7167
**31**	−5.6674	−1.8931	3.7742	1.8871	−3.7802	3.7862
**34**	−5.9278	−1.9249	4.0028	2.0014	−3.9264	3.8514
**37**	−5.6973	−1.9111	3.7862	1.8931	−3.8042	3.8222
**29**	−5.5147	−1.7138	3.8009	1.9005	−3.6142	3.4367
**32**	−5.6524	−1.7557	3.8967	1.9484	−3.7040	3.5209
**35**	−5.9082	−1.7843	4.1239	2.0620	−3.8462	3.5872
**38**	−5.6750	−1.7682	3.9068	1.9534	−3.7216	3.5452
**24**	−7.1049	−1.4487	5.6562	2.8281	−4.2768	3.2339
**25**	−7.2350	−1.6602	5.5748	2.7874	−4.4476	3.5483
**26**	–7.2467	–1.7339	5.5128	2.7564	–4.4903	3.6575

**Table 9:** FXa inhibition and IC_50_ values of novel compounds **6**–**38**.

Compound^a^	Inhibition FXa (%)^b^	IC_50_ ± SD [μM]^c^
**6**	19	-
**7**	18	-
**8**	20	-
**10**	15	-
**11**	17	-
**12**	14	-
**13**	25	-
**14**	24	-
**17**	25	-
**19**	27	-
**20**	15	-
**27**	52	102.10 ± 0.14
**28**	33	-
**29**	46	-
**30**	63	41.71 ± 0.07
**31**	73	29.73 ± 0.09
**32**	21	-
**33**	41	-
**34**	60	67.92 ± 0.08
**35**	28	-
**36**	34	-
**37**	34	-
**38**	39	-

^a^ It was not possible to synthesize Compound 18. ^b^ Inhibition percentage at 100 μM. ^c^ Apixaban 97% inhibition, IC_50_ = 2.8 nM. Rivaroxaban 94% inhibition, IC_50_ = 0.70 nM.

**Table 10:** Anti FXa assay values of selected compound.

Compound	Anti-FXa [IU/mL] ^a^
**27**	0.29 ± 0.10
**30**	0.00
**31**	0.05 ± 0.09
**34**	0.42 ± 0.18

^a^ IU/mL: International Units per milliliter. Concentration 1 mM. Apixaban (500 nM, 1.50 ± 0.36 IU/mL) and Rivaroxaban (300 nM, 1.20 ± 0.23 IU/mL), respectively.

**Table 11:** Predicted Glide SP score values obtained from molecular docking for selected compounds **8, 19, 27, 30, 31,** and **34.**

Compound	Glide Emodel (kcal/mol)	Glide EVdW (Kcal/mol)	∆G_bind_ (Kcal/mol)	∆G_bind VdW_ (Kcal/mol)
**8**	−56.62	−36.62	−53.15	−40.49
**19**	−45.66	−27.25	−49.49	−26.66
**27**	−79.77	−44.64	−67.39	−49.17
**30**	−77.10	−49.62	−52.84	−46.57
**31**	−82.94	−54.62	−65.81	−58.48
**34**	−82.89	−51.19	−64.28	−53.24

Emodel is a specific combination of Docking Score, CVdW (CVdW = Coul + VdW is the non-bonded interaction energy between the ligand and the receptor) and the internal torsional energy of the ligand conformer. ∆G_bind VdW_ is the VdW interaction energy between ligand and receptor calculated using Glide SP. ∆G_bind_ and ∆G_bind VdW_ were obtained through a MM-GBSA approach implemented in the Prime module of the Schrödinger suite. CVdW: Coulomb-van der Waals interaction-energy score. MM-GBSA: Molecular Mechanics-Generalized Born Surface Area.

**Table 12:** Drug likeness score of synthesized compounds.

Compound	logPo/w	Molecular Weight	logS	H-Bonds Donors	H-Bonds Acceptors	logBB	PHOA
**7**	2.30	309.34	−3.48	1.5	6.5	0.80	88.62
**8**	1.71	226.27	−2.60	1.5	4.5	0.12	91.48
**9**	0.76	210.21	−2.02	1.5	5.7	0.21	85.49
**11**	2.81	246.28	−3.91	1.5	4.0	0.10	100.00
**12**	3.58	347.39	−5.82	1.5	6.5	0.57	100.00
**13**	2.83	264.32	−4.01	1.5	4.5	0.08	100.00
**14**	1.94	248.26	−3.00	1.5	5.7	0.04	100.00
**17**	2.83	369.44	−3.92	0.5	7.0	0.22	96.68
**18**	3.37	470.54	−5.80	0.5	9.5	0.85	93.68
**19**	2.75	387.47	−3.99	0.5	7.5	0.10	96.52
**20**	1.79	371.41	−2.90	0.5	8.7	0.25	89.95
**27**	4.02	365.41	−6.25	1.0	6.0	0.63	100.00
**28**	4.51	399.85	−6.99	1.0	6.0	−0.48	100.00
**29**	4.25	383.40	−6.62	1.0	6.0	−0.53	100.00
**30**	4.51	466.51	−7.63	1.0	8.5	−1.26	100.00
**31**	4.99	500.96	−8.35	1.0	8.5	−1.11	90.07
**33**	3.94	383.44	−6.17	1.0	6.5	−0.51	100.00
**34**	4.74	484.50	−7.99	1.0	8.5	−1.15	100.00
**34**	4.44	417.89	−6.91	1.0	6.5	−0.35	100.00
**35**	4.18	401.43	−6.54	1.0	6.5	−0.40	100.00
**36**	2.97	367.38	−5.01	1.0	7.7	−0.63	100.00
**37**	3.46	401.83	−5.75	1.0	7.7	−0.48	100.00
**38**	3.21	385.37	−5.43	1.0	7.7	−0.54	100.00

QPlogPo/w = Predicted octanol / water partition coefficient. Recommended values −2.0–6.5. donor HB = Estimated number of hydrogen bonds that would be donated in an aqueous solution. Values are averages taken over a number of configurations, thus, they can be non-integer. Acceptors HB = Estimated number of hydrogen bonds that would be accepted in an aqueous solution. Values are averages taken over a number of configurations, thus, they can be non-integer. QPlogS = Predicted aqueous solubility, log S. Recommended values −6.5 to 0.5. QPlogBB = Predicted brain/blood partition coefficient. Recommended values −3.0 to 1.2. logBB = Passive blood-brain partitioning values expressed in terms of logarithm. PHOA= Predicted human oral absorption on 0 to 100% scale. Recommended values >80% is high <25% is poor.

## Supplementary Materials

The following are available online at https://www.mdpi.com/1420-3049/25/3/491/s1, NMR (^1^H, ^13^C, ^19^F), HRMS, FT-IR for all novel synthesized compounds; FT-IR stacking for **11**–**14**, **17**–**20**, **27**–**38**; ROCs AUC curve, boiled-egg diagram, crystal data and structure refinement for molecules **6**, **9**, and **20**; Fukui functions for molecules 14 and 25, and calculated thermochemical energies data.
