# Supplementary material for: Innovative Three-Step Microwave-Promoted Synthesis of N-Propargyltetrahydroquinoline and 1,2,3-Triazole Derivatives as a Potential Factor Xa (FXa) Inhibitors: Drug Design, Synthesis, and Biological Evaluation
Source: Molecules. 2020 Jan 23;25(3):491. doi: 10.3390/molecules25030491 (PMC7037264; doi:10.3390/molecules25030491)
Supplement: Supplementary file 1 [file molecules-25-00491-s001.pdf]

SUPPLEMENTARY MATERIAL

Innovative three-step microwave-promoted synthesis of *N*-propargyltetrahydroquinoline and 1,2,3-triazole derivatives as a potential factor Xa (FXa) inhibitors: drug design, synthesis, and biological evaluation.

Comentado [FZ1]: The title was changed

TABLE OF CONTENTS

^{1}H, ^{13}C, ^{19}F NMR; ESI-HRMS; FT-IR for compound 17	S1
^{1}H, ^{13}C, ^{19}F NMR; ESI-HRMS; FT-IR for compound 19	S2
^{1}H, ^{13}C, ^{19}F NMR; ESI-HRMS; FT-IR for compound 20	S3
^{1}H, ^{13}C, ^{19}F NMR; ESI-HRMS; FT-IR for compound 27	S4
^{1}H, ^{13}C, ^{19}F NMR; ESI-HRMS; FT-IR for compound 28	S5
^{1}H, ^{13}C, ^{19}F NMR; ESI-HRMS; FT-IR for compound 29	S6
^{1}H, ^{13}C, ^{19}F NMR; ESI-HRMS; FT-IR for compound 30	S7
^{1}H, ^{13}C, ^{19}F NMR; ESI-HRMS; FT-IR for compound 31	S8
^{1}H, ^{13}C, ^{19}F NMR; ESI-HRMS; FT-IR for compound 32	S9
^{1}H, ^{13}C, ^{19}F NMR; ESI-HRMS; FT-IR for compound 33	S10
^{1}H, ^{13}C, ^{19}F NMR; ESI-HRMS; FT-IR for compound 34	S11
^{1}H, ^{13}C, ^{19}F NMR; ESI-HRMS; FT-IR for compound 35	S12
^{1}H, ^{13}C, ^{19}F NMR; ESI-HRMS; FT-IR for compound 36	S13
^{1}H, ^{13}C, ^{19}F NMR; ESI-HRMS; FT-IR for compound 37	S14
^{1}H, ^{13}C, ^{19}F NMR; ESI-HRMS; FT-IR for compound 38	S15
FT-IR stacking for aniline compounds (6-9)	S16
FT-IR stacking for *N*-propargyl aniline compounds (11-14)	S17
FT-IR stacking for *N*-propargyl tetrahydroquinoline compounds (17-20)	S18
FT-IR stacking for ^{1}*H*-1,2,3-triazole compounds (27,30,33,36)	S19
FT-IR stacking for ^{1}*H*-1,2,3-triazole compounds (28,31,34,37)	S20
FT-IR stacking for ^{1}*H*-1,2,3-triazole compounds (29,32,35,38)	S21
Table of Calculated log Pa of synthesized compounds (cont.)	S22
Reaction optimization for the synthesis of compounds 7-9	S23
Dynamic RMSD for compound 19	S24
ROCs AUC curve for method enrichment	S25
Boiled egg diagram for blood brain-barrier	S26
Crystal data and structure refinement for compound 6	S27
Crystal data and structure refinement for compound 9	S28
Crystal data and structure refinement for compound 20	S29
Dihedral bond angle difference between calculated and experimental compound 9	S30
Hydrogen bond formed in compound 9 crystal	S31
Interactions between the propargyl and carbonyl group in the crystal structure of compound 20	S32
Fukui functions for compound 14	S33
Fukui functions for compound 25	S34
Reaction species according to Fukui calculations	S35
Calculated thermochemical energies	S36

S1. ^{1}H, ^{13}C, ^{19}F NMR; ESI-HRMS; FT-IR for compound 17

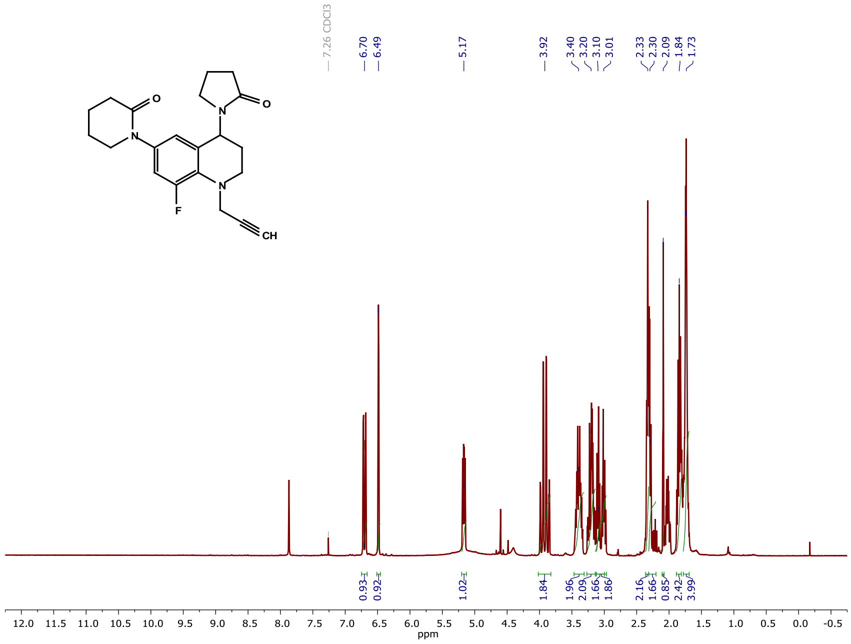

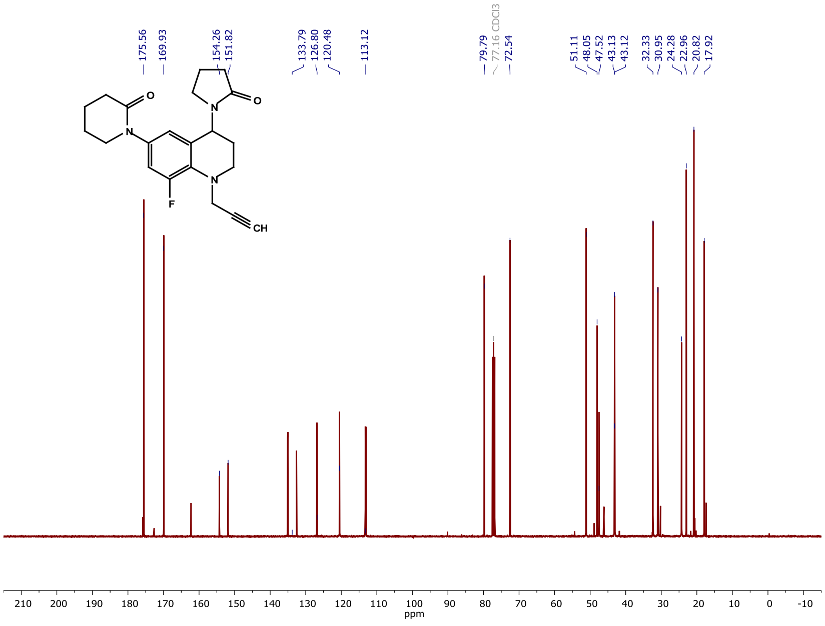

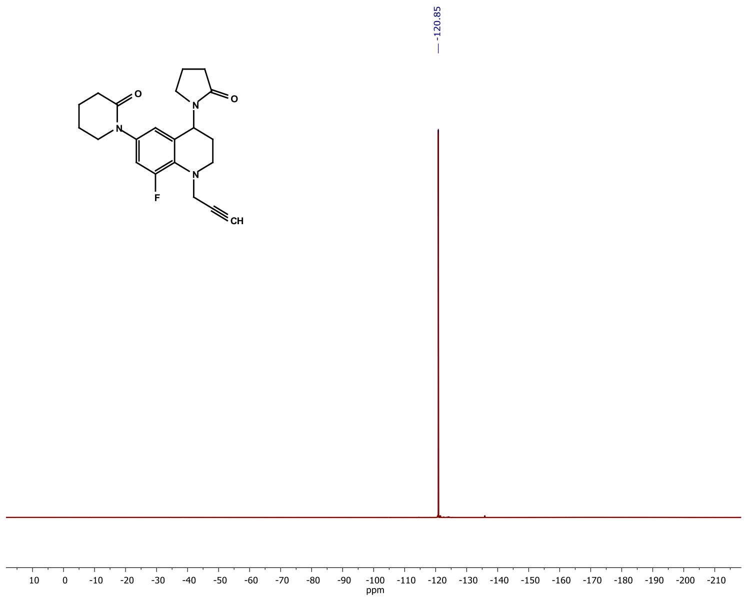

-120.85
O
O
N
N
N
F
CH
10
0
-10
-20
-30
-40
-50
-60
-70
-80
-90
-100
-110
-120
-130
-140
-150
-160
-170
-180
-190
-200
-210
ppm

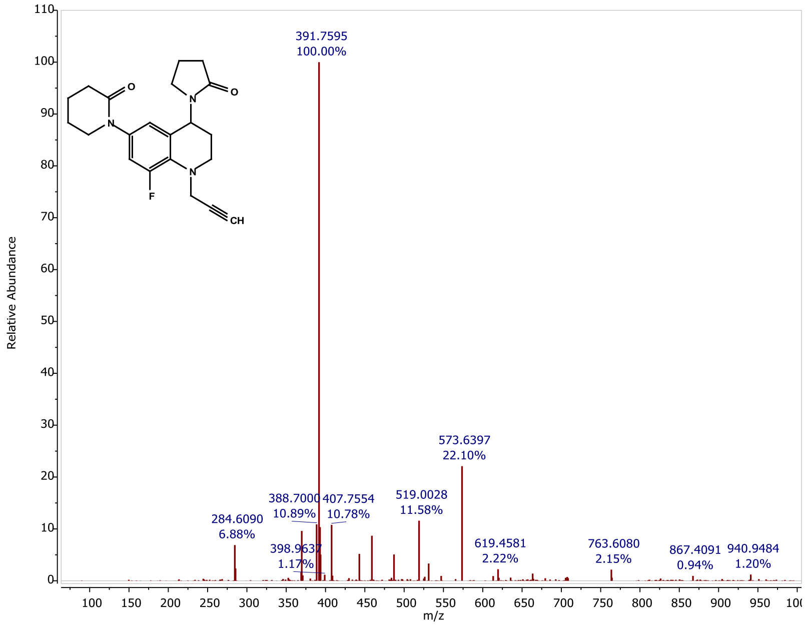

Relative Abundance
m/z
391.7595
100.00%
573.6397
22.10%
388.7000
10.89%
407.7554
10.78%
519.0028
11.58%
284.6090
6.88%
398.9637
1.17%
619.4581
2.22%
763.6080
2.15%
867.4091
0.94%
940.9484
1.20%
O
O
N
N
N
F
CH

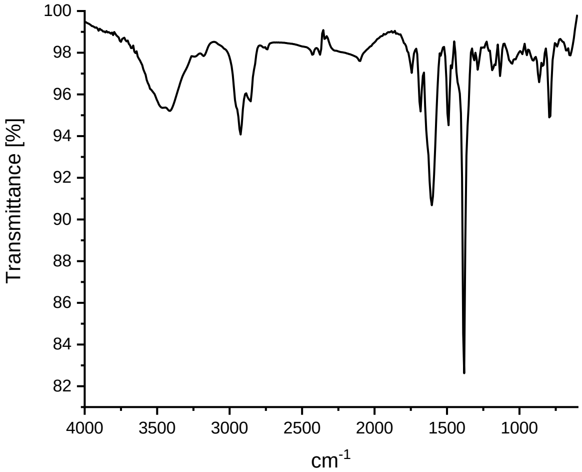

S2. ^{1}H, ^{13}C, ^{19}F NMR; ESI-HRMS; FT-IR for compound 19

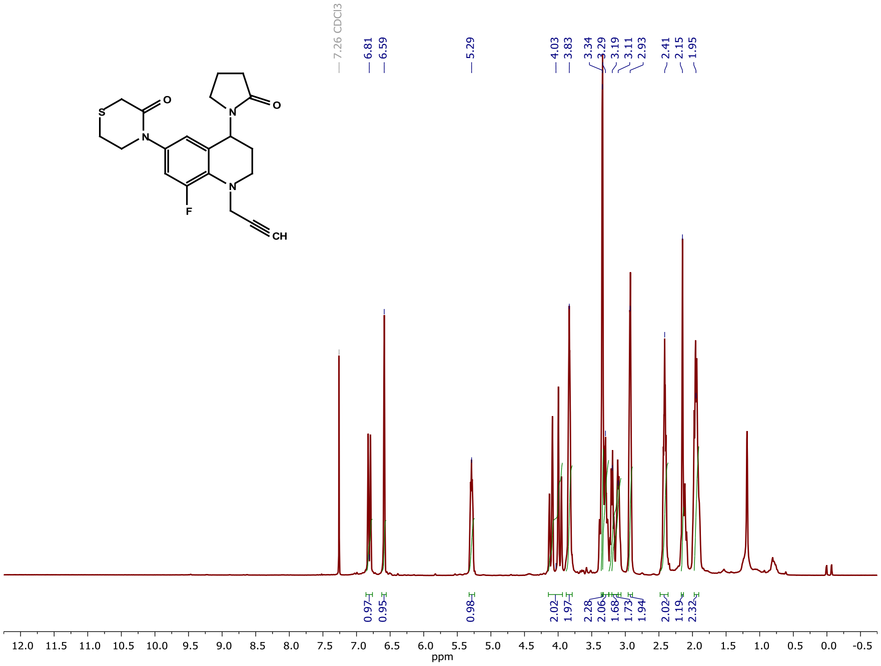

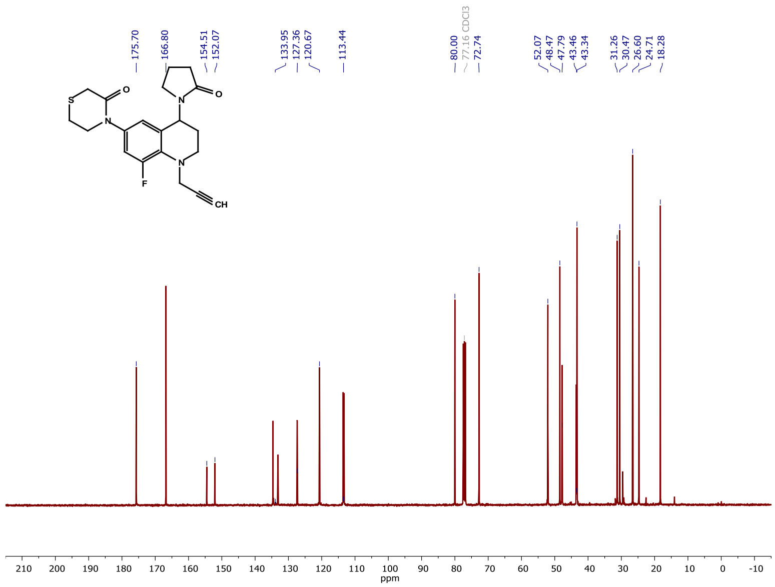
175.70
166.80
154.51
152.07
133.95
127.36
120.67
113.44
80.00
77.16 CDCl3
72.74
52.07
48.47
47.79
43.46
43.34
31.26
30.47
26.60
24.71
18.28
S
O
N
N
O
N
F
CH
210 200 190 180 170 160 150 140 130 120 110 100 90 80 70 60 50 40 30 20 10 0 -10
ppm

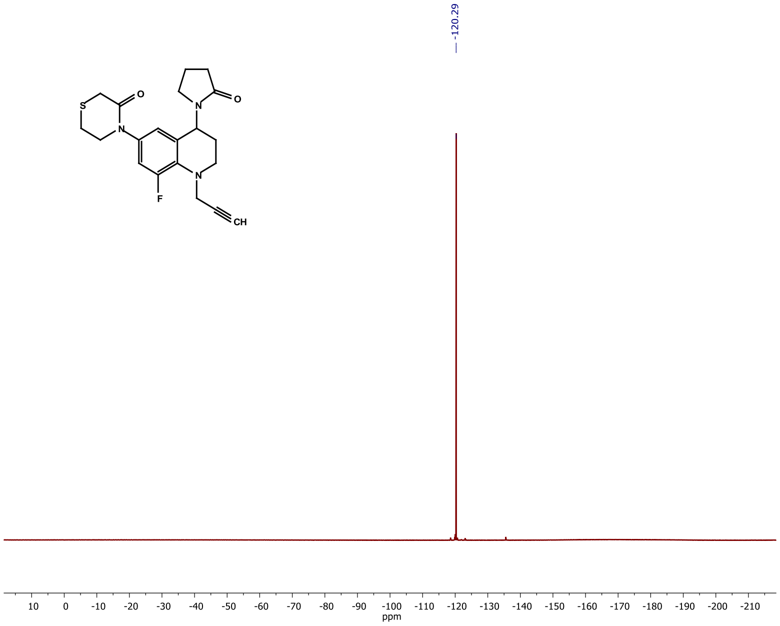
-120.29
S
O
N
N
O
N
F
CH
10 0 -10 -20 -30 -40 -50 -60 -70 -80 -90 -100 -110 -120 -130 -140 -150 -160 -170 -180 -190 -200 -210
ppm

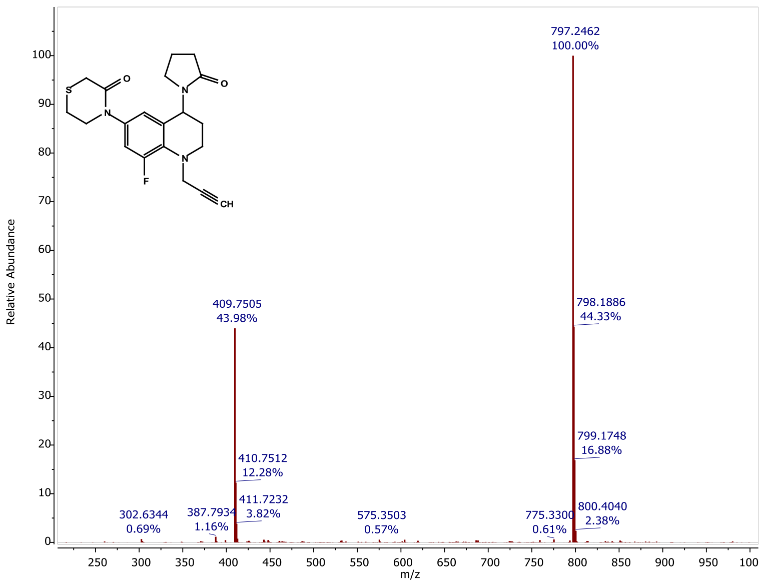
Relative Abundance
m/z
797.2462
100.00%
409.7505
43.98%
798.1886
44.33%
799.1748
16.88%
410.7512
12.28%
411.7232
3.82%
302.6344
0.69%
387.7934
1.16%
575.3503
0.57%
775.3300
0.61%
800.4040
2.38%

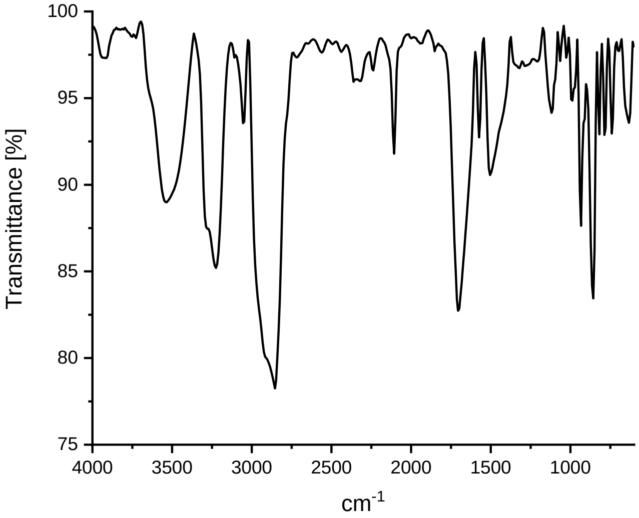
Transmittance [%]
cm-1

S3. ¹H, ¹³C, ¹⁹F NMR; ESI-HRMS; FT-IR for compound 20

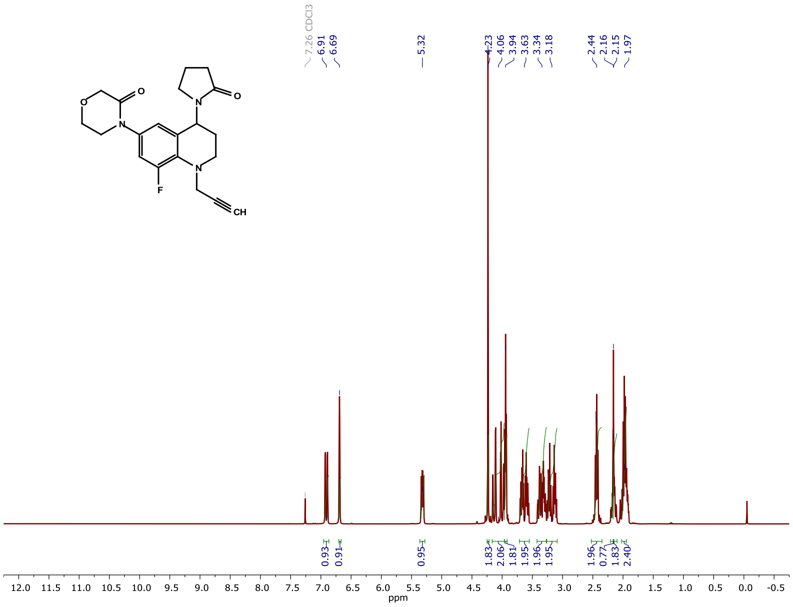

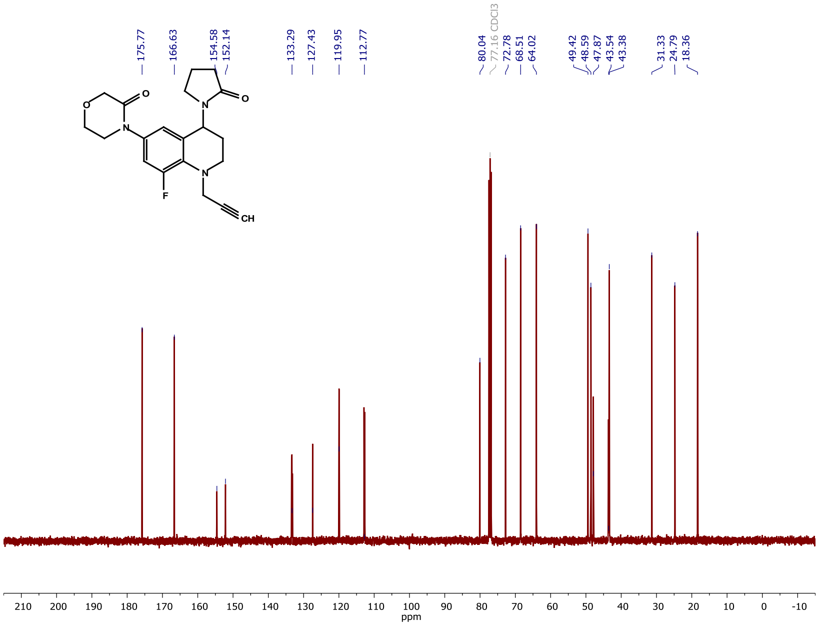

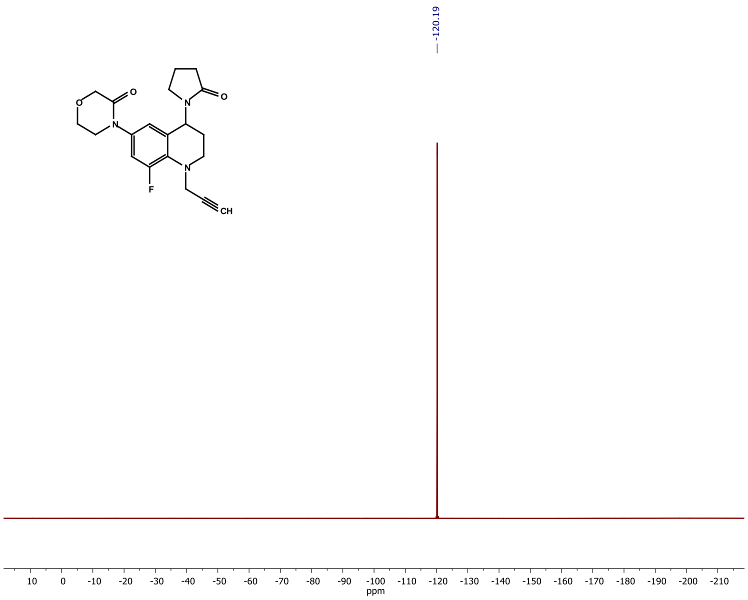

-120.19
O
N
O
N
O
N
F
CH
ppm

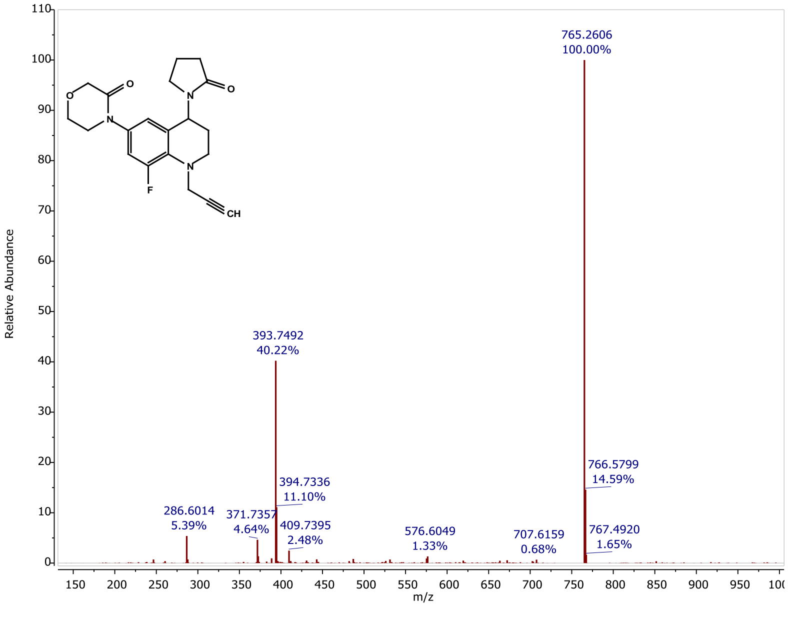

765.2606
100.00%
393.7492
40.22%
766.5799
14.59%
394.7336
11.10%
286.6014
5.39%
371.7357
4.64%
409.7395
2.48%
576.6049
1.33%
707.6159
0.68%
767.4920
1.65%
Relative Abundance
m/z

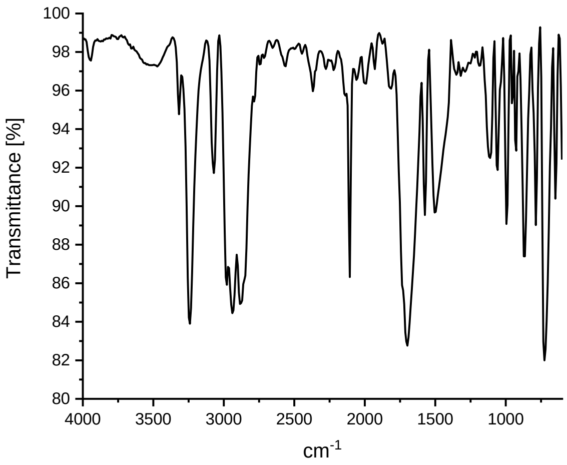

S4. ^{1}H, ^{13}C, ^{19}F NMR; ESI-HRMS; FT-IR for compound 27

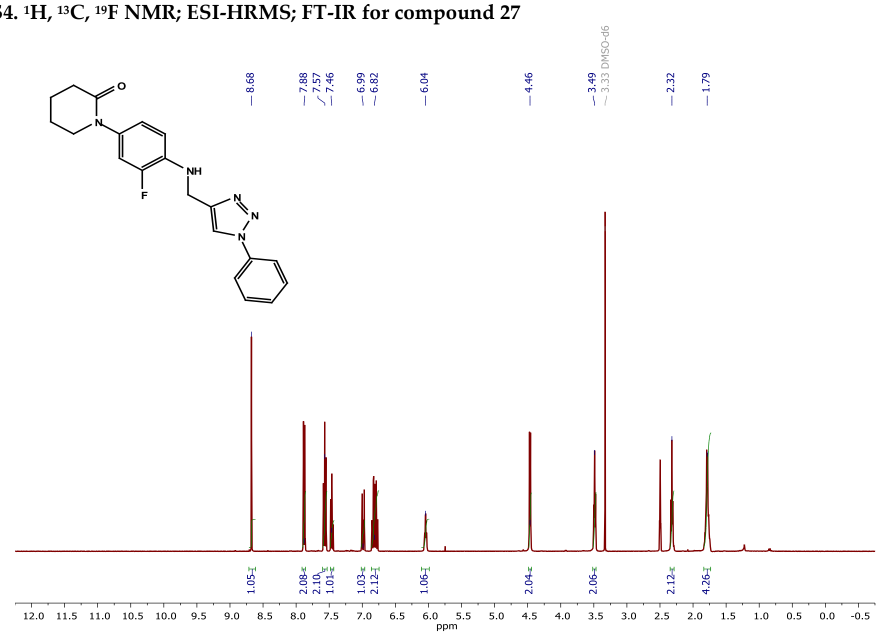

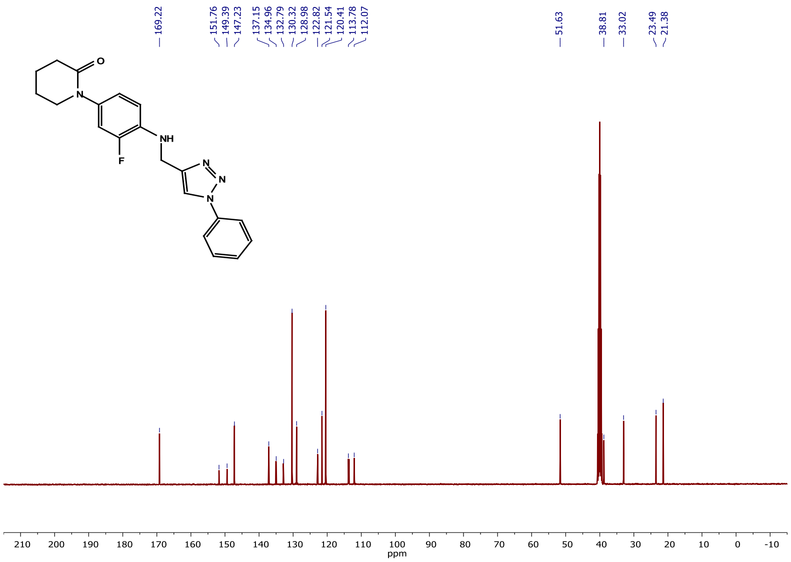
169.22
151.76
149.39
147.23
137.15
134.96
132.79
130.32
128.98
122.82
121.54
120.41
113.78
112.07
51.63
38.81
33.02
23.49
21.38
ppm

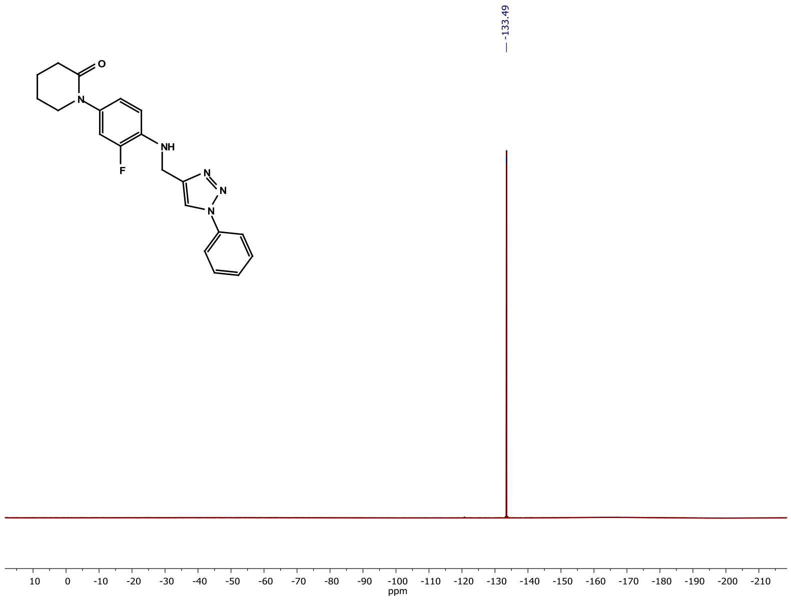
-133.49
ppm

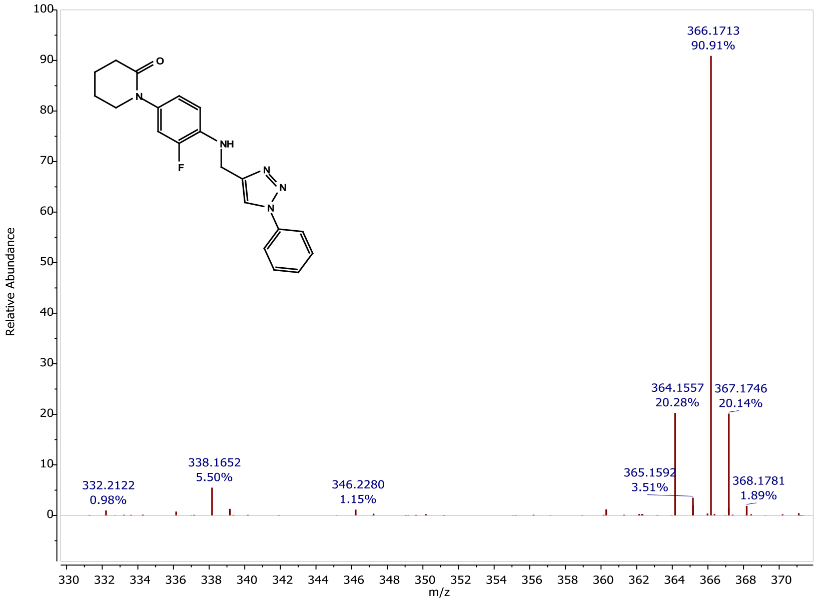

Relative Abundance
m/z
366.1713
90.91%
364.1557
20.28%
367.1746
20.14%
365.1592
3.51%
368.1781
1.89%
338.1652
5.50%
332.2122
0.98%
346.2280
1.15%

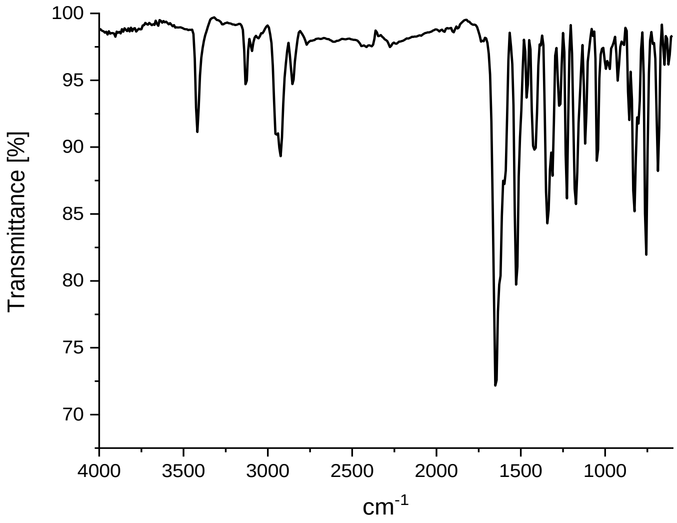

Transmittance [%]
cm-1

S5. ^{1}H, ^{13}C, ^{19}F NMR; ESI-HRMS; FT-IR for compound 28

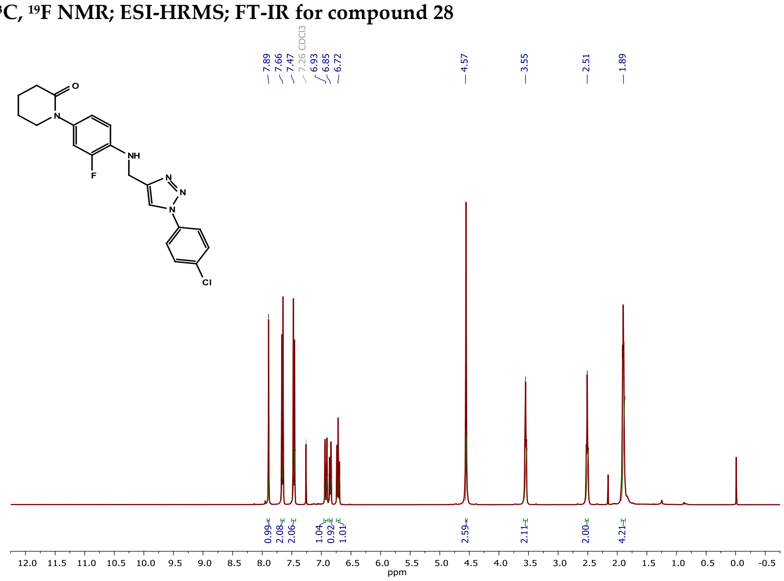

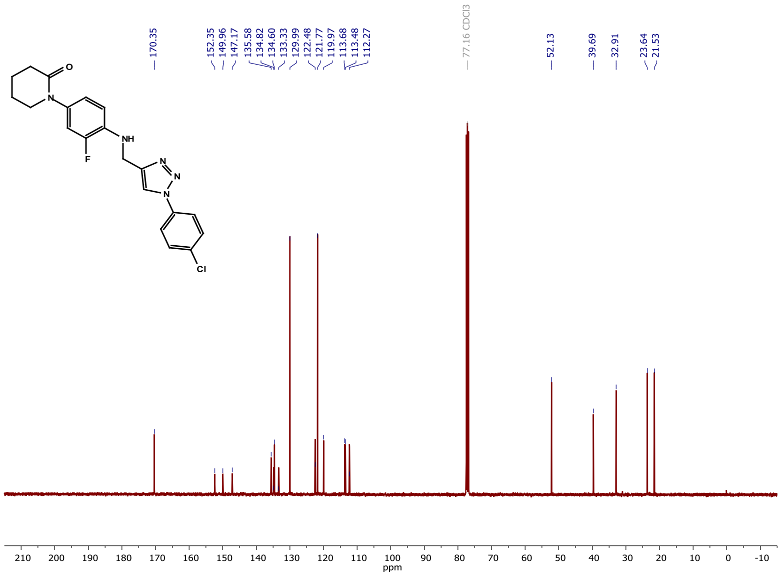

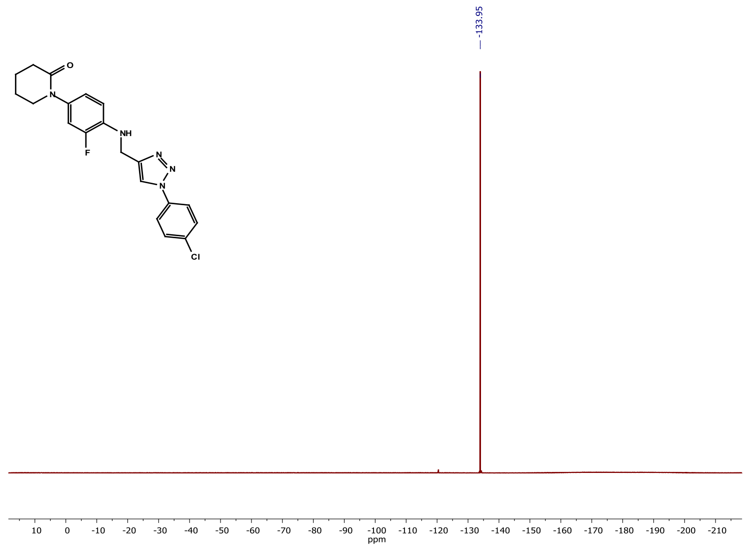
-133.95
O
N
NH
F
N
N
N
Cl
10
0
-10
-20
-30
-40
-50
-60
-70
-80
-90
-100
-110
-120
-130
-140
-150
-160
-170
-180
-190
-200
-210
ppm

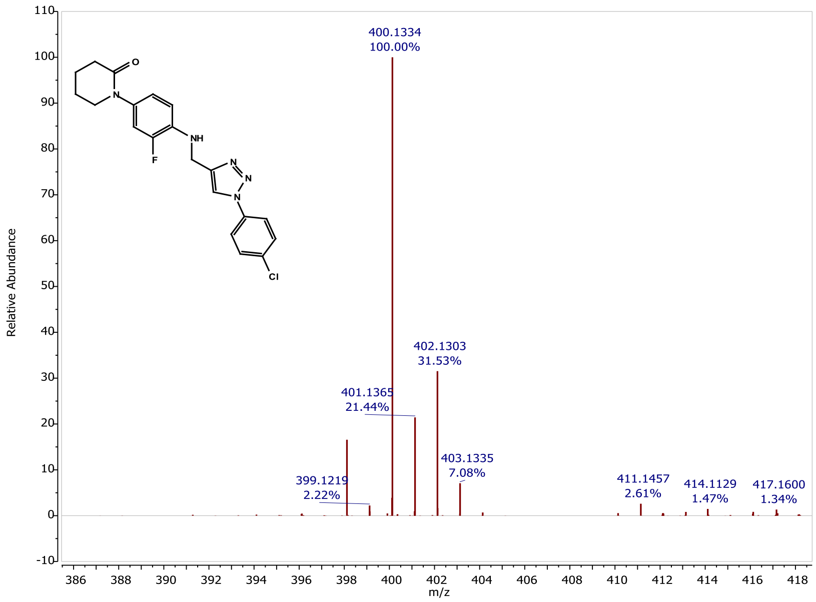
Relative Abundance
m/z
400.1334
100.00%
402.1303
31.53%
401.1365
21.44%
403.1335
7.08%
399.1219
2.22%
411.1457
2.61%
414.1129
1.47%
417.1600
1.34%
O
N
NH
F
N
N
N
Cl

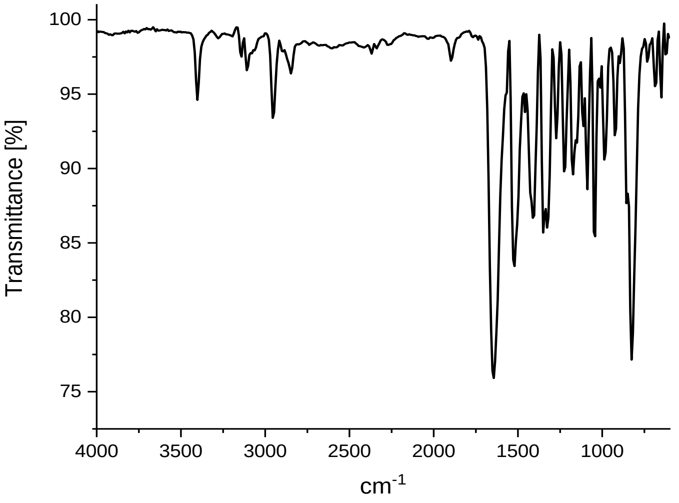

S6. ^{1}H, ^{13}C, ^{19}F NMR; ESI-HRMS; FT-IR for compound 29

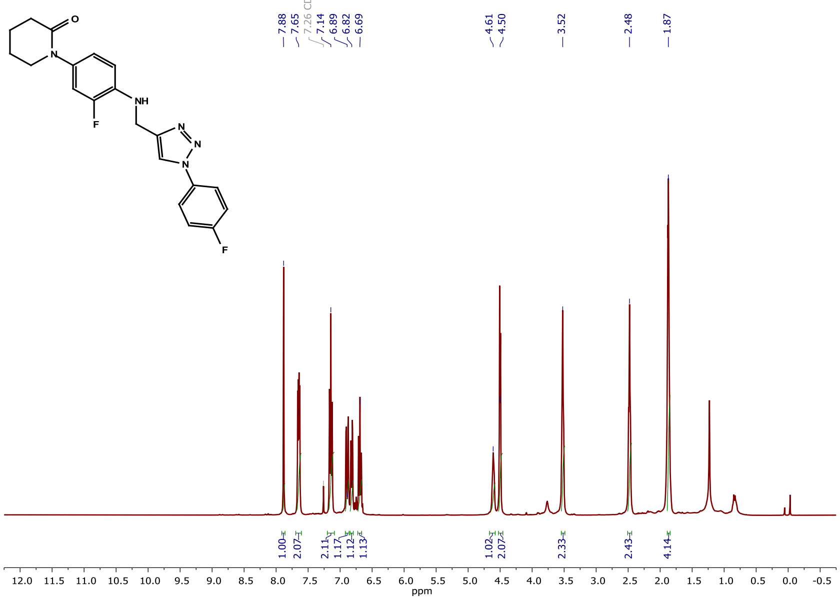

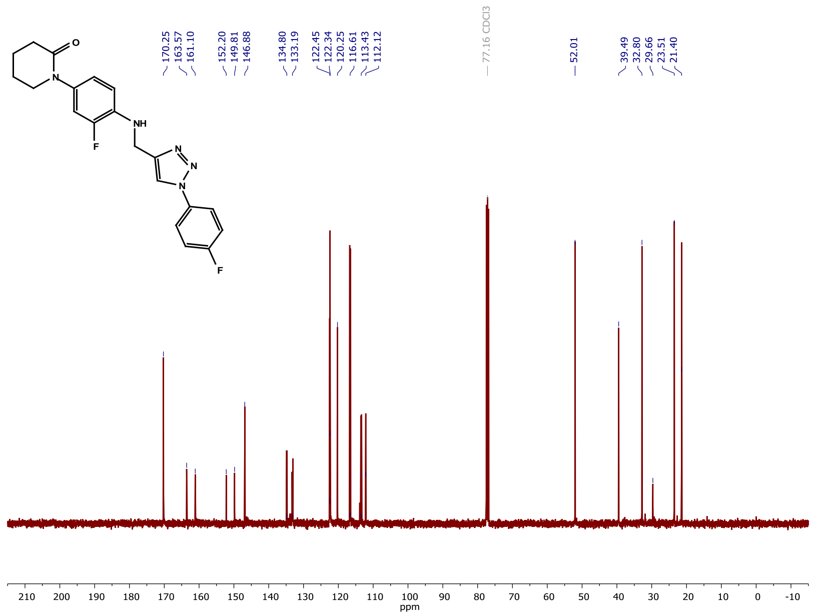

170.25
163.57
161.10
152.20
149.81
146.88
134.80
133.19
122.45
122.34
120.25
116.61
113.43
112.12
77.16 CDCl3
52.01
39.49
32.80
29.66
23.51
21.40
210 200 190 180 170 160 150 140 130 120 110 100 90 80 70 60 50 40 30 20 10 0 -10
ppm

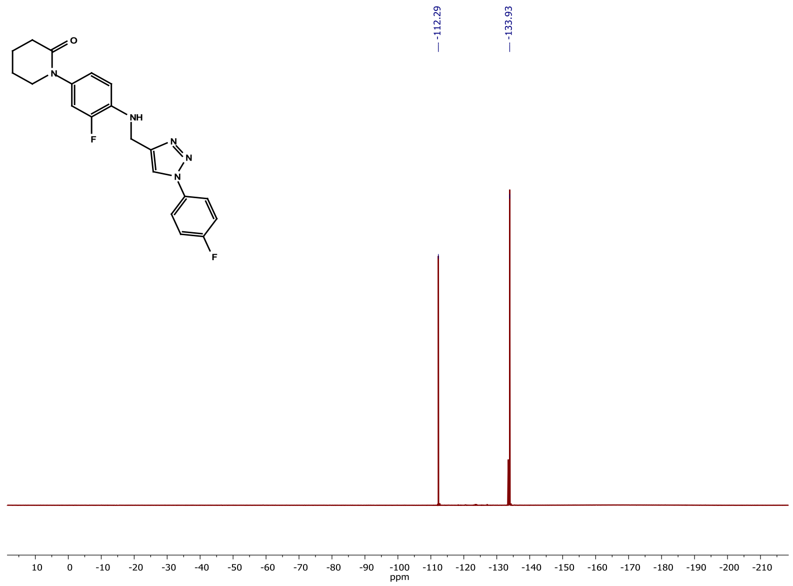

-112.29
-133.93
10 0 -10 -20 -30 -40 -50 -60 -70 -80 -90 -100 -110 -120 -130 -140 -150 -160 -170 -180 -190 -200 -210
ppm

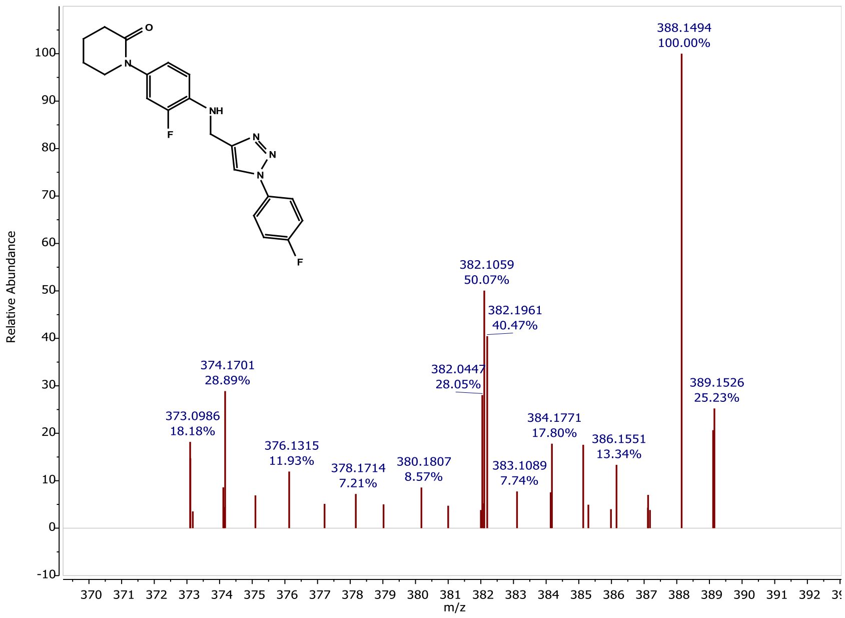

388.1494
100.00%
382.1059
50.07%
382.1961
40.47%
374.1701
28.89%
382.0447
28.05%
389.1526
25.23%
373.0986
18.18%
384.1771
17.80%
386.1551
13.34%
376.1315
11.93%
380.1807
8.57%
383.1089
7.74%
378.1714
7.21%
Relative Abundance
m/z

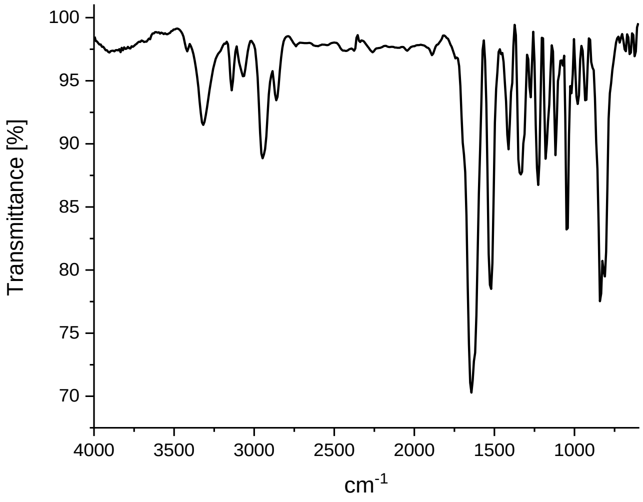

Transmittance [%]
cm-1

S7. ^{1}H, ^{13}C, ^{19}F NMR; ESI-HRMS; FT-IR for compound 30

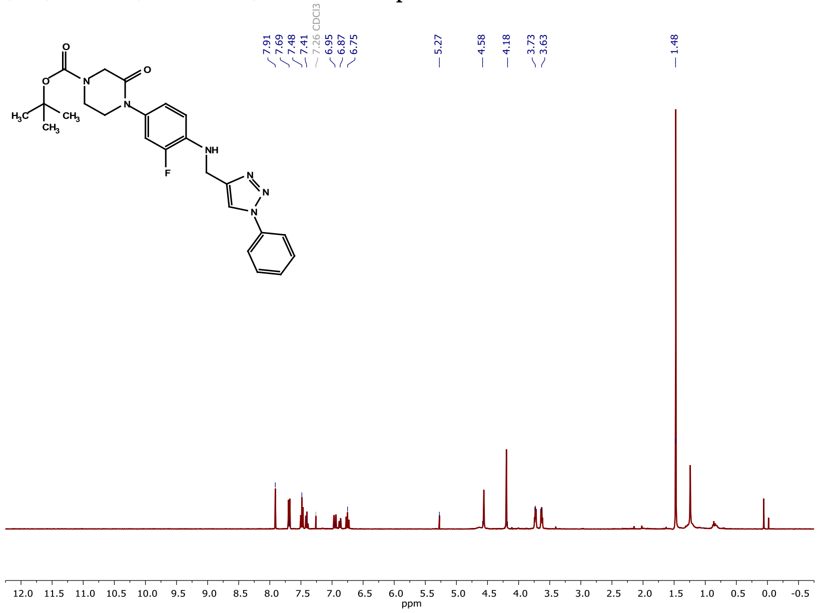

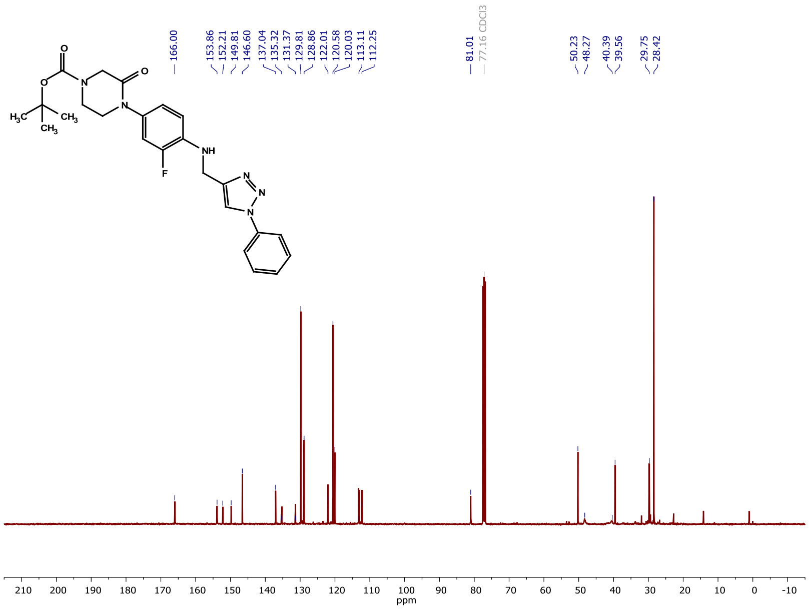

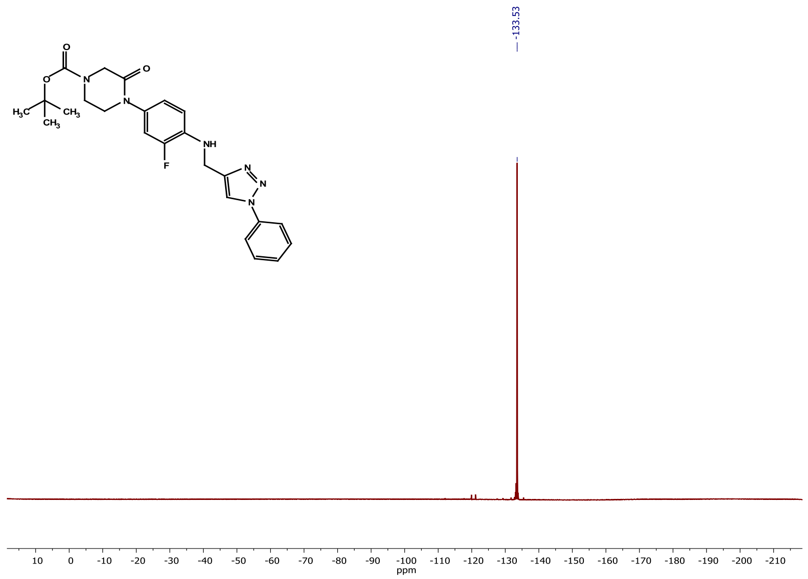

—133.53
10
0
-10
-20
-30
-40
-50
-60
-70
-80
-90
-100
-110
-120
-130
-140
-150
-160
-170
-180
-190
-200
-210
ppm

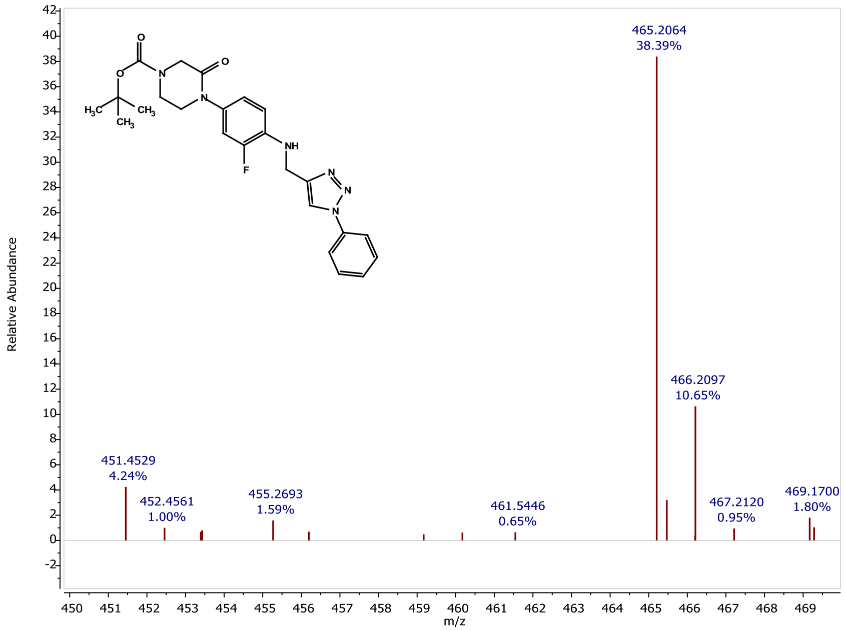

465.2064
38.39%
466.2097
10.65%
451.4529
4.24%
452.4561
1.00%
455.2693
1.59%
461.5446
0.65%
467.2120
0.95%
469.1700
1.80%
Relative Abundance
m/z

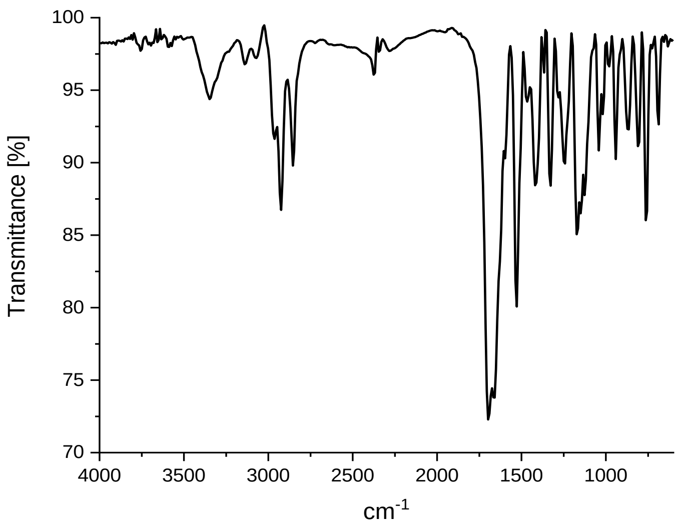

S8. ^{1}H, ^{13}C, ^{19}F NMR; ESI-HRMS; FT-IR for compound 31

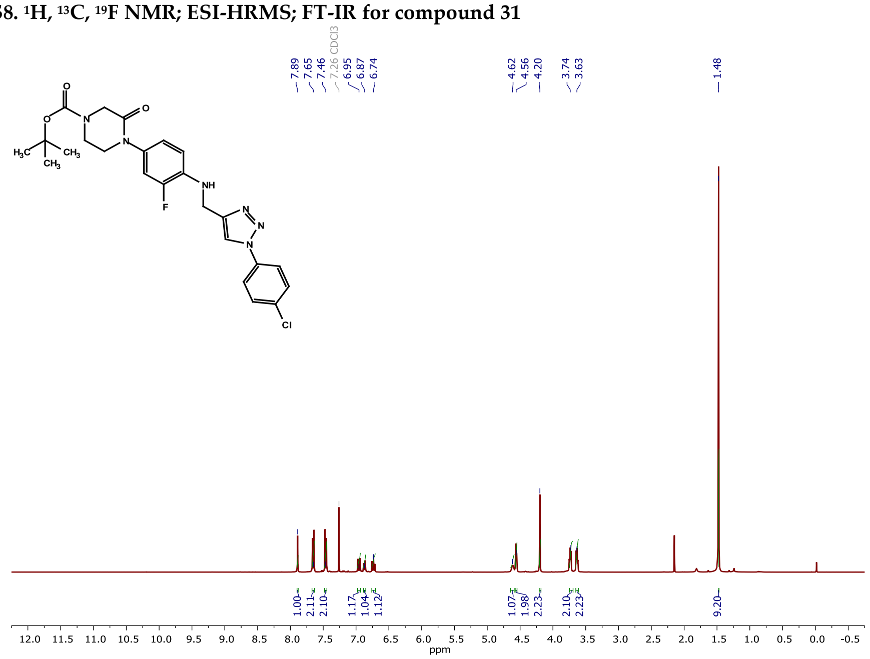

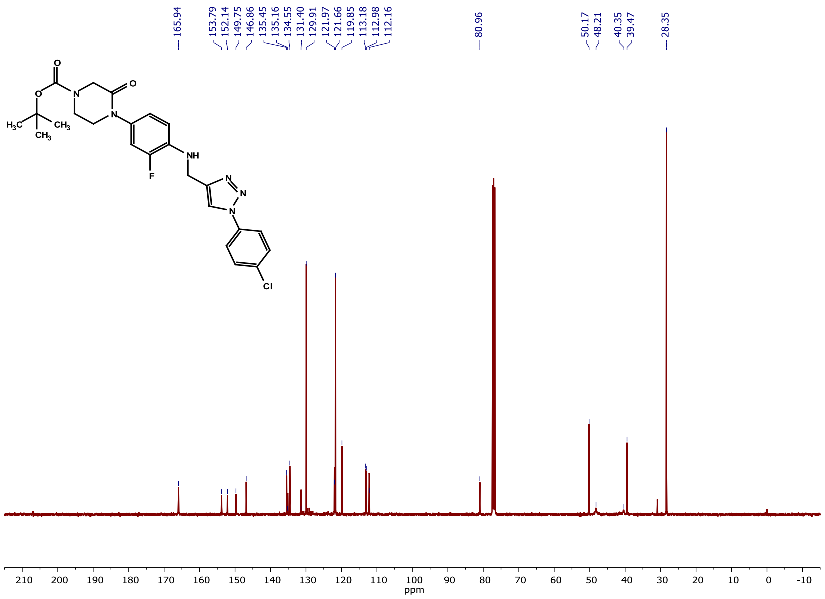
165.94
153.79
152.14
149.75
146.86
135.45
135.16
134.55
131.40
129.91
121.97
121.66
119.85
113.18
112.98
112.16
80.96
50.17
48.21
40.35
39.47
28.35
O
N
O
O
H3C
CH3
CH3
N
F
NH
N
N
N
Cl
210 200 190 180 170 160 150 140 130 120 110 100 90 80 70 60 50 40 30 20 10 0 -10
ppm

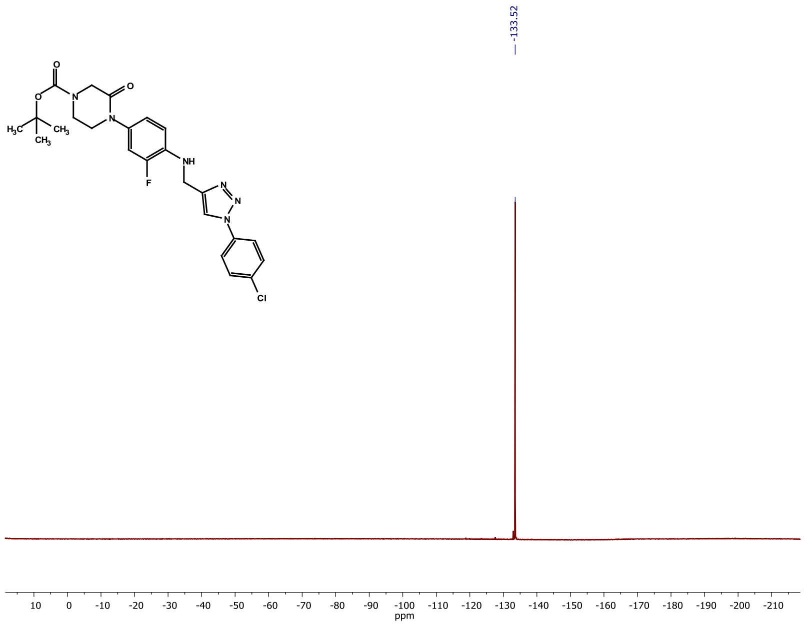
-133.52
O
N
O
O
H3C
CH3
CH3
N
F
NH
N
N
N
Cl
10 0 -10 -20 -30 -40 -50 -60 -70 -80 -90 -100 -110 -120 -130 -140 -150 -160 -170 -180 -190 -200 -210
ppm

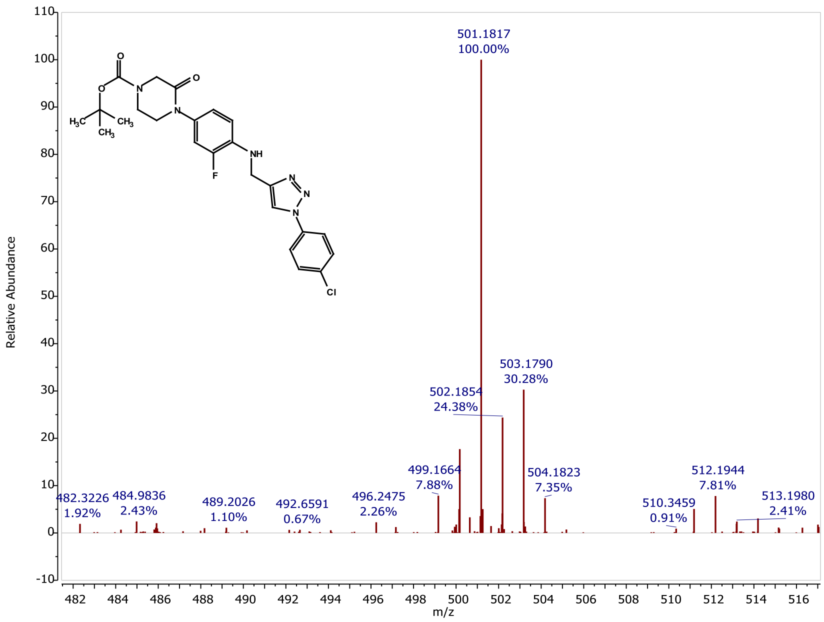
501.1817
100.00%
503.1790
30.28%
502.1854
24.38%
499.1664
7.88%
504.1823
7.35%
512.1944
7.81%
513.1980
2.41%
510.3459
0.91%
482.3226
1.92%
484.9836
2.43%
489.2026
1.10%
492.6591
0.67%
496.2475
2.26%
Relative Abundance
m/z

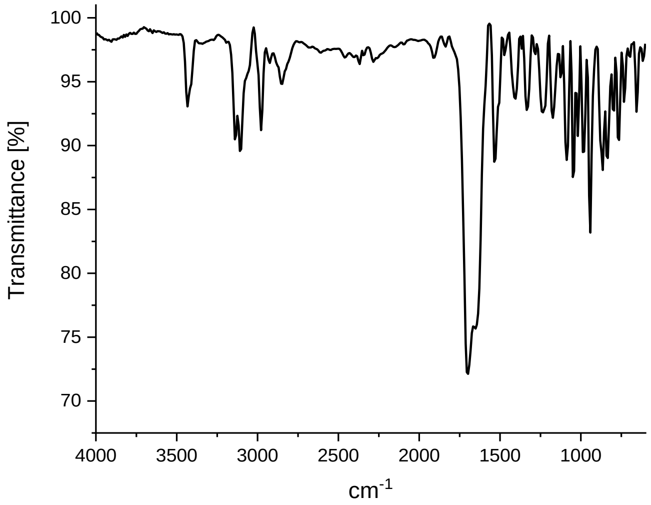
Transmittance [%]
cm^{-1}

S9. ^{1}H, ^{13}C, ^{19}F NMR; ESI-HRMS; FT-IR for compound 32

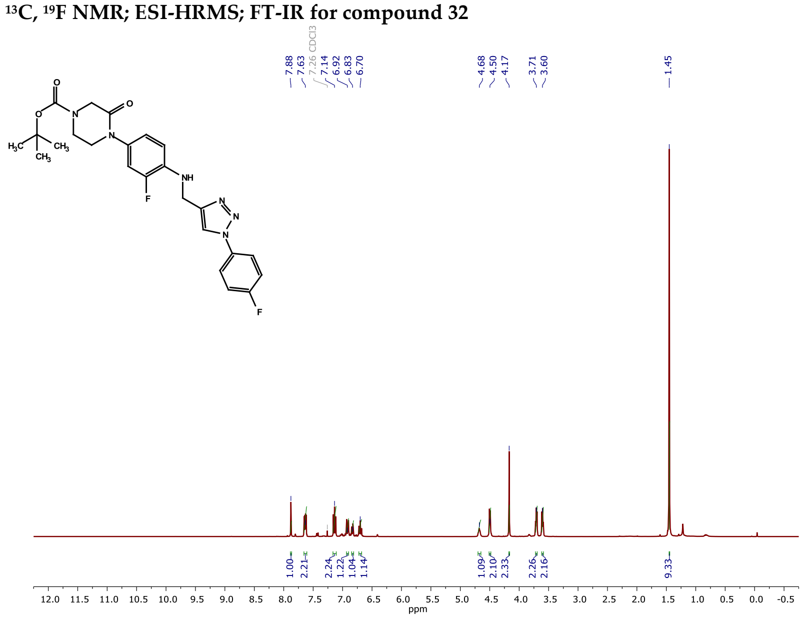

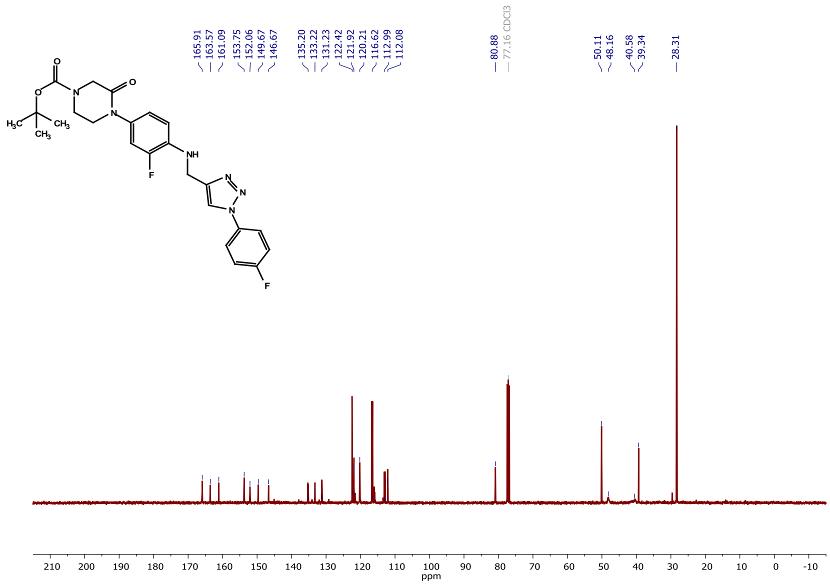

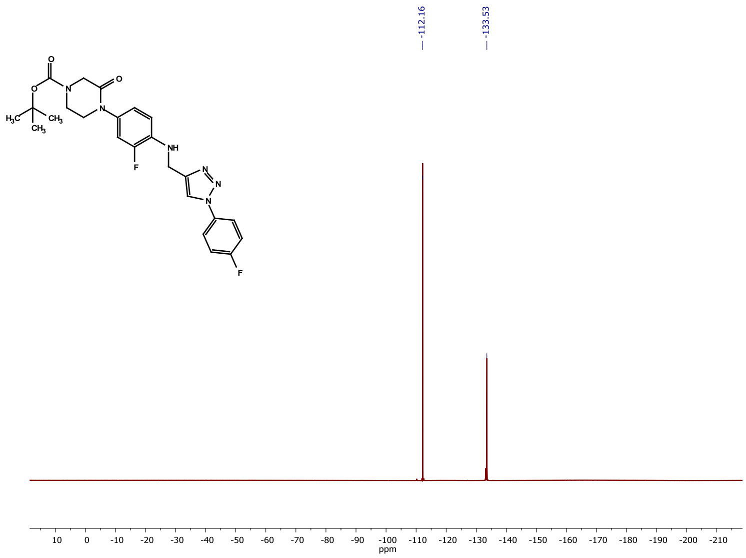

-112.16
-133.53
ppm

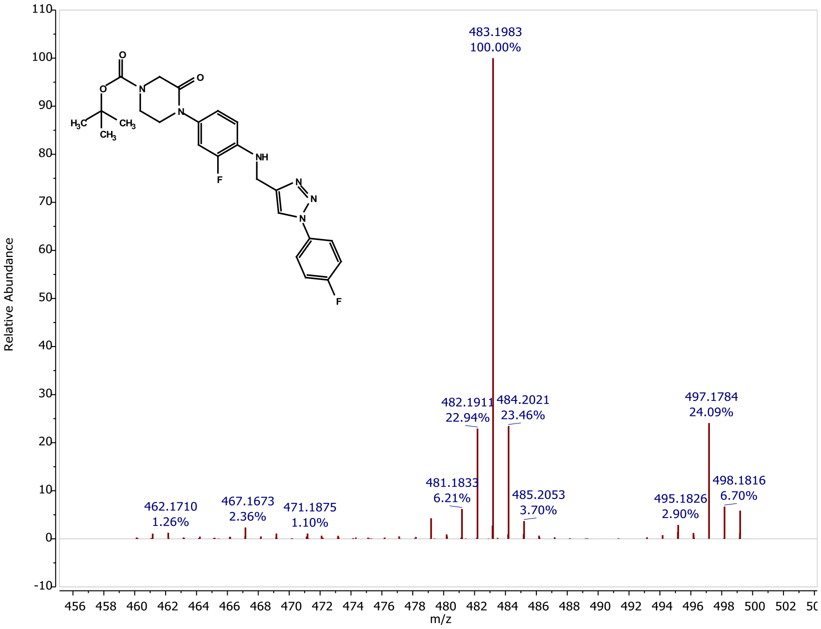

483.1983
100.00%
482.1911
22.94%
484.2021
23.46%
497.1784
24.09%
481.1833
6.21%
485.2053
3.70%
495.1826
2.90%
498.1816
6.70%
462.1710
1.26%
467.1673
2.36%
471.1875
1.10%
Relative Abundance
m/z

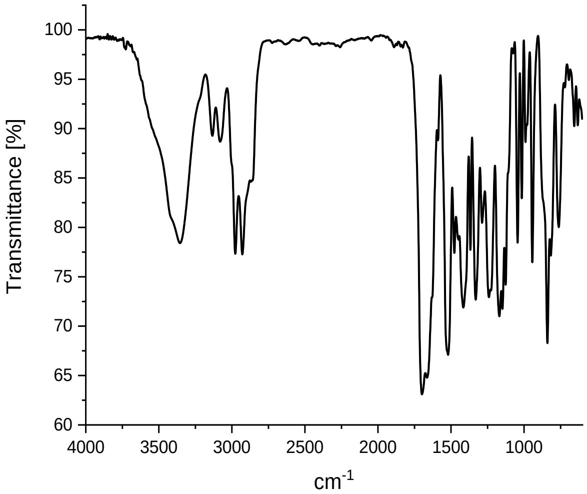

S10. ^{1}H, ^{13}C, ^{19}F NMR; ESI-HRMS; FT-IR for compound 33

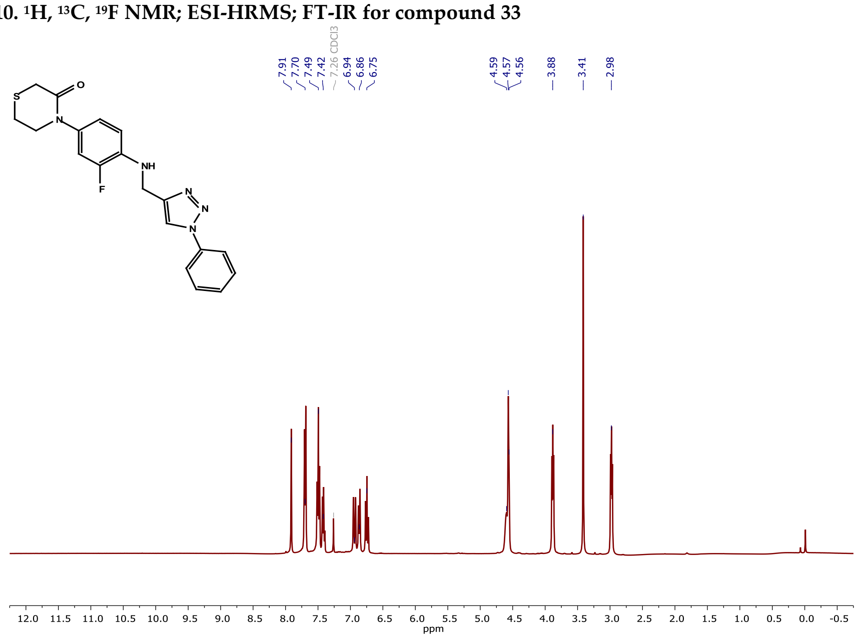

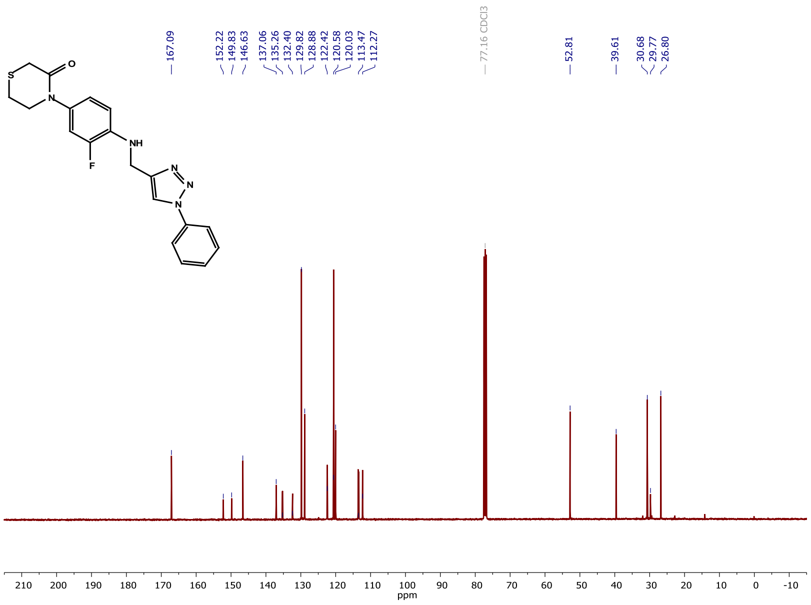

167.09
152.22
149.83
146.63
137.06
135.26
132.40
129.82
128.88
122.42
120.58
120.03
113.47
112.27
77.16 CDCl3
52.81
39.61
30.68
29.77
26.80
S
O
N
NH
F
N
N
N
ppm

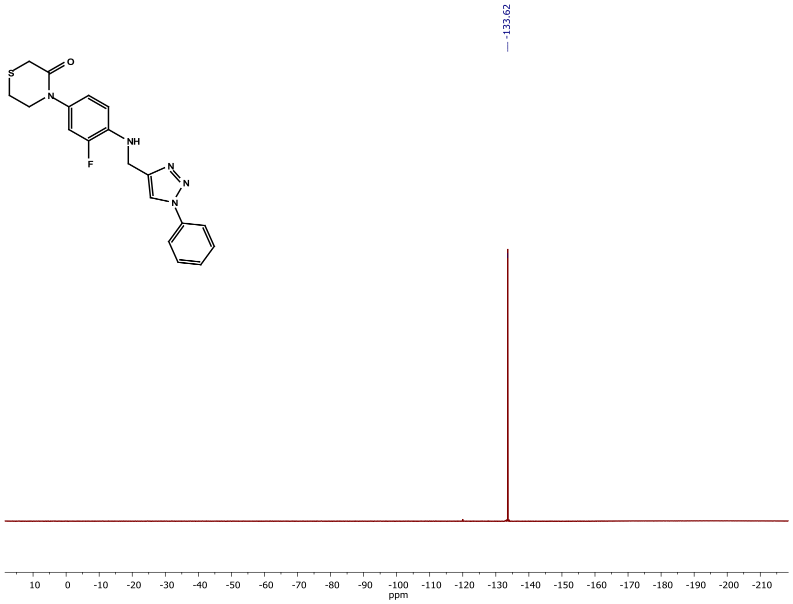

-133.62
S
O
N
NH
F
N
N
N
ppm

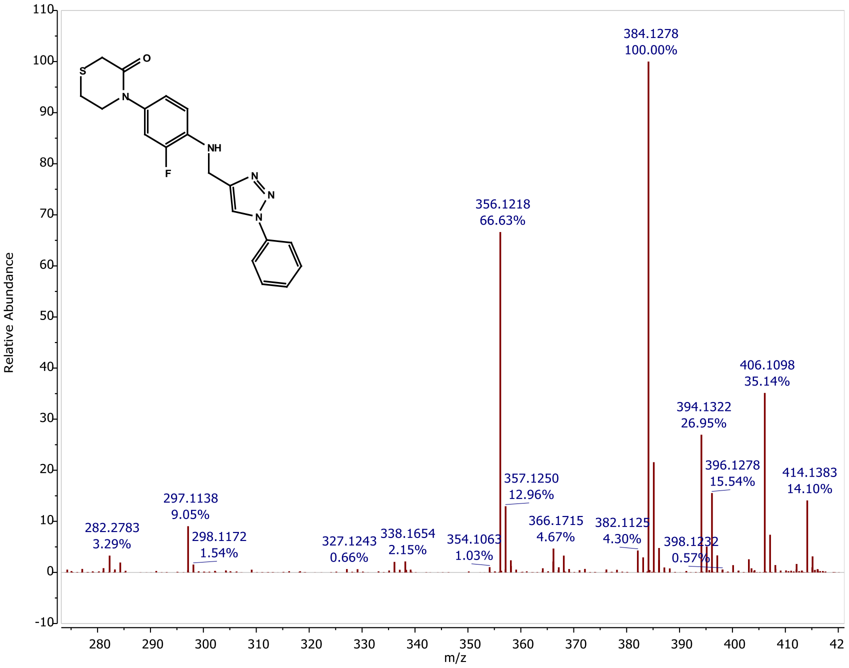

384.1278
100.00%
356.1218
66.63%
406.1098
35.14%
394.1322
26.95%
396.1278
15.54%
414.1383
14.10%
357.1250
12.96%
297.1138
9.05%
282.2783
3.29%
298.1172
1.54%
327.1243
0.66%
338.1654
2.15%
354.1063
1.03%
366.1715
4.67%
382.1125
4.30%
398.1232
0.57%
Relative Abundance
m/z

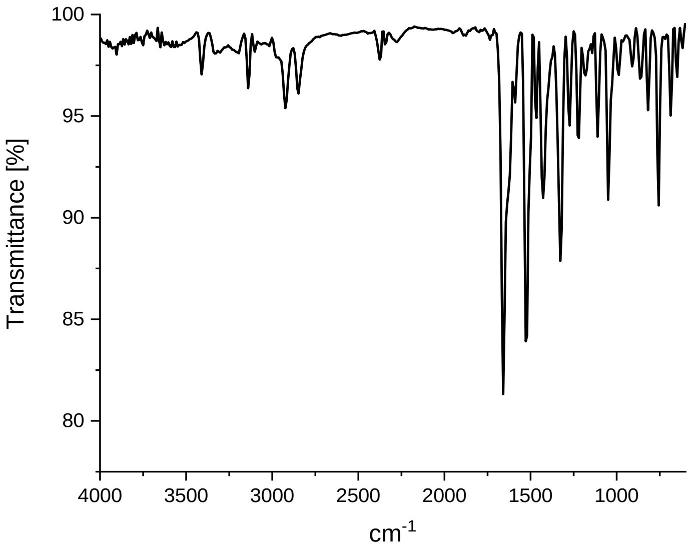

Transmittance [%]
cm-1

S11. ^{1}H, ^{13}C, ^{19}F NMR; ESI-HRMS; FT-IR for compound 34

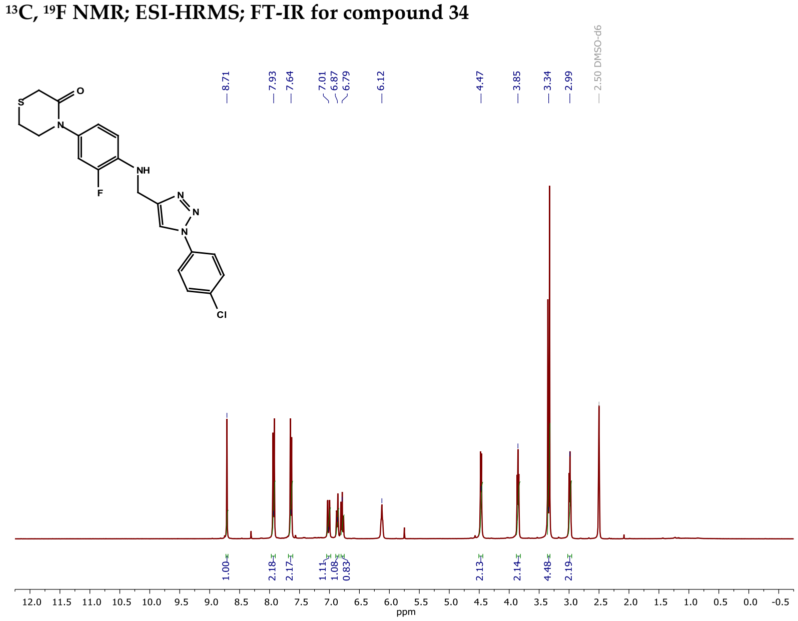

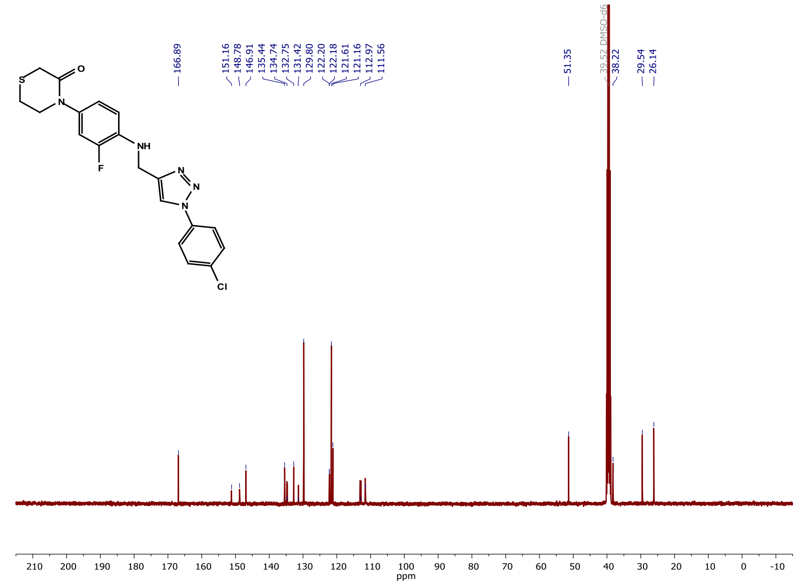

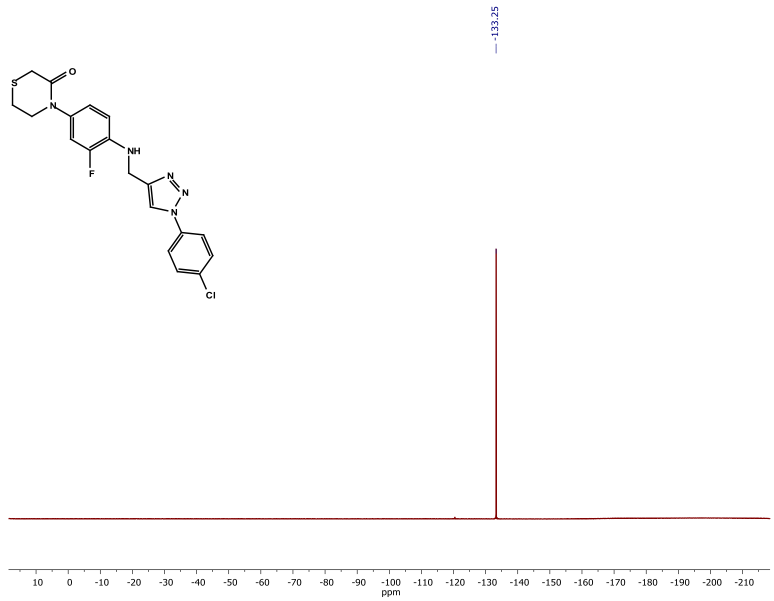
-133.25
S
O
N
NH
F
N
N
N
Cl
10 0 -10 -20 -30 -40 -50 -60 -70 -80 -90 -100 -110 -120 -130 -140 -150 -160 -170 -180 -190 -200 -210
ppm

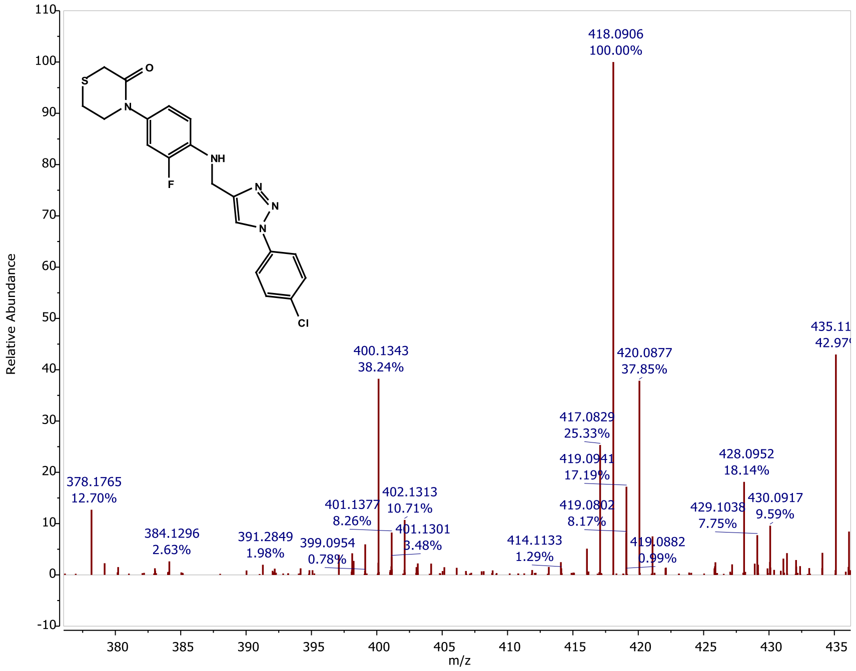
Relative Abundance
m/z
418.0906
100.00%
400.1343
38.24%
420.0877
37.85%
435.11
42.97
417.0829
25.33%
419.0941
17.19%
428.0952
18.14%
378.1765
12.70%
402.1313
10.71%
401.1377
8.26%
401.1301
3.48%
419.0802
8.17%
429.1038
7.75%
430.0917
9.59%
384.1296
2.63%
391.2849
1.98%
399.0954
0.78%
414.1133
1.29%
419.0882
0.99%

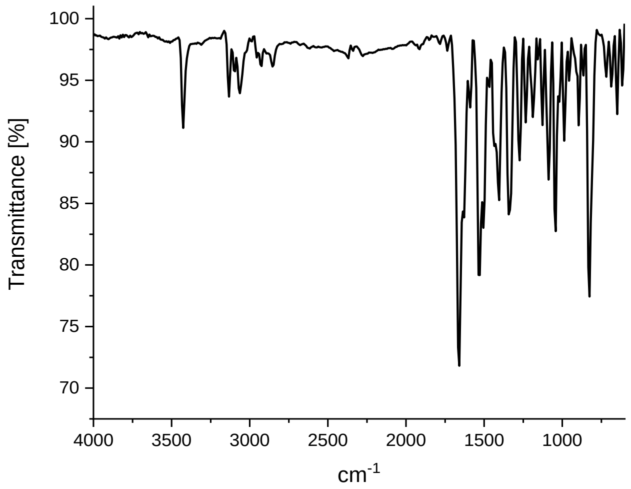

S12. ^{1}H, ^{13}C, ^{19}F NMR; ESI-HRMS; FT-IR for compound 35

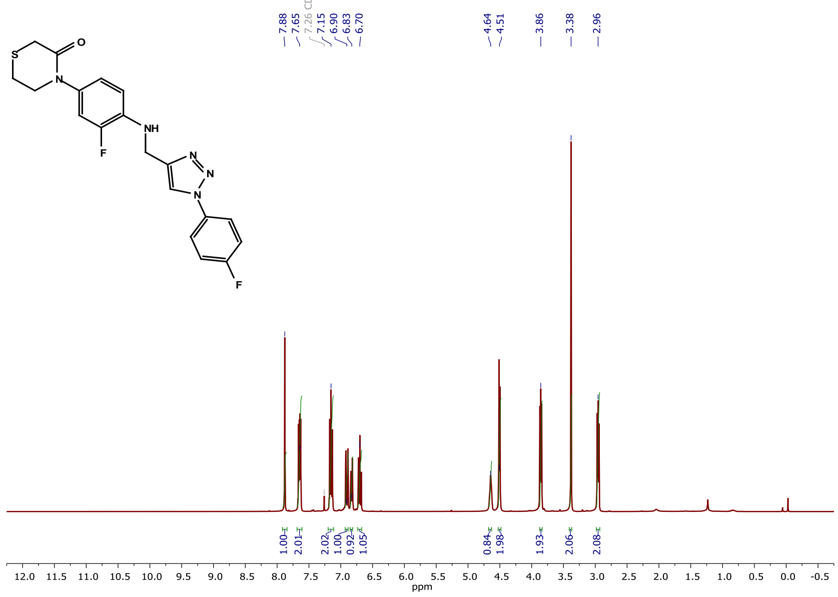

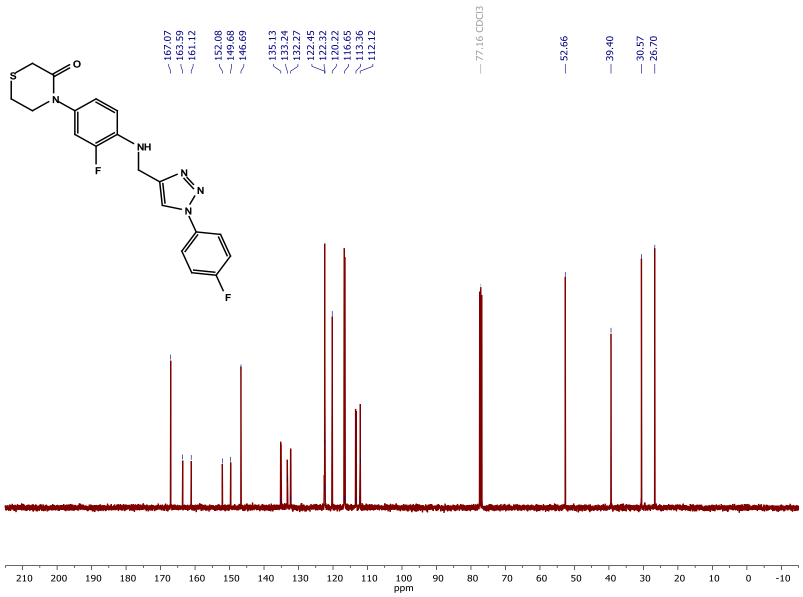

167.07
163.59
161.12
152.08
149.68
146.69
135.13
133.24
132.27
122.45
122.32
120.22
116.65
113.36
112.12
77.16 CDCl3
52.66
39.40
30.57
26.70
ppm

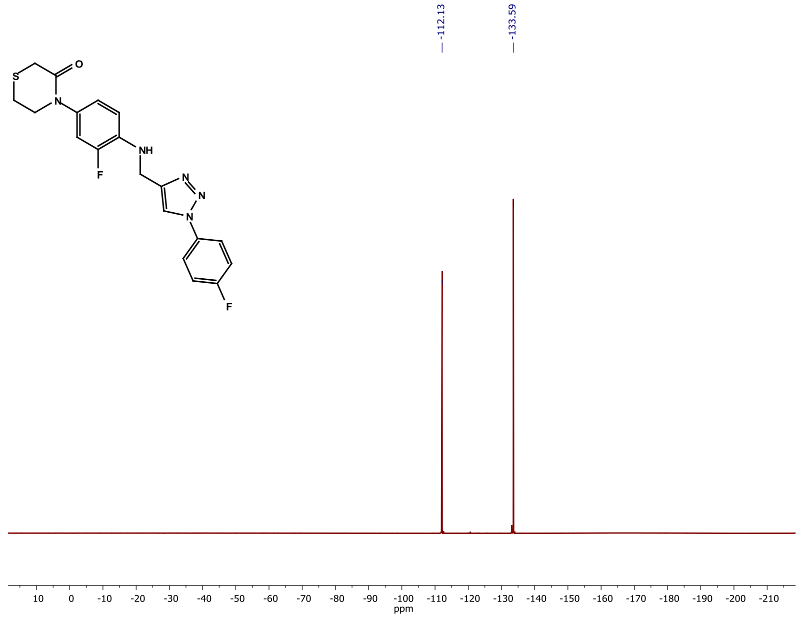

-112.13
-133.59
ppm

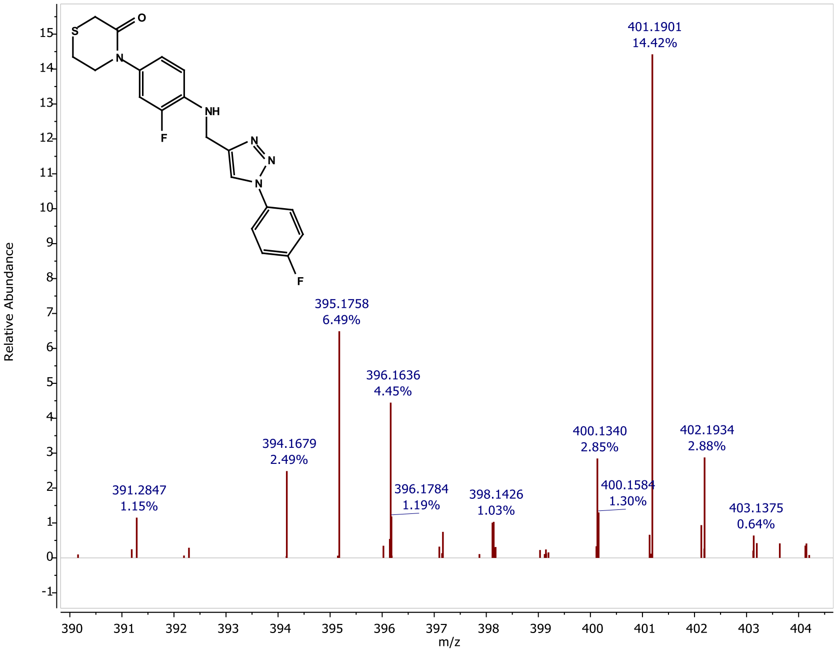

401.1901
14.42%
395.1758
6.49%
396.1636
4.45%
394.1679
2.49%
400.1340
2.85%
402.1934
2.88%
391.2847
1.15%
396.1784
1.19%
398.1426
1.03%
400.1584
1.30%
403.1375
0.64%
Relative Abundance
m/z

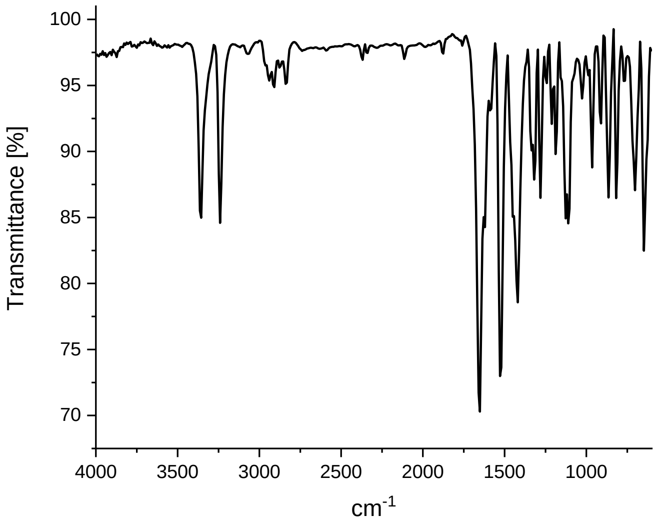

Transmittance [%]
cm-1

S13. ^{1}H, ^{13}C, ^{19}F NMR; ESI-HRMS; FT-IR for compound 36

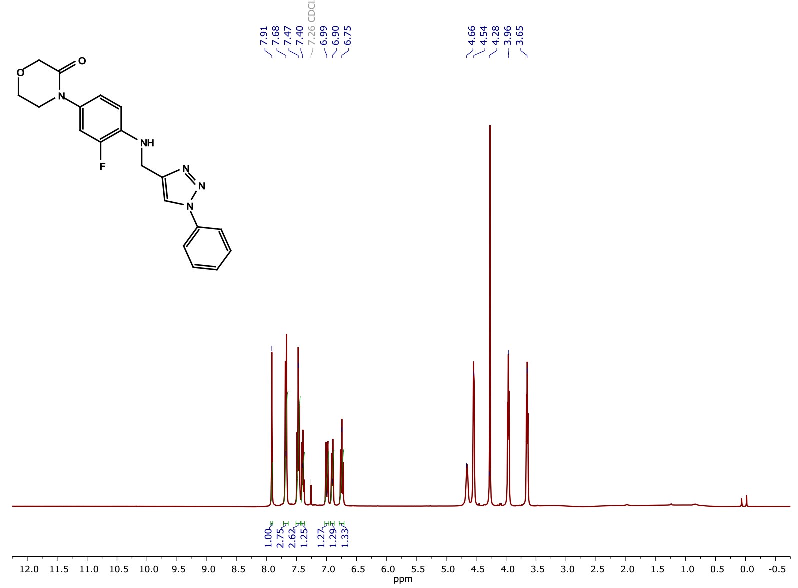

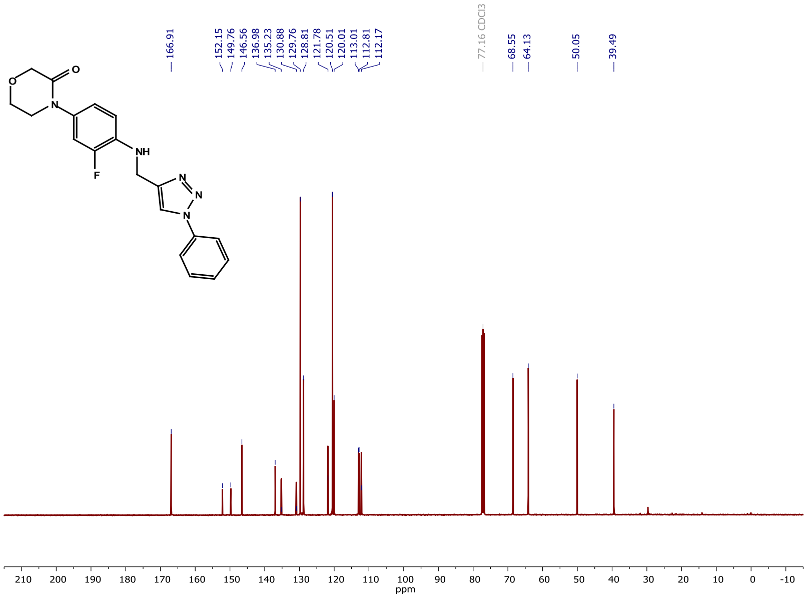

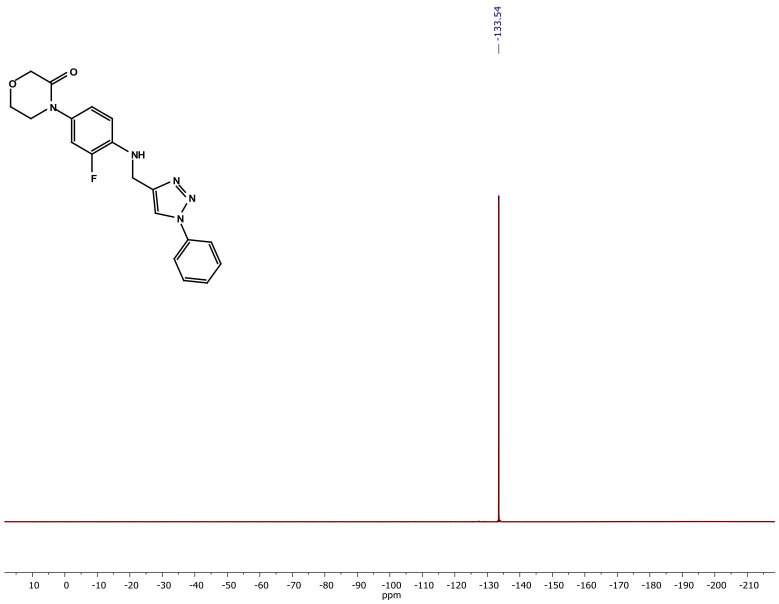

-133.54
O
N
NH
F
N
N
N
10
0
-10
-20
-30
-40
-50
-60
-70
-80
-90
-100
-110
-120
-130
-140
-150
-160
-170
-180
-190
-200
-210
ppm

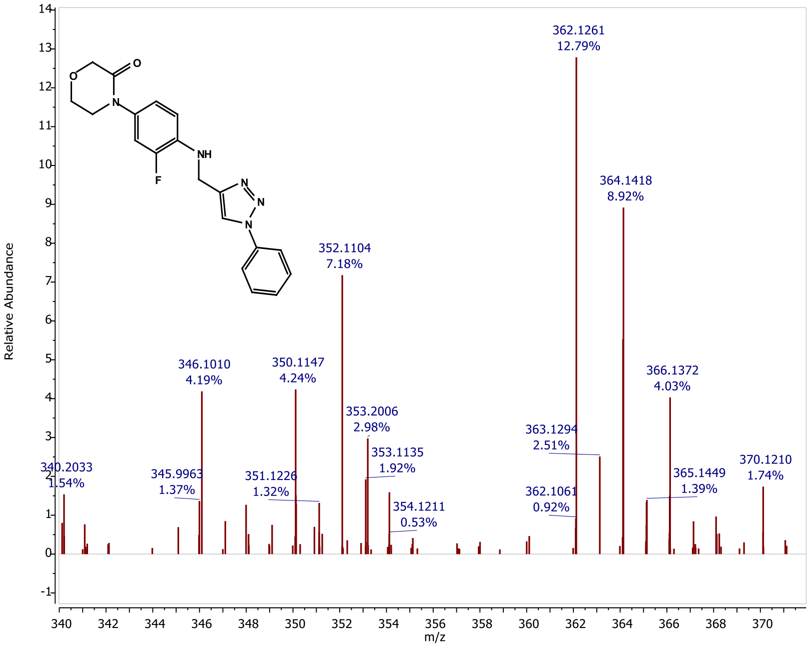

Relative Abundance
m/z
362.1261
12.79%
364.1418
8.92%
352.1104
7.18%
346.1010
4.19%
350.1147
4.24%
366.1372
4.03%
353.2006
2.98%
363.1294
2.51%
353.1135
1.92%
340.2033
1.54%
345.9963
1.37%
351.1226
1.32%
365.1449
1.39%
370.1210
1.74%
362.1061
0.92%
354.1211
0.53%

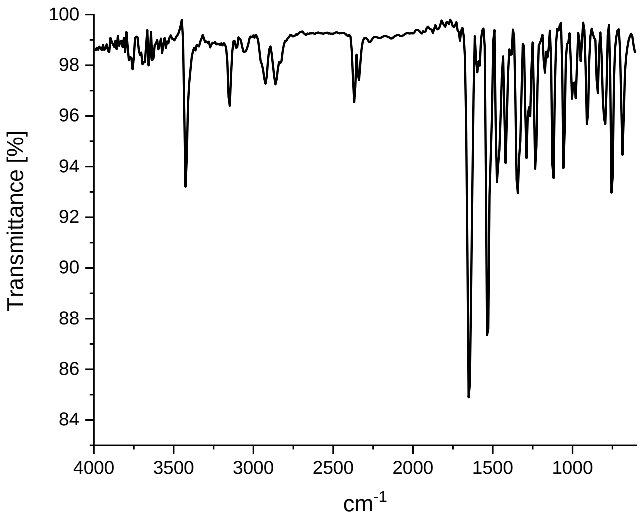

S14. ^{1}H, ^{13}C, ^{19}F NMR; ESI-HRMS; FT-IR for compound 37

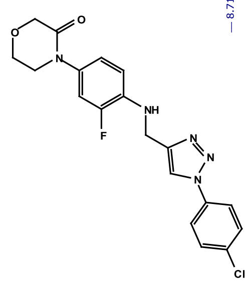

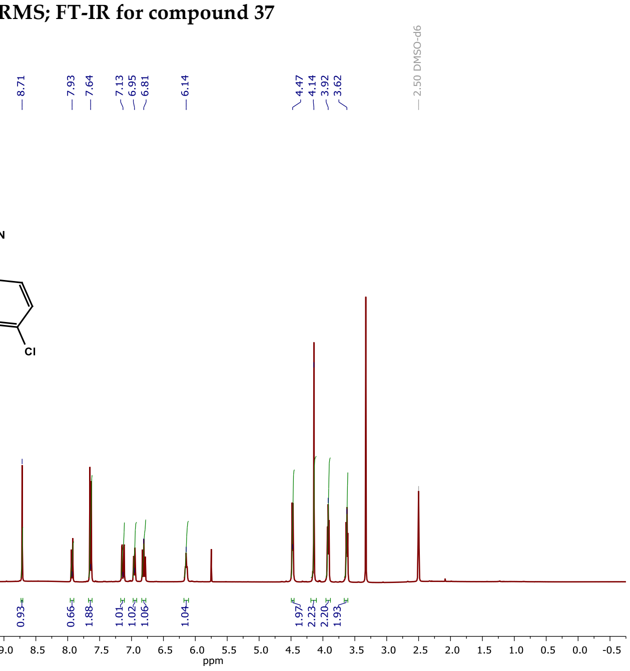

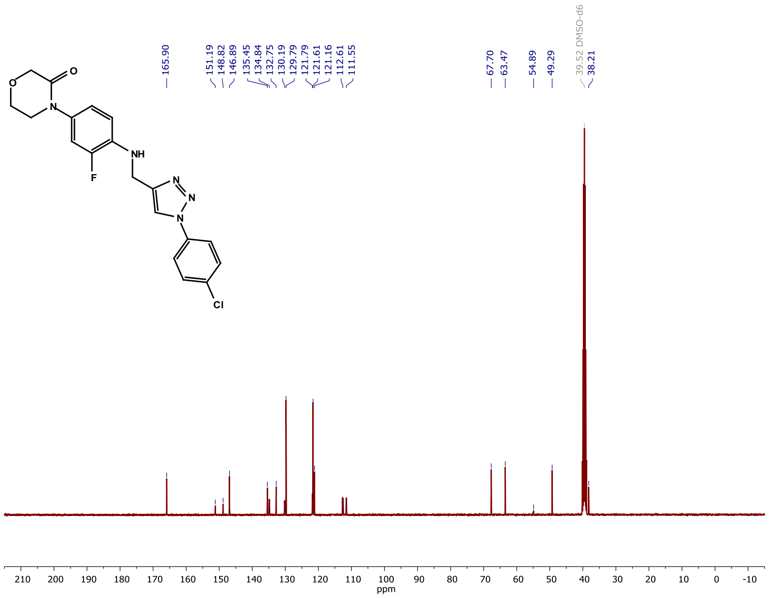
165.90
151.19
148.82
146.89
135.45
134.84
132.75
130.19
129.79
121.79
121.61
121.16
112.61
111.55
67.70
63.47
54.89
49.29
39.52 DMSO-d6
38.21
ppm
O
N
NH
F
N
N
N
Cl

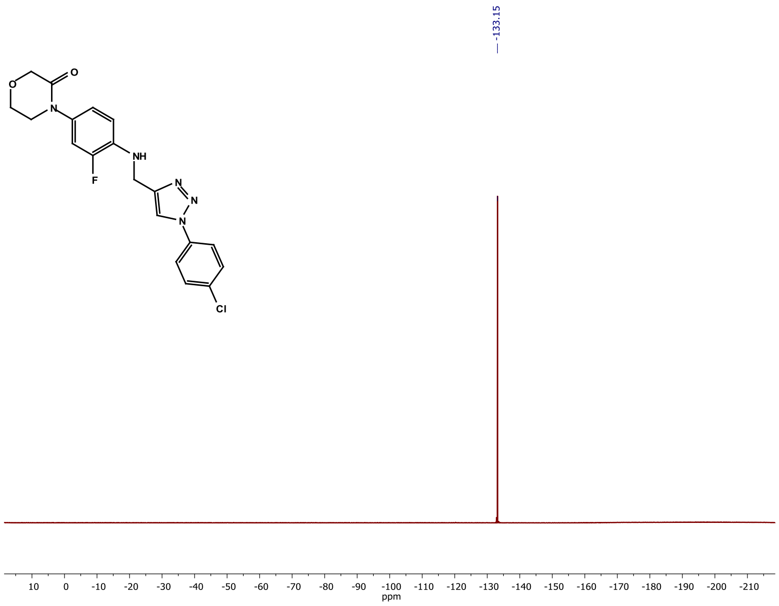
-133.15
ppm
O
N
NH
F
N
N
N
Cl

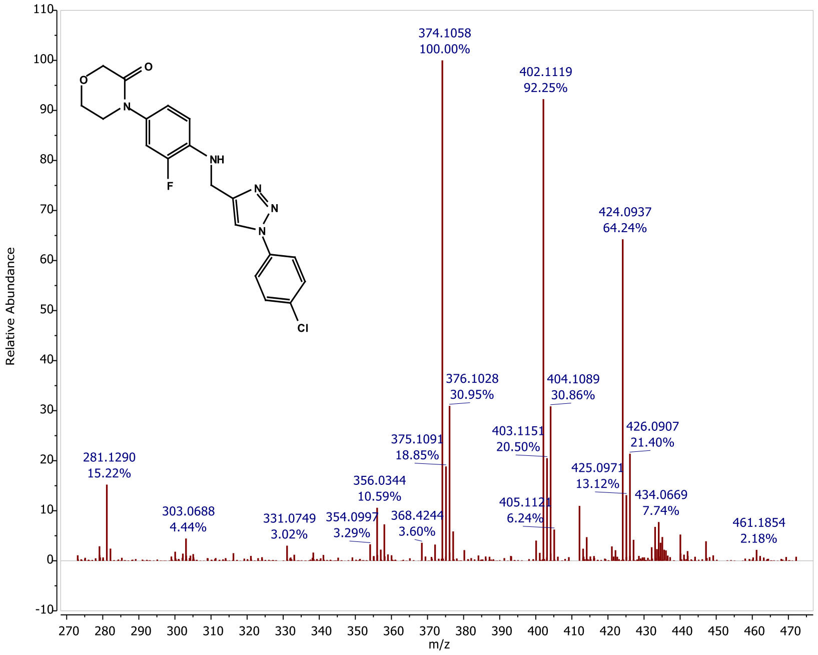
Relative Abundance
m/z
374.1058
100.00%
402.1119
92.25%
424.0937
64.24%
376.1028
30.95%
404.1089
30.86%
426.0907
21.40%
403.1151
20.50%
375.1091
18.85%
281.1290
15.22%
425.0971
13.12%
356.0344
10.59%
434.0669
7.74%
405.1121
6.24%
303.0688
4.44%
368.4244
3.60%
354.0997
3.29%
331.0749
3.02%
461.1854
2.18%

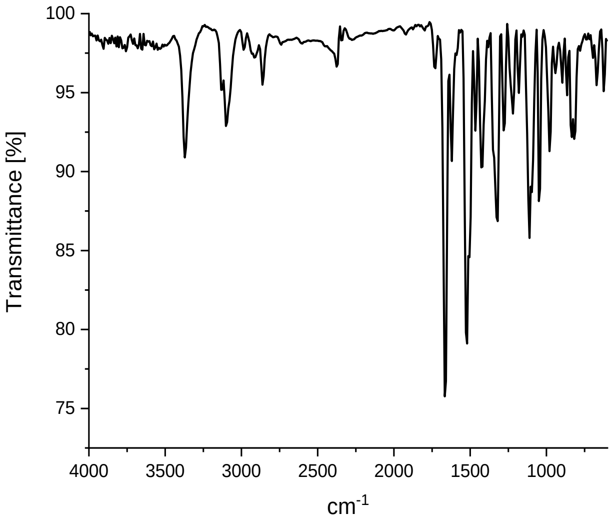
Transmittance [%]
cm-1

S15. ¹H, ¹³C, ¹⁹F NMR; ESI-HRMS; FT-IR for compound 38

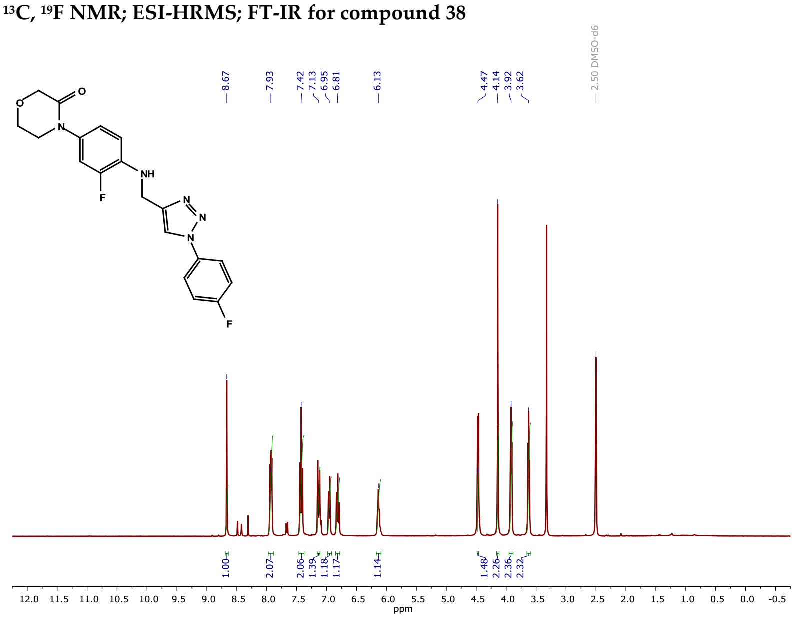

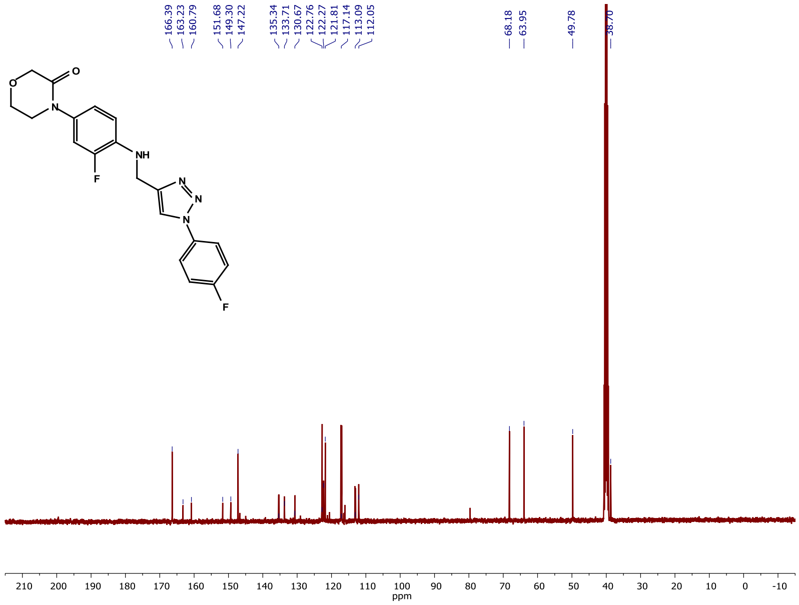

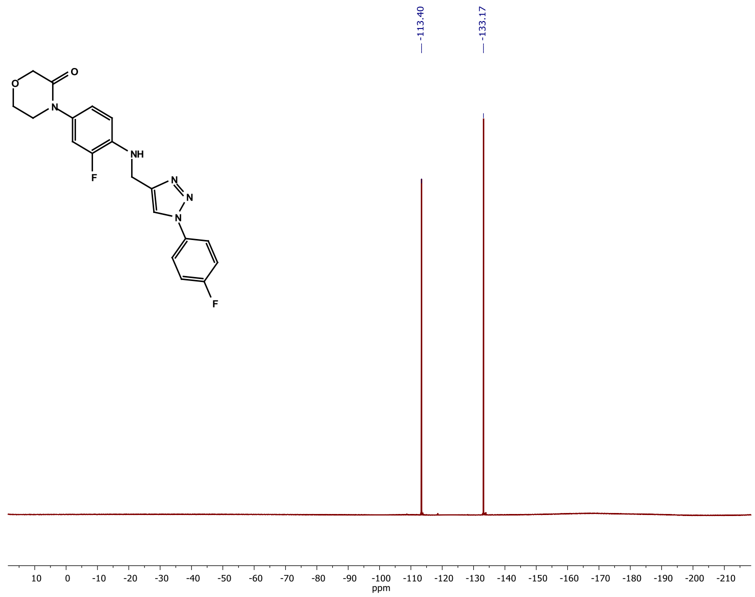

-113.40
-133.17
O
O
N
NH
F
N
N
N
F
10 0 -10 -20 -30 -40 -50 -60 -70 -80 -90 -100 -110 -120 -130 -140 -150 -160 -170 -180 -190 -200 -210
ppm

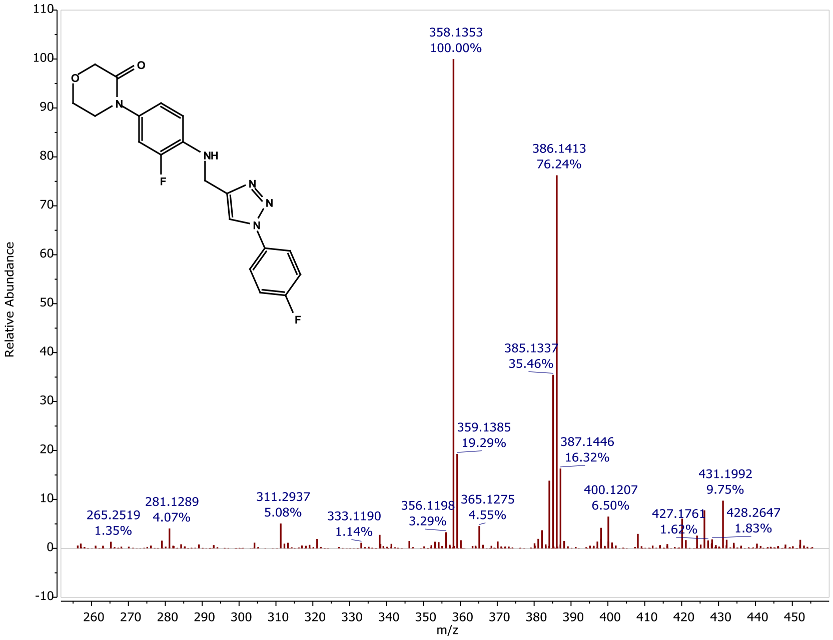

Relative Abundance
m/z
358.1353
100.00%
386.1413
76.24%
385.1337
35.46%
359.1385
19.29%
387.1446
16.32%
431.1992
9.75%
400.1207
6.50%
265.2519
1.35%
281.1289
4.07%
311.2937
5.08%
333.1190
1.14%
356.1198
3.29%
365.1275
4.55%
427.1761
1.62%
428.2647
1.83%

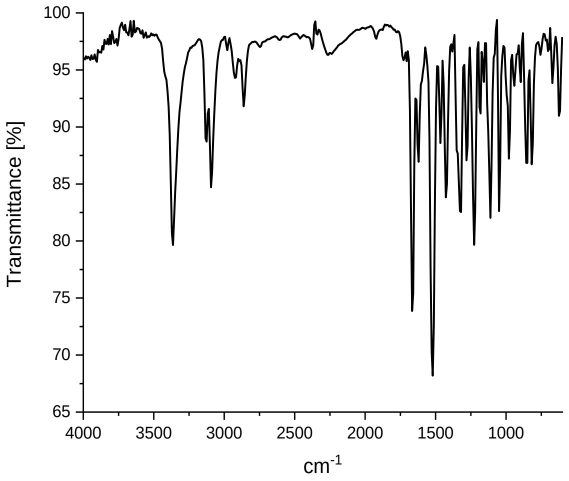

Transmittance [%]
100
95
90
85
80
75
70
65
4000
3500
3000
2500
2000
1500
1000
cm-1

S16. FT-IR stacking for aniline compounds (6-9)

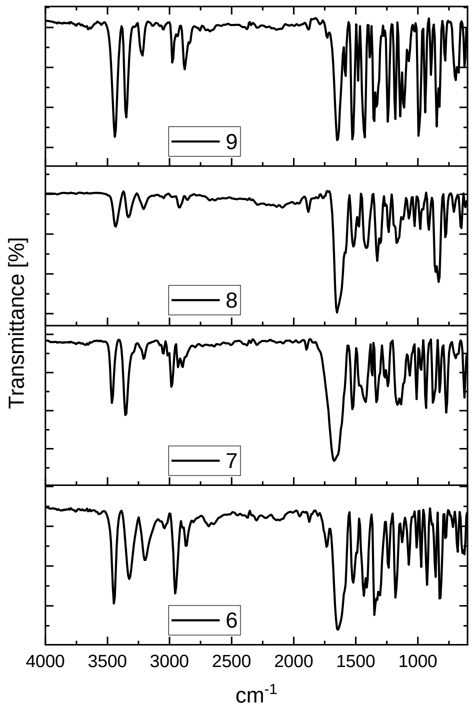

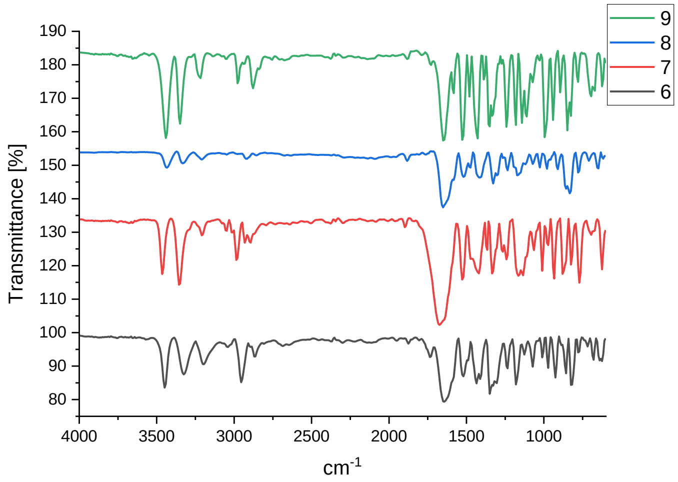

S17. FT-IR stacking for *N*-propargyl aniline compounds (11-14)

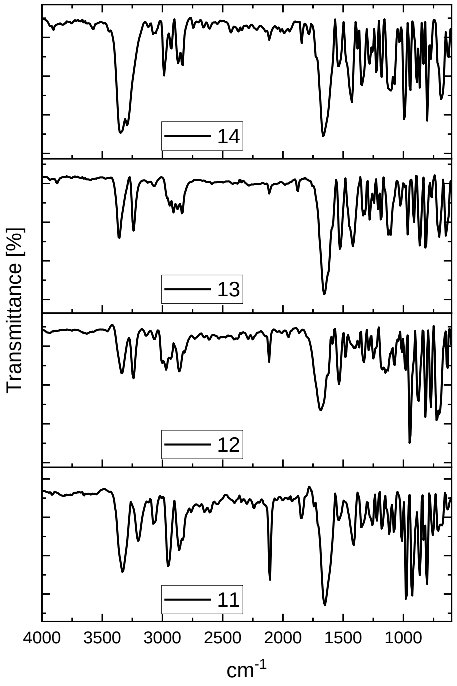

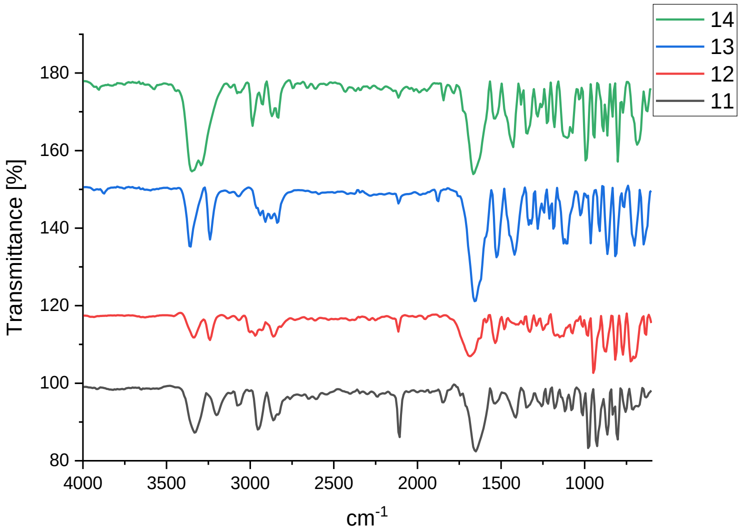

S18. FT-IR stacking for *N*-propargyl tetrahydroquinoline compounds (17-20)

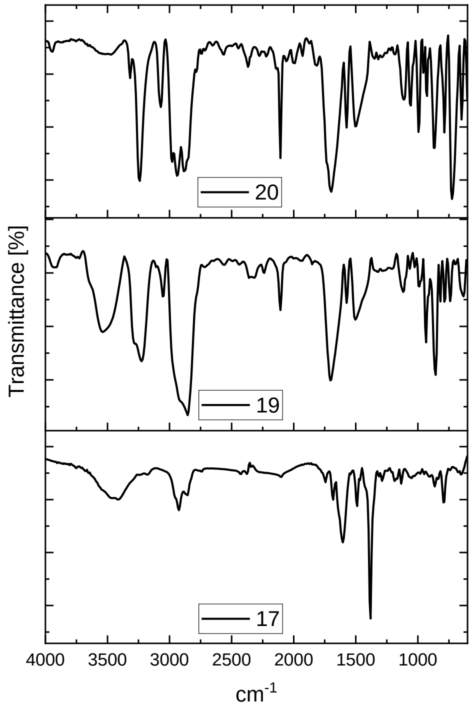

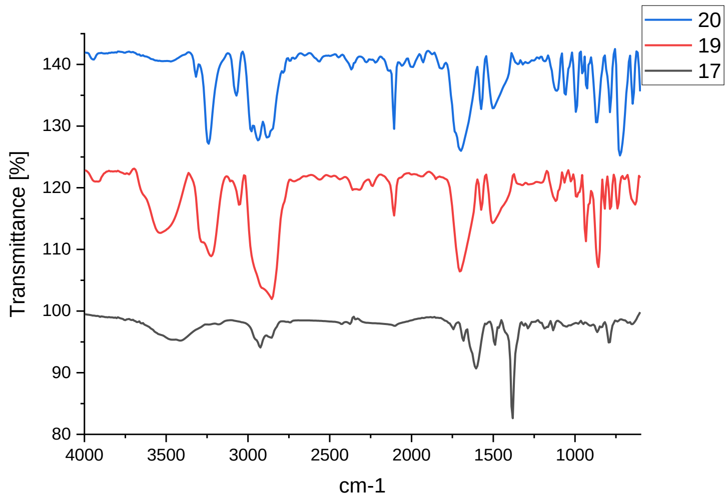

S19. FT-IR stacking for ¹H-1,2,3-triazole compounds (27,30,33,36)

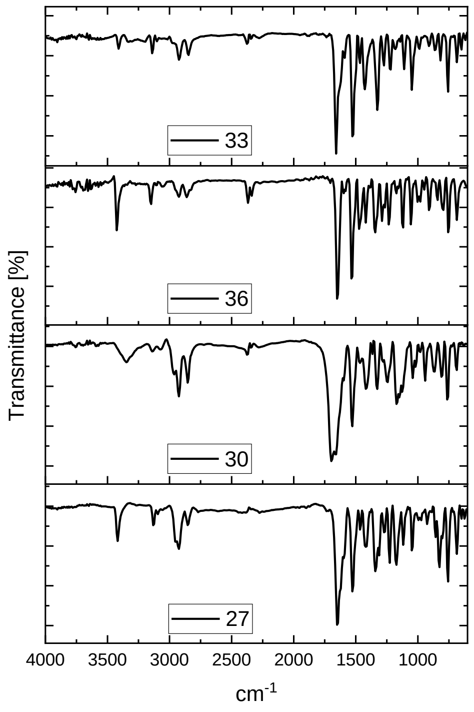

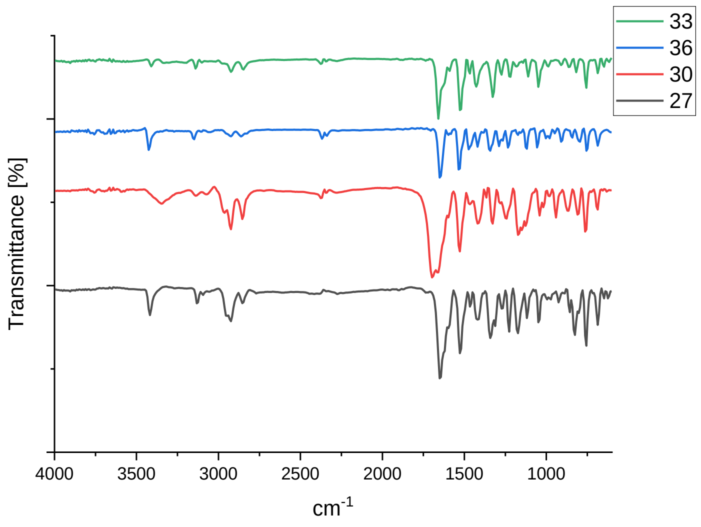

S20. FT-IR stacking for ¹H-1,2,3-triazole compounds (28,31,34,37)

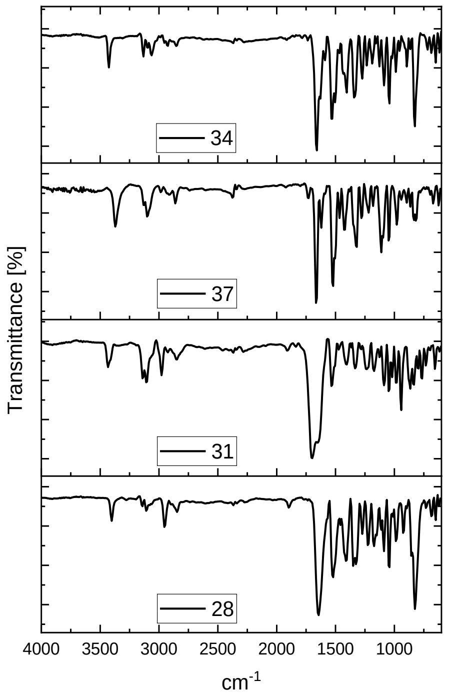

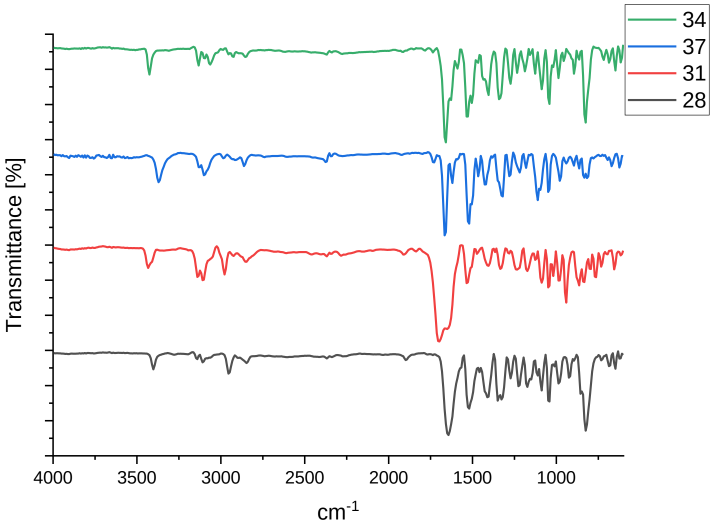

S21. FT-IR stacking for [1]H-1,2,3-triazole compounds (29,32,35,38)

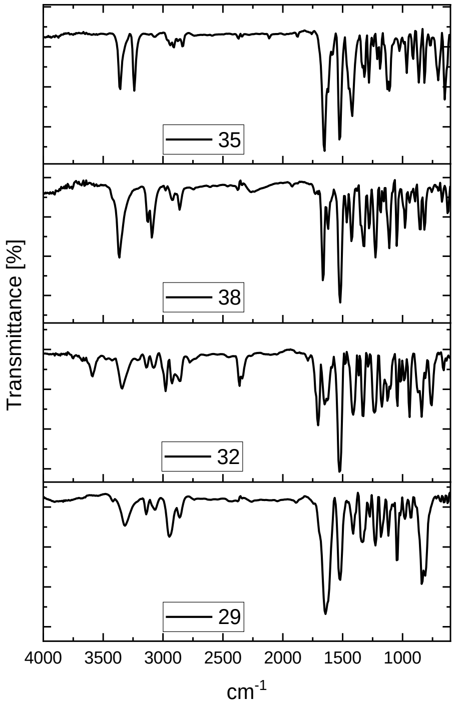

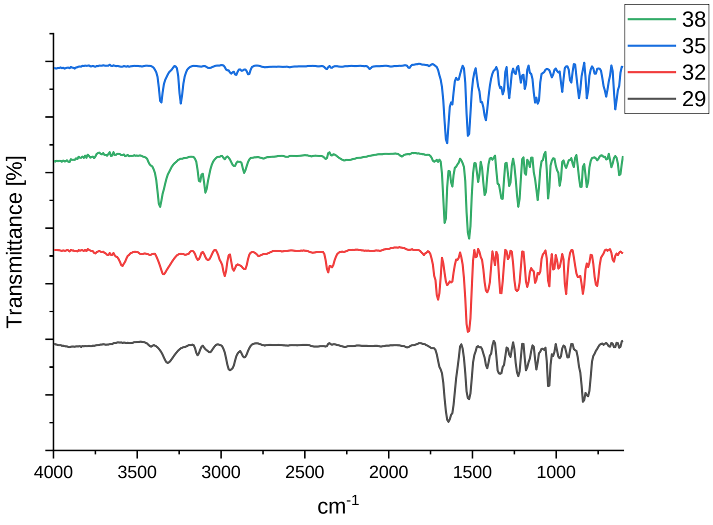

S22. Table of calculated log P of synthesized compounds (cont.)

Compound	log P[a]	Ilog P[b]	Xlog P3[b]	Wlog P[b]	Mlog P[b]	Silicos-IT[b]	Consensus[b]
**6**	0.84 ± 0.52	1.91	1.12	1.97	1.84	1.89	1.75
**7**	0.67 ± 0.65	2.69	1.39	1.66	1.54	1.08	1.67
**8**	0.35 ± 0.61	1.7	1.15	1.54	1.56	1.85	1.56
**9**	-0.50 ± 0.57	1.63	0.36	0.82	0.71	1.29	0.96
**11**	1.60 ± 0.56	2.71	1.90	2.32	2.56	2.84	2.47
**12**	1.42 ± 0.66	3.45	2.17	2.00	2.18	2.15	2.39
**13**	1.11 ± 0.63	2.22	1.93	1.88	2.3	2.79	2.22
**14**	0.25 ± 0.58	2.37	1.14	1.16	1.45	2.23	1.67
**17**	1.75 ± 0.71	3.39	1.57	1.88	2.86	3.11	2.56
**18**	1.26 ± 0.76	2.63	1.6	1.45	2.63	3.04	2.27
**19**	0.40 ± 0.73	3.12	0.8	0.73	1.82	2.48	1.79
**20**	2.58 ± 0.67	3.27	2.74	3.23	3.04	2.65	2.99
**27**	3.17 ± 0.68	3.56	3.37	3.89	3.53	3.28	3.52
**28**	2.63 ± 0.70	3.16	2.84	3.79	3.42	3.06	3.25
**29**	2.40 ± 0.87	4.19	3.01	2.92	2.68	1.95	2.95
**30**	3.00 ± 0.88	4.18	3.64	3.57	3.15	2.60	3.43
**31**	2.45 ± 0.91	4.05	3.11	3.48	3.05	2.38	3.21
**32**	2.09 ± 0.78	3.06	2.77	2.80	2.81	2.57	2.80
**33**	2.68 ± 0.80	3.34	3.40	3.45	3.31	3.21	3.34
**34**	2.14 ± 0.83	2.94	2.88	3.35	3.19	2.99	3.07
**35**	1.23 ± 0.69	3.19	1.98	2.08	2.01	2.01	2.25
**36**	1.83 ± 0.70	3.37	2.61	2.73	2.51	2.65	2.77
**37**	1.28 ± 0.74	3.11	2.08	2.64	2.39	2.43	2.53

[a]log P of synthesized compounds were calculated using ACD Labs log P predictor. [b] log P of synthesized compounds were calculated using SwissADME log P predictor

S23. Reaction optimization for the synthesis of compounds 7-9

Entry	Lactam (equiv.)	Aniline (equiv.)	CuI (equiv.)	DMEDA (equiv.)	T (°C)	Time (h)	Heating source	Yield (%)[1]	Yield (%)[2]	Yield (%)[3]
1	1.2	1	0.5	0.5	20	96	r.t.	12.74	9.51	15.29
2	1.2	1	0.5	0.5	100	72	conventional	39.5	23.16	40.41
3	1.2	1	0.5	0.5	120	48	conventional	25.48	28.87	45.37
4	1.2	1	0.5	0.5	60	6	sonication	28.55	30.82	62.81
5	1.2	1	0.05	0.1	120	2,0	microwave	6.68	0.91	15,0
6	1.2	1	0.1	0.2	120	2,0	microwave	31.6	3.86	29.67
7	1.2	1	0.15	0.3	120	2,0	microwave	34.87	8.45	52.79
8	1.2	1	0.25	0.5	120	2,0	microwave	39.5	23.16	60.69
9	1.2	1	0.5	0.5	120	2,0	microwave	79.69	62.31	86.91
10	1.2	1	0.5	0.5	160	1.5	microwave	**85.49**[4]	**73.3**	**88.74**
11	**1**	**1.2**	0.5	0.5	160	1.5	microwave	**90.0**[4]	**86.0**	**94.0**

[1]*N*-Boc piperazinone (**7**), [2]Thiomorpholinone (**8**), [3]Morpholinone (**9**), [4]Reaction temperature 90 °C.

S24. Dynamic RMSD for compound 19

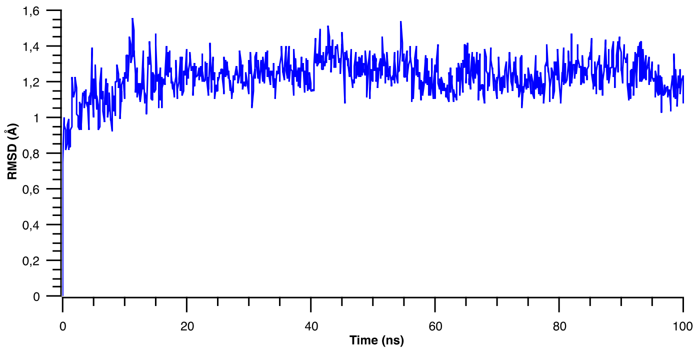

S25. ROCs AUC curve for method enrichment

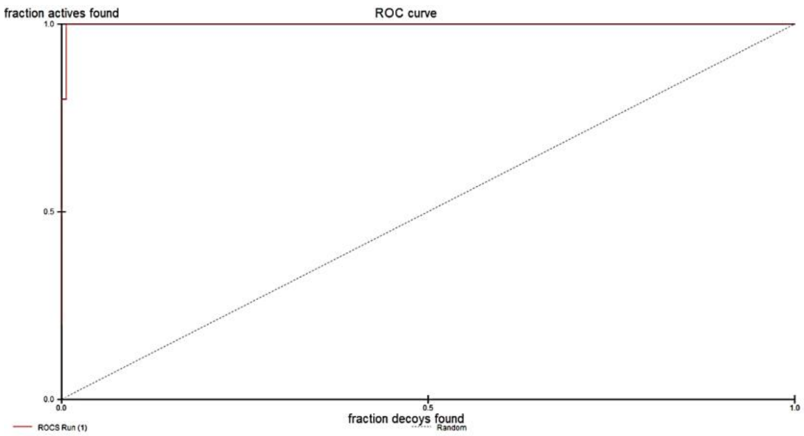

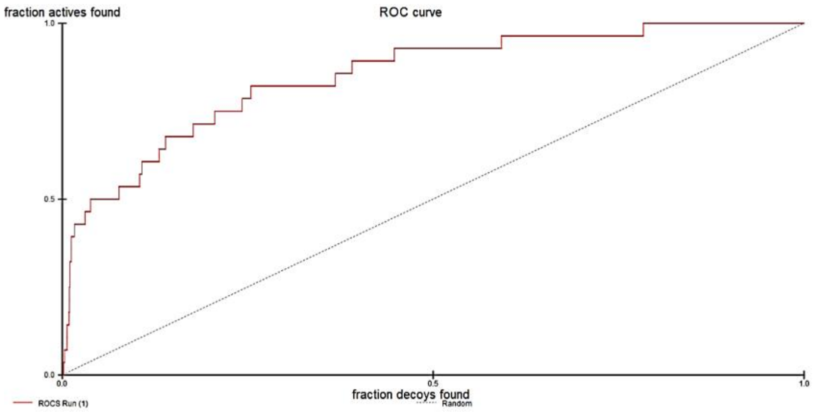

S26. Boiled egg diagram for blood brain-barrier

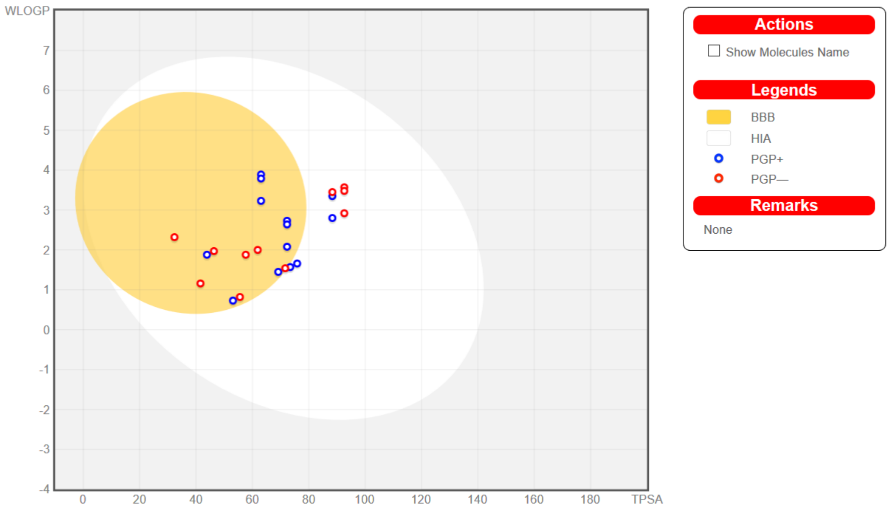

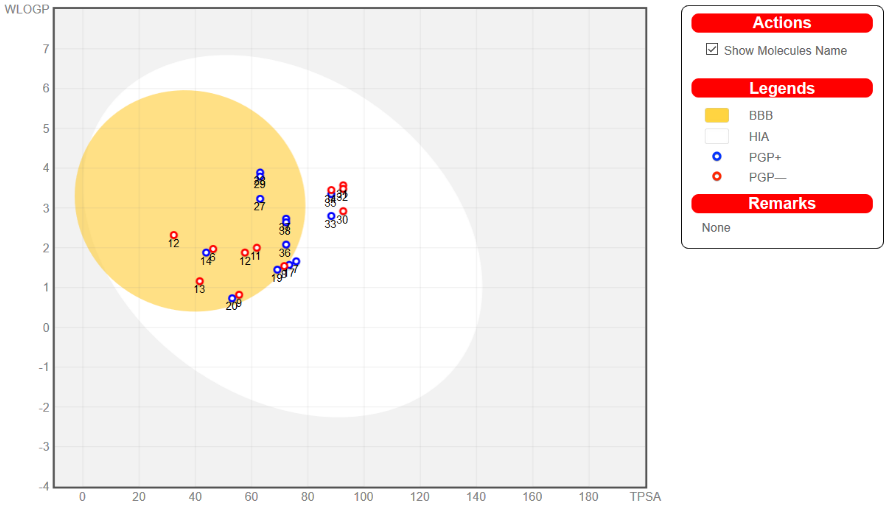

S27. Crystal data and structure refinement for compound 6

Identification code	6	
Empirical formula	C11H13FN2O	
Formula weight	208.23	
Temperature	100(2) K	
Wavelength	0.71073 Å	
Crystal system	Monoclinic	
Space group	P2(1/n)	
Unit cell dimensions	a = 5.6811(8) Å	α= 90°.
	b = 13.3981(19) Å	β= 97.192(5)°.
	c = 13.2740(18) Å	γ = 90°.
Volume	1002.4(2) Å3	
Z	4	
Density (calculated)	1.380 Mg/m3	
Absorption coefficient	0.103 mm-1	
F(000)	440	
Crystal size	0.12 x 0.11 x 0.10 mm3	
Theta range for data collection	2.17 to 26.41°.	
Index ranges	-7<=h<=7, -16<=k<=16, -16<=l<=16	
Reflections collected	28701	
Independent reflections	2050 [$R_{(int)}$ = 0.0472]	
Completeness to theta = 26.41°	99.8 %	
Max. and min. transmission	0.9900 and 0.9874	
Refinement method	Full-matrix least-squares on F^2	
Data / restraints / parameters	2050 / 50 / 199	
Goodness-of-fit on F^2	1.082	
Final R indices [I>2σ(I)]	R_1= 0.0461, wR_2 = 0.1292	
R indices (all data)	R_1= 0.0571, wR_2 = 0.1376	
Largest diff. peak and hole	0.488 and -0.262 e.Å-3	

S28. Crystal data and structure refinement for compound 9

Identification code	9	
Empirical formula	C10H11FN2O2	
Formula weight	210.21	
Temperature	100(2) K	
Wavelength	0.71073 Å	
Crystal system	Monoclinic	
Space group	C2/c	
Unit cell dimensions	a = 20.2264(16) Å	α= 90°.
	b = 6.3168(5) Å	β= 109.377(2)°.
	c = 16.1031(12) Å	γ = 90°.
Volume	1940.9(3) $Å^3$	
Z	8	
Density (calculated)	1.439 Mg/m^3	
Absorption coefficient	0.114 mm^{-1}	
F(000)	880	
Crystal size	0.27 x 0.21 x 0.13 mm^3	
Theta range for data collection	2.68 to 26.40°.	
Index ranges	-25<=h<=25, -7<=k<=7, -20<=l<=20	
Reflections collected	27057	
Independent reflections	1991 [$R_{(int)}$ = 0.0389]	
Completeness to theta = 26.40°	99.9 %	
Max. and min. transmission	0.9858 and 0.9698	
Refinement method	Full-matrix least-squares on F^2	
Data / restraints / parameters	1991 / 0 / 180	
Goodness-of-fit on F^2	0.789	
Final R indices [I>2σ(I)]	R_1= 0.0343, wR_2 = 0.0944	
R indices (all data)	R_1= 0.0394, wR_2 = 0.0995	
Largest diff. peak and hole	0.231 and -0.186 e.$Å^{-3}$	

S29. Crystal data and structure refinement for compound 20

Identification code	20	
Empirical formula	C21H21FN2O3	
Formula weight	368.40	
Temperature	100(2) K	
Wavelength	0.71073 Å	
Crystal system	Triclinic	
Space group	P-1	
Unit cell dimensions	a = 8.7304(9) Å	α= 77.885(3)°.
	b = 9.6126(10) Å	β= 71.698(3)°.
	c = 11.8765(12) Å	γ = 70.361(3)°.
Volume	885.15(16) $Å^3$	
Z	2	
Density (calculated)	1.382 Mg/m^3	
Absorption coefficient	0.100 mm^{-1}	
F(000)	388	
Crystal size	0.15 x 0.13 x 0.10 mm^3	
Theta range for data collection	1.82 to 26.46°.	
Index ranges	-10<=h<=10, -11<=k<=12, -14<=l<=14	
Reflections collected	32341	
Independent reflections	3639 [$R_{(int)}$ = 0.0429]	
Completeness to theta = 26.46°	99.8 %	
Max. and min. transmission	0.9902 and 0.9851	
Refinement method	Full-matrix least-squares on F^2	
Data / restraints / parameters	3639 / 0 / 244	
Goodness-of-fit on F^2	1.116	
Final R indices [I>2σ(I)]	R1 = 0.0518, wR2 = 0.1552	
R indices (all data)	R1 = 0.0619, wR2 = 0.1666	
Largest diff. peak and hole	0.864 and -0.299 e.$Å^{-3}$	

S30. Dihedral bond angle difference between calculated and experimental compound 9.

Calculated **Experimental**

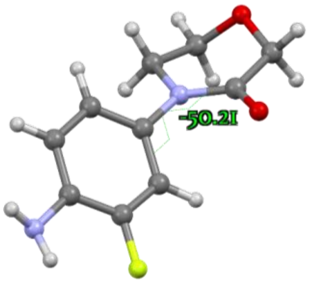

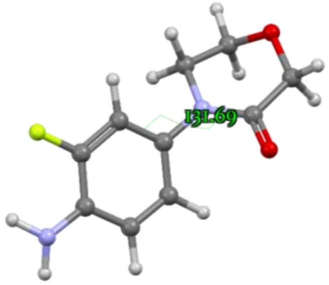

S31. Hydrogen bond formed in compound 9 crystal

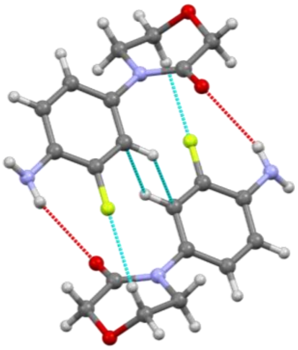

S32. Interactions between the propargyl and the carbonyl group in the crystal structure of compound 20

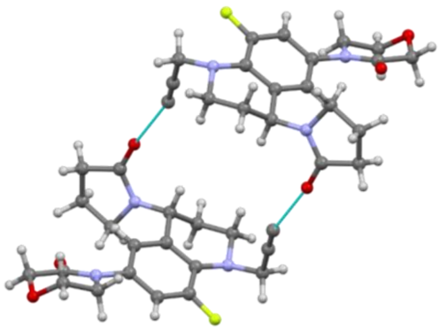

S33. Fukui functions for compound 14

Atom		Neutral	Cation	Anion	*f-*	*f+*	*f°*	Δ*f*
Nº	Type							
1	S	0.258944	0.353466	0.137422	0.094522	0.121522	0.108022	0.027000
2	C	-0.638921	-0.624136	-0.604985	0.014785	0.033936	0.024361	0.019151
3	C	-0.143918	-0.133043	-0.117730	0.010875	0.026188	0.018532	0.015313
4	N	-0.687981	-0.663278	-0.661368	0.024703	0.026613	0.025658	0.001910
5	C	0.576118	0.595208	0.485268	0.019090	0.090850	0.054970	0.071760
6	C	-0.748307	-0.714594	-0.732123	0.033713	0.016184	0.024949	-0.017529
7	C	0.351941	0.188277	0.322766	0.163664	0.029175	0.096420	-0.134489
8	C	-0.244880	-0.033686	-0.295819	0.211194	0.050939	0.131067	-0.160255
9	C	0.258751	0.231914	0.240765	0.026837	0.017986	0.022412	-0.008851
10	C	0.229809	0.364839	0.182387	0.135030	0.047422	0.091226	-0.087608
11	C	-0.121101	-0.123262	-0.151056	0.002161	0.029955	0.016058	0.027794
12	C	-0.112750	-0.033566	-0.114877	0.079184	0.002127	0.040656	-0.077057
13	F	-0.315098	-0.302832	-0.345970	0.012266	0.030872	0.021569	0.018606
14	N	-0.663792	-0.689837	-0.673497	0.026045	0.009705	0.017875	-0.016340
15	C	-0.208653	-0.175108	-0.194225	0.033545	0.014428	0.023987	-0.019117
16	O	-0.389878	-0.357438	-0.498669	0.032440	0.108791	0.070616	0.076351
17	C	0.229584	0.156303	0.257936	0.073281	0.028352	0.050817	-0.044929
18	C	-0.526177	-0.432097	-0.591886	0.094080	0.065709	0.079895	-0.028371
19	H	0.224413	0.246404	0.175686	0.021991	0.048727	0.035359	0.026736
20	H	0.230835	0.256244	0.185958	0.025409	0.044877	0.035143	0.019468
21	H	0.202515	0.221628	0.167322	0.019113	0.035193	0.027153	0.016080
22	H	0.225757	0.242181	0.168567	0.016424	0.057190	0.036807	0.040766
23	H	0.243868	0.280782	0.168761	0.036914	0.075107	0.056011	0.038193
24	H	0.256761	0.279102	0.206292	0.022341	0.050469	0.036405	0.028128
25	H	0.185274	0.231223	0.140703	0.045949	0.044571	0.045260	-0.001378
26	H	0.168206	0.221657	0.121542	0.053451	0.046664	0.050058	-0.006787
27	H	0.186011	0.203863	0.152611	0.017852	0.033400	0.025626	0.015548
28	H	0.315077	0.404017	0.289489	0.088940	0.025588	0.057264	-0.063352
29	H	0.210411	0.271672	0.174992	0.061261	0.035419	0.048340	-0.025842
30	H	0.225779	0.276459	0.208341	0.050680	0.017438	0.034059	-0.033242
31	H	0.221403	0.257637	0.195397	0.036234	0.026006	0.031120	-0.010228

S34. Fukui functions for compound 25

Atom		Neutral	Cation	Anion	*f*-	*f*+	*f*°	Δ*f*
Nº	Type							
1	C	-0.080679	-0.024180	-0.113052	0.056499	0.032373	0.044436	-0.024126
2	C	-0.149657	-0.117282	-0.162486	0.032375	0.012829	0.022602	-0.019546
3	C	-0.138827	-0.051506	-0.202111	0.087321	0.063284	0.075303	-0.024037
4	C	-0.149657	-0.117282	-0.162486	0.032375	0.012829	0.022602	-0.019546
5	C	-0.080679	-0.024180	-0.113052	0.056499	0.032373	0.044436	-0.024126
6	C	0.025736	0.084568	0.023830	0.058832	0.001906	0.030369	-0.056926
7	N	-0.166497	-0.074790	-0.242773	0.091707	0.076276	0.083992	-0.015431
8	N	0.017948	0.037180	-0.122350	0.019232	0.140298	0.079765	0.121066
9	N	-0.071665	0.080270	-0.295994	0.151935	0.224329	0.188132	0.072394
10	H	0.160865	0.240213	0.076127	0.079348	0.084738	0.082043	0.005390
11	H	0.158119	0.239545	0.081440	0.081426	0.076679	0.079053	-0.004747
12	H	0.156008	0.247687	0.075340	0.091679	0.080668	0.086174	-0.011011
13	H	0.158119	0.239545	0.081440	0.081426	0.076679	0.079053	-0.004747
14	H	0.160865	0.240213	0.076127	0.079348	0.084738	0.082043	0.005390

S35. Reaction species according to Fukui calculations

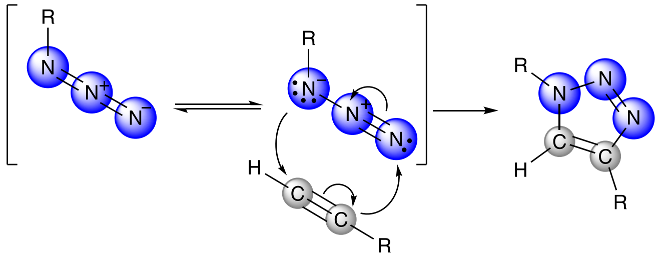

S36. Calculated thermochemical energies

Compound	E (hartree)	E (eV)	E (kcal/mol)	E (kJ/mol)	ΔH (kcal/mol)	ΔH (kJ/mol)
**12**	-826.73	-22496.89	-518779.39	-2170571.43	-	-
**13**	-1188.45	-32340.04	-745762.91	-3120269.84	-	-
**14**	-1185.62	-32263.15	-743989.80	-3112851.15	-	-
**15**	-862.63	-23474.02	-541312.09	-2264848.18	-	-
**29**	-1222.51	-33266.98	-767138.05	-3209703.34	-61.76	-258.39
**32**	-1584.23	-43110.13	-994121.74	-4159402.43	-61.92	-259.07
**35**	-1581.41	-43033.25	-992348.76	-4151984.31	-62.06	-259.64
**38**	-1258.42	-34244.11	-789670.70	-3303979.91	-61.71	-258.22
**30**	-1682.12	-45773.94	-1055549.11	-4416414.36	-61.58	-257.65
**33**	-2043.84	-55617.09	-1282532.70	-5366113.04	-61.64	-257.92
**36**	-2041.02	-55540.20	-1280759.71	-5358694.86	-61.77	-258.43
**39**	-1718.03	-46751.06	-1078081.71	-4510690.70	-61.48	-257.23
**31**	-1321.76	-35967.86	-829420.50	-3470292.93	-61.56	-257.57
**34**	-1683.49	-45811.01	-1056404.14	-4419991.81	-61.67	-258.04
**37**	-1680.66	-45734.13	-1054631.13	-4412573.55	-61.78	-258.47
**40**	-1357.67	-36944.99	-851953.11	-3564569.31	-61.47	-257.20
**25**	-395.69	-10767.41	-248296.90	-1038873.52	-	-
**26**	-494.94	-13468.30	-310579.55	-1299463.93	-	-
**27**	-855.30	-23274.37	-536708.14	-2245585.28	-	-
